# Double‐Pronged NAD Preservation: Delaying Cellular Senescence and Initiating Musculoskeletal Regeneration

**DOI:** 10.1111/acel.70468

**Published:** 2026-04-07

**Authors:** Jianfeng Yu, Mingzhuang Hou, Yaoge Deng, Chenqi Yu, Yang Liu, Kang Kang, Xiaowei Xia, Xiaoping Li, Huilin Yang, Dinghua Jiang, Wu Xu, Yijian Zhang, Xuesong Zhu

**Affiliations:** ^1^ Department of Orthopaedics, The First Affiliated Hospital of Soochow University Soochow University Suzhou China; ^2^ Orthopaedic Institute, Medical College Soochow University Suzhou China; ^3^ Department of Orthopaedics Qilu Hospital of Shandong University Jinan China

**Keywords:** apigenin, cellular senescence, musculoskeletal disorders, nicotinamide adenine dinucleotide, nicotinamide mononucleotide, phytosphingosine

## Abstract

In the context of population aging, musculoskeletal fitness has emerged as a cornerstone of overall well‐being and injury prevention, relying on the coordinated function of cartilage, bone, and muscle. Drawing on the principle of “increasing income and reducing expenditure,” we propose a combinatorial formulation consisting of the nicotinamide adenine dinucleotide (NAD) precursor nicotinamide mononucleotide (NMN) and the NAD^+^‐consuming enzyme inhibitor apigenin (API), hereafter referred to as the “N + A” regimen, to enhance NAD^+^ reserves. Our results revealed that the N + A formulation alleviated cellular senescence, thereby promoting the differentiation of skeletal precursor cells into chondrocytes, osteoblasts, and myocytes for the reconstruction of the musculoskeletal system. Oral administration of the N + A formulation alleviated cartilage degeneration, bone loss, and muscle atrophy; additionally, it enhanced exercise capacity in aged mice. Mechanistically, the N + A strategy preserves NAD^+^ levels, which are subsequently utilized by mitochondrial sirtuin 3 (SIRT3) to promote deacetylation modifications and alleviate the senescent phenotype. Moreover, oral administration of N + A indirectly enhanced the synthesis of the metabolite phytosphingosine (PHS) by the intestinal microbiota members *Coriobacteriaceae_UCG‐002* and *Ruminococcus*, thereby alleviating age‐related degeneration. In summary, our findings demonstrate that enhancing the NAD^+^ reservoir represents a promising strategy for promoting musculoskeletal regeneration, and we developed a rational combinatorial regimen with potential for clinical translation.

## Graphical abstract

A novel drug combination (NMN + API) promotes musculoskeletal regeneration in aging. By boosting NAD^+^, it activates SIRT3 to suppress cellular senescence and enhance differentiation into chondrocytes, osteoblasts, and myocytes. Oral administration reshapes the gut microbiota, increasing production of the anti‐aging metabolite PHS.

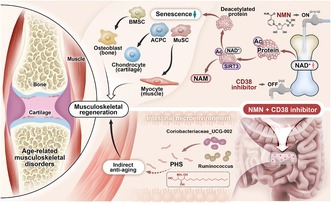

## 1 Introduction

With global population aging emerging as one of the most significant demographic shifts in the modern era, investigations into age‐related disorders have become a critical public health priority (Yang et al. 2025). The musculoskeletal system is among the most significantly affected systems during organismal aging (Zhang, Zhou, et al. 2024). Accumulating evidence indicates that advancing age is closely associated with progressive bone loss (osteoporosis, OP) (Xu et al. 2023), cartilage degeneration (osteoarthritis, OA) (Hu et al. 2025), and skeletal muscle atrophy (sarcopenia) (Lai et al. 2024). Enhancing musculoskeletal function through regular exercise has been shown to exert geroprotective effects, attenuating age‐related functional decline across multiple tissues and organs (Geng et al. 2025). Owing to the complexity of the musculoskeletal system, the relationships among cartilage, bone, and muscle have garnered significant attention (Yan et al. 2024). Muscle‐derived extracellular vesicles (EV) transport LDHA, enhancing glycolysis in bone marrow‐derived mesenchymal stem cells (BMSC), thereby promoting osteogenesis (Ma et al. 2023). Osteocyte‐derived EV induce dysregulation of cartilage metabolism via the miR‐23b‐3p‐OTUD4 axis during development of OA (Liu et al. 2025). Combined therapeutic strategies targeting both muscle atrophy and OA through the involvement of coenzyme Q10 to suppress ferroptosis have been shown to exhibit therapeutic efficacy (Xiang et al. 2025). In our previous study, we engineered anisotropic bicellular living hydrogels capable of facilitating spatiotemporal, cell‐driven regeneration for the reconstruction of the cartilage‐bone interface (Zhang, Li, et al. 2024). Therefore, future research should focus more on developing integrated therapeutic strategies capable of achieving multiple objectives—such as restoring multi‐tissue homeostasis and enhancing musculoskeletal regeneration—within a single intervention.

Aging is characterized by a progressive decline in coenzyme availability, with nicotinamide adenine dinucleotide (NAD^+^) playing a significant role in regulating senescent phenotypes through multiple signaling pathways (Wiley and Campisi 2016). Notably, NAD^+^ is synthesized via three primary pathways: the de novo biosynthesis pathway, the Preiss‐Handler pathway, and the salvage pathway, with the latter serving as the most important route for NAD^+^ production (Brenner 2025). Within the salvage pathway, nicotinamide (NAM) and nicotinamide riboside (NR) are converted into nicotinamide mononucleotide (NMN) by NAMPT and NRK, respectively. Subsequently, NMN is converted into NAD by NMNAT (Migaud et al. 2024). Research has shown that supplementation with these precursors leads to an increase in NAD^+^ levels, ultimately improving aging‐related cardiopulmonary dysfunction and reducing mortality risk (Abdellatif et al. 2021; Norheim et al. 2024). Importantly, NMN serves as a co‐product of NR and NAM and is readily converted into NAD^+^ by NMNAT. The most recent study has demonstrated that solely NMN and NR, rather than NAM, are capable of increasing the circulatory NAD^+^ concentrations in healthy adults (Christen et al. 2026). Moreover, NMN seems to be a more direct NAD^+^ precursor compared to NR, which can enter cells directly via SLC12A8 and contribute to the synthesis of NAD^+^ (Grozio et al. 2019). Studies have shown that administration of NMN restores NAD^+^ levels, alleviates vascular aging, and improves muscle insulin sensitivity among prediabetic women (Yoshino et al. 2021; Zhan et al. 2023). Besides NAD^+^ biosynthesis, various NAD^+^‐consuming enzymes—such as sirtuins, poly‐ADP‐ribose polymerases (PARP), and CD38 enzyme (Chini et al. 2020)—play key roles in the regulation of NAD^+^ metabolism (Chini et al. 2021). CD38 exhibits a higher level of sensitivity to the aging process and assumes a crucial role in the age‐associated reduction of NAD^+^ (Camacho‐Pereira et al. 2016). Meanwhile, apigenin (API), a common and readily accessible flavonoid found in various natural sources, is also recognized as the specific pharmacological inhibitor of CD38 (Escande et al. 2013). CD38 deficiency alleviates the intracellular decline of NAD^+^ levels, thereby reversing vascular senescence and promoting vascular remodeling (Gan et al. 2021). Pharmacological inhibition of CD38 has been demonstrated to consistently mitigate ovarian aging and improve fertility in middle‐aged mice (Yang et al. 2024).

**SCHEME 1:**
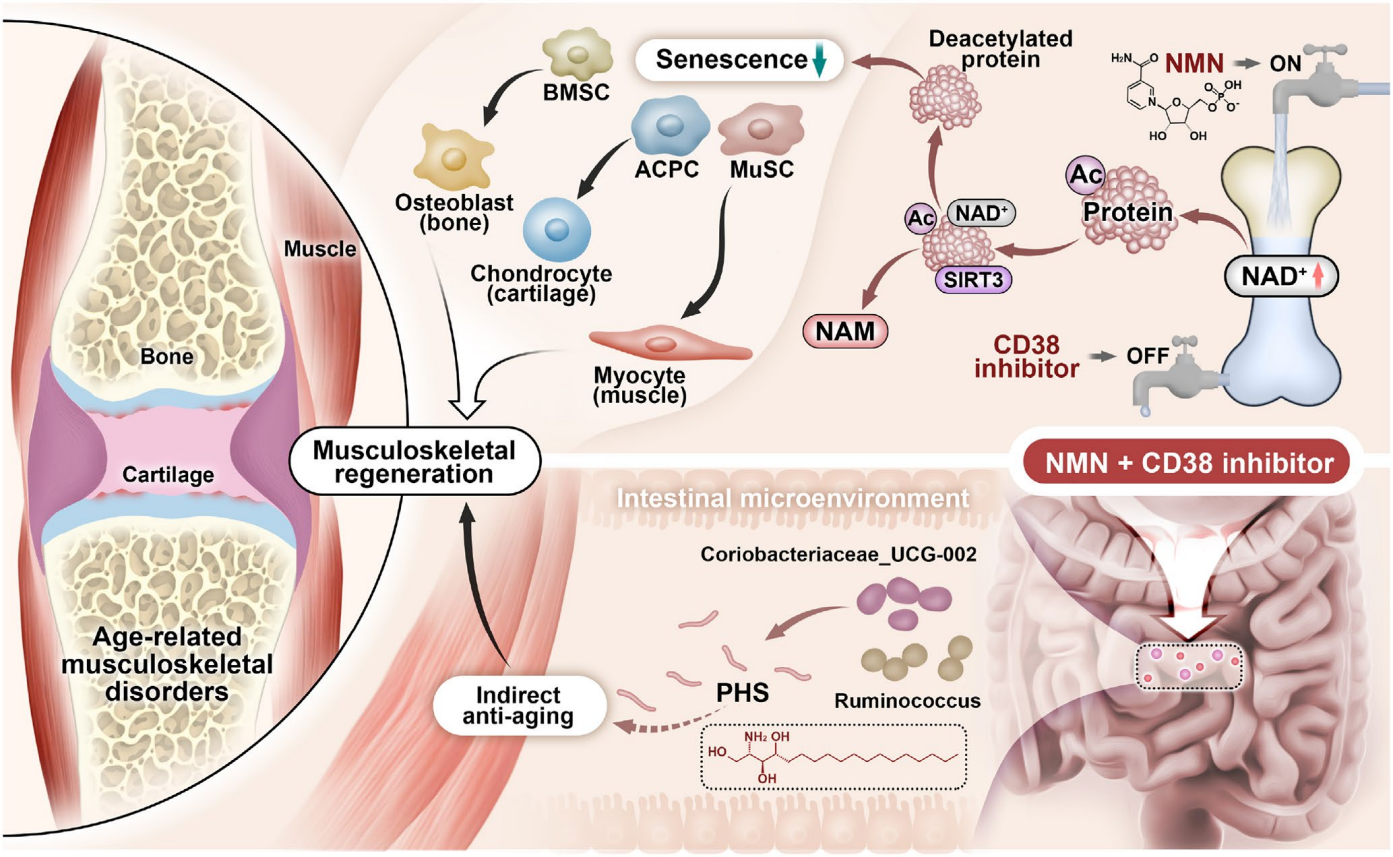
A novel synergistic drug combination (N + A) consisting of an NAD^+^ precursor (NMN) and an NAD^+^ consumption (CD38) inhibitor (API) promotes musculoskeletal regeneration in aging. Notably, increased NAD^+^ serves as a coenzyme for SIRT3, exerting a robust anti‐senescence effect, thus promoting tri‐lineage differentiation into chondrocytes, osteoblasts, and myocytes. Furthermore, oral administration of the N + A formulation modulated the intestinal microenvironment, promoting the gut microbiota‐derived production of the metabolite PHS, thereby exerting indirect anti‐aging effects in musculoskeletal disorders.

Thus, given its significant involvement in both aging and age‐related diseases, this study explored NAD^+^‐targeted therapeutic strategy for its potential in treating geriatric musculoskeletal disorders. Initially, NAD^+^ metabolism and associated alterations in NAD^+^ levels within the cartilage, bone, and muscle tissues were assessed using single‐cell RNA sequencing technology and corroborated across multiple aging models. Subsequently, a novel formulation (N + A), comprising NAD^+^ precursor NMN to enhance NAD^+^ biosynthesis and the CD38 natural inhibitor API to minimize NAD^+^ consumption—both for maintaining NAD^+^ pool—was administered to musculoskeletal precursor cells to evaluate its potential effects on cellular senescence and tri‐lineage differentiation capacity (Wu et al. 2022). Additionally, N + A was orally administered to aged mice in vivo to evaluate its therapeutic effects on OA, OP, and sarcopenia. To investigate the underlying molecular mechanisms of N + A, the role of NAD‐dependent deacetylase sirtuin 3 (SIRT3) was explored using CRISPR‐Cas9‐generated knockout mice. Additionally, following oral administration, the effects of the N + A formulation on the gut microbiota (GM) and associated metabolites were evaluated using 16S rRNA microbiota sequencing and fecal microbiota transplantation (FMT). Ultimately, we identified a novel candidate metabolite, phytosphingosine (PHS), and validated its potential as a therapeutic agent for musculoskeletal degeneration associated with aging. Collectively, this study proposes a two‐pronged strategy to reverse cellular senescence for musculoskeletal regeneration and identifies two distinct underlying mechanisms involving the NAD^+^‐dependent deacetylase SIRT3 and the gut metabolite PHS (Scheme 1).

## 2 Results

### 2.1 Aged Musculoskeletal System Was Closely Associated With Impaired NAD ^+^ Metabolism and a Decline in NAD ^+^ Levels

**FIGURE 1:**
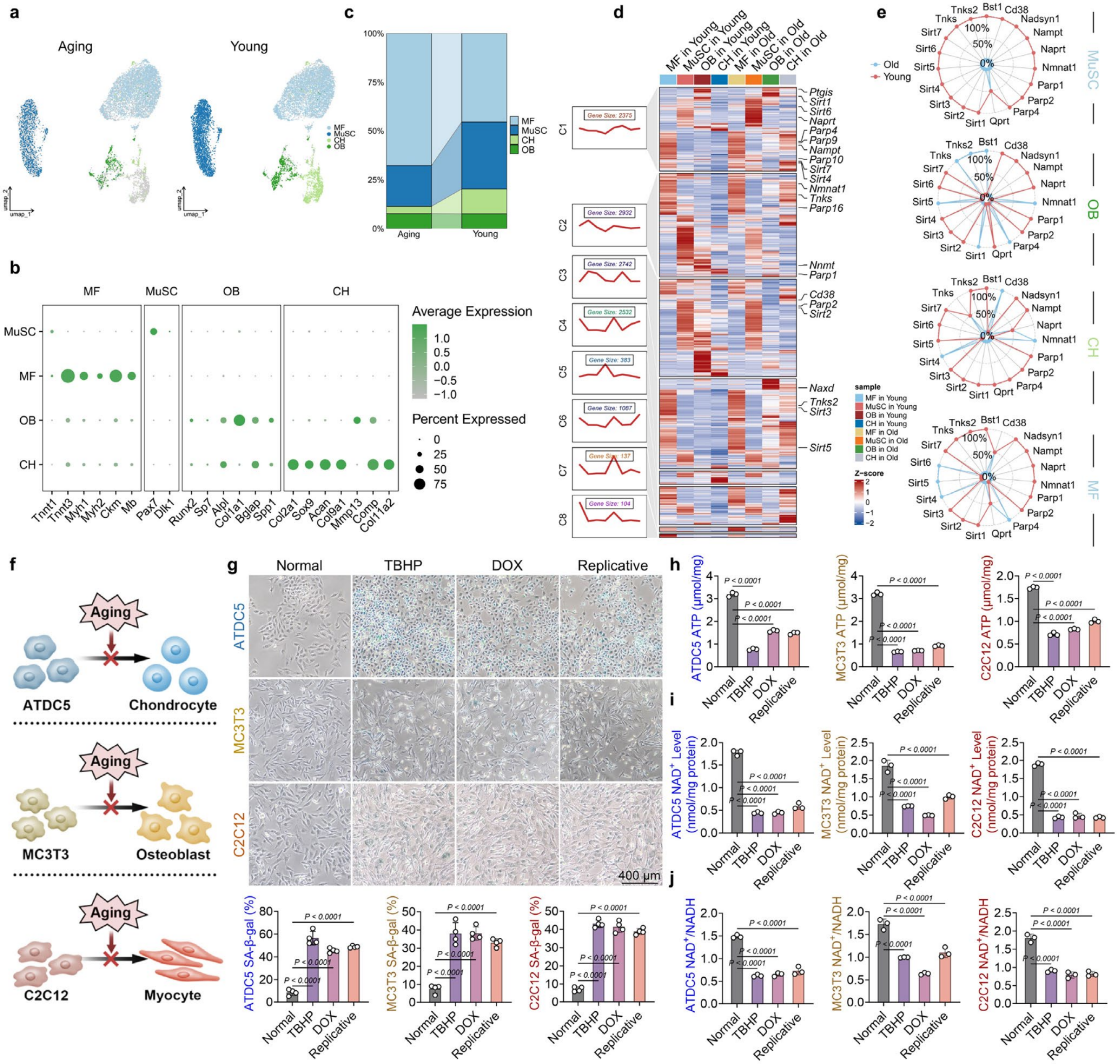
Aged musculoskeletal system was closely associated with impaired NAD metabolism and a decline in NAD levels. (a) The uniform manifold approximation and projection (UMAP) plot illustrating the four main clusters identified in 16‐ to 19‐month‐old aged muscle and femur tissues. (b) Dot plot depicting the mean expression levels of preferentially expressed genes across the four subclusters. (c) Relative proportion of each cluster in aging versus young tissues. (d) Heatmap displaying differentially expressed genes. (e) Radar chart representing the cell‐type distribution of specifically expressed genes. (f) Aging suppresses the differentiation of ATDC5, MC3T3, and C2C12 cells. (g) Representative images and quantification of SA‐β‐gal staining (*n* = 3). (h) Impact of aging on ATP levels in ATDC5, MC3T3, and C2C12 cells (*n* = 3). (i) Influence of aging on NAD^+^ level in ATDC5, MC3T3, and C2C12 cells (*n* = 3). (j) Effect of aging on the NAD^+^/NADH ratio in ATDC5, MC3T3, and C2C12 cells (*n* = 3). Data are presented as mean ± SD; statistical significance was assessed using one‐way ANOVA, with significant differences between groups indicated by *p* < 0.05.

Age‐related changes and NAD^+^ deficiency play a significant role in determining the efficacy of cellular therapy (Hope et al. 2025). However, the metabolic alterations in NAD^+^ levels within the musculoskeletal system during the aging process remain incompletely understood. To address this knowledge gap, we performed a single‐cell transcriptomic analysis using data from publicly accessible databases (Kedlian et al. 2024; Zhong et al. 2020). Notably, to evaluate alterations in the musculoskeletal system under aging conditions, four cell populations were identified: myofibers (MF), muscle stem cells (MuSC), chondrocytes (CH), and osteoblasts (OB) (Figure 1a,b and Figure S1). Differential analysis results revealed a decreased proportion of precursor MuSCs within the aging sample, whereas the proportion of adult MFs was increased, indicating a decline in regenerative capacity with advancing age (Figure 1c). Cluster heatmaps revealed that NAD^+^ metabolism‐related genes, including NAMPT, NMNAT1, sirtuins, PARPs, and CD38 (Aksoy et al. 2006), demonstrated significant differences between young and aging samples (Figure 1d and Figure S2). Particularly, the expression of most NAD^+^‐related genes and signaling pathways was downregulated in aging tissues (Figure 1e and Figure S3). Subsequently, we examined the effects of aging on musculoskeletal tri‐lineage differentiation, including chondrogenesis, osteogenesis, and myogenesis, as well as on NAD^+^ metabolism (Figure 1f and Figure S4). Under three different aging models—oxidative stress induced by tert‐butyl hydroperoxide (TBHP), DNA damage due to doxorubicin (DOX), and replicative senescence observed in late‐passage cells—the proportion of β‐galactosidase (β‐gal)‐positive senescent musculoskeletal precursor cells was significantly increased (Figure 1g). Lineage differentiation is an energy‐dependent process with adenosine triphosphate (ATP) serving as its primary energy source (Powell 2018). Results from quantitative analyses supported that aging leads to a reduction in the concentration of cellular ATP, resulting in an energy deficit (Figure 1h). NAD^+^ exists in two interconvertible forms: the reduced form (NADH) and the oxidized form (NAD^+^). The oxidation of NADH to NAD involves the loss of an electron, a process tightly coupled to cellular energy generation (Cahoon and Freitag 2018). For cellular NAD^+^ and NADH metabolism, aging not only reduces the concentration of NAD^+^ but also decreases the NAD^+^/NADH ratio, with an unspecified alteration in NADH levels (Figure 1i,j and Figure S5). Collectively, these findings demonstrate that musculoskeletal aging was correlated with dysregulation in NAD^+^ metabolism and decreased NAD^+^ levels.

### 2.2 The N + A Formulation Alleviated Musculoskeletal Cellular Senescence and Restored Mitochondrial Function

**FIGURE 2:**
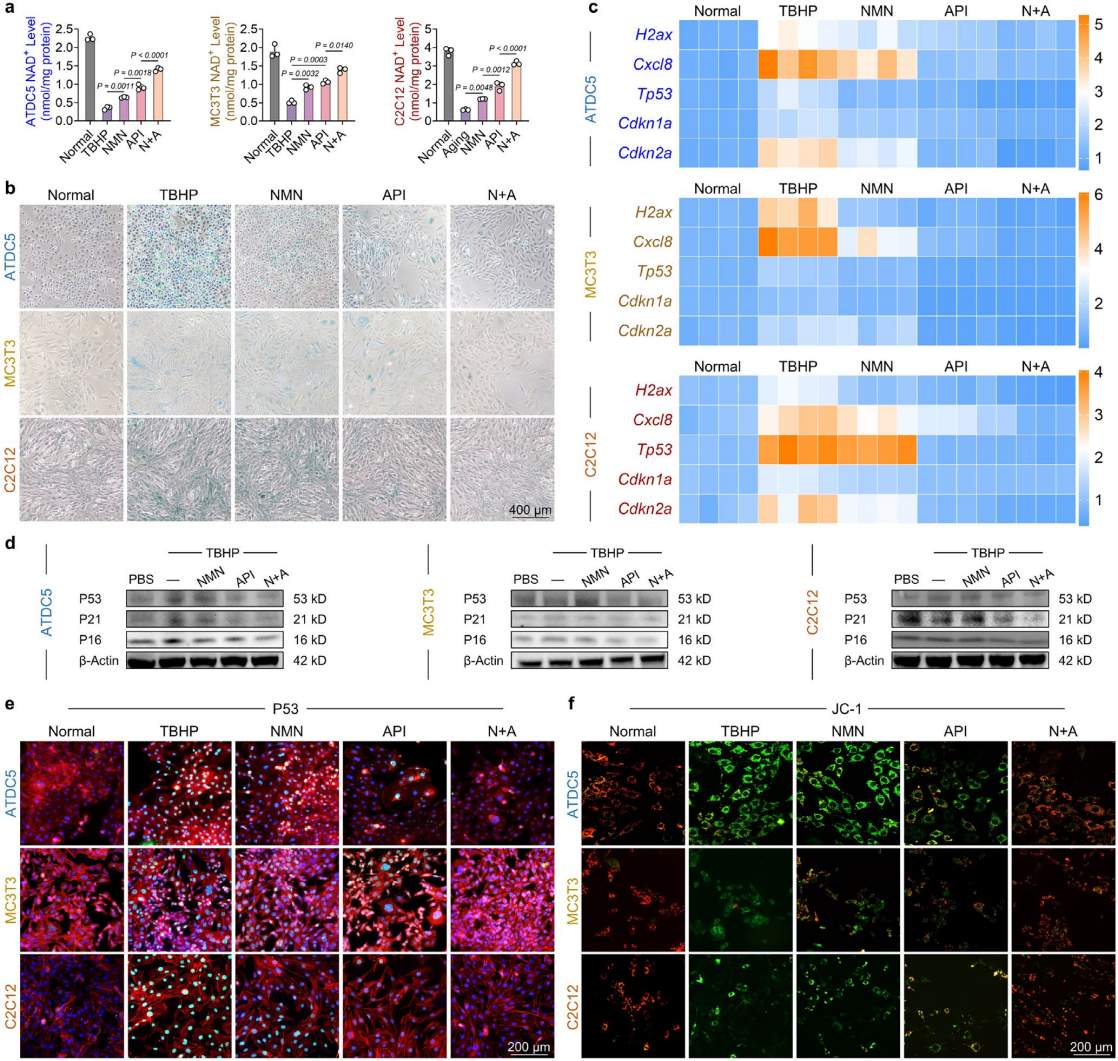
The N + A formulation alleviated musculoskeletal cellular senescence and restored mitochondrial function. (a) The effects of NMN and API on NAD^+^ levels in senescent cells (*n* = 3). (b) Representative images of SA‐β‐gal staining in ATDC5, MC3T3, and C2C12 cells following exposure to NMN and API. (c) Heatmaps depicting the expression levels of multiple senescence‐related genes (*H2ax*, *Cxcl8*, *Tp53*, *Cdkn1a*, *Cdkn2a*) in three cell types (ATDC5, MC3T3, C2C12) treated with NMN and API (Table S1). (d) Western blot analysis of P16, P21, and P53 protein expression following pharmacological intervention under three cellular senescence conditions (*n* = 3). (e) Cellular immunofluorescence detection of P53 following drug treatment in senescent cells. (f) Representative images of mitochondrial membrane potential (JC‐1) staining. Data are presented as mean ± SD; statistical significance was assessed using one‐way ANOVA, with significant differences between groups indicated by *p* < 0.05.

Given the observed NAD^+^ deficiency and impaired energy production in musculoskeletal precursor cells, we aimed to investigate a viable strategy to enhance cellular NAD^+^ metabolism. Building on the concept of simultaneously enhancing NAD^+^ biosynthesis (“increasing income”) and limiting its degradation (“reducing expenditure”), we developed a novel formulation comprising the NAD^+^ precursor NMN and the NAD^+^‐consuming enzyme inhibitor API, hereafter referred to as the “N + A” regimen. The live/dead cell assays demonstrated that treatment with NMN, API, or the combined N + A formulation prevented cell death under aging conditions (Figure S6). Regarding NAD^+^ homeostasis, both NMN and API partially restored the TBHP‐induced decline in NAD^+^ levels; however, the combined N + A formulation demonstrated the most pronounced enhancement of both NAD^+^ levels and the NAD^+^/NADH ratio across all the three types of musculoskeletal precursor cells (Figure 2a and Figure S7). Moreover, the in vivo assay of NAD metabolism in cartilage, bone, and muscle samples derived from differently treated mice indicated that the N + A formulation enhanced the NAD^+^ levels and the NAD^+^/NADH ratio (Figure S8). Owing to the importance of CD38, a NAD‐consuming enzyme, the expression level and activity of CD38 were examined in three cell lines (Aksoy et al. 2006). Our results indicated that the TBHP led to an increase in the expression and activity of CD38, and this phenomenon was mitigated by the treatment with N + A (Figure S9). Both the dose‐gradient and time‐gradient assays indicated that API treatment can effectively decrease the expression level and enzyme activity of CD38 in the three musculoskeletal cells (Figures S10 and S11). Furthermore, to investigate the relationship between apigenin and CD38, we conducted a molecular docking assay and found that CD38 is a potential target of apigenin (Figure S12). Additionally, the SA‐β‐galactosidase staining assay revealed that the NAD^+^ reservoir generated by the N + A formulation effectively reduced the proportion of senescent cells among the musculoskeletal precursor cells (Figure 2b and Figure S13). Notably, senescent cells secrete a wide range of bioactive molecules, collectively termed the senescence‐associated secretory phenotype (SASP), which can inhibit cell proliferation and disrupt normal differentiation processes (Wang et al. 2024). Heatmap analysis demonstrated that treatment with the N + A formulation suppressed SASP components such as the marker of double‐strand breaks γ‐H2ax and pro‐inflammatory chemokine CXCL8 (Figure 2c). Stress factors activate the DNA damage response (DDR) pathway, subsequently initiating either the classical P53‐P21^CIP1^‐CDK2 or the P16^INK4A^‐CDK4/6 signaling pathways, resulting in cell cycle arrest and cellular senescence (Groelly et al. 2023). Western blot and immunofluorescence staining demonstrated that expression levels of P53, P21, and P16 within the nuclear were significantly reduced following treatment with N + A (Figure 2d,e and Figures S14 and S15). Given that mitochondrial dysfunction is one of the primary triggers of cellular senescence, we further investigated the effects of the N + A formulation on mitochondrial function across the three types of precursor cells (Gallage and Gil 2016). Treatment with N + A formulation restored the impaired mitochondrial membrane potential (MMP) and promoted mitochondrial ATP biosynthesis (Figure 2f and Figure S16). Furthermore, N + A treatment effectively increased both the basal and maximal respiration rates in aged cells and significantly enhanced mitochondrial ATP production (Figure S17). Collectively, these findings demonstrate that treatment with the N + A formulation delayed cellular aging and restored mitochondrial function.

### 2.3 The N + A Formulation Simultaneously Enhanced Chondrogenic, Osteogenic, and Myogenic Differentiation

**FIGURE 3:**
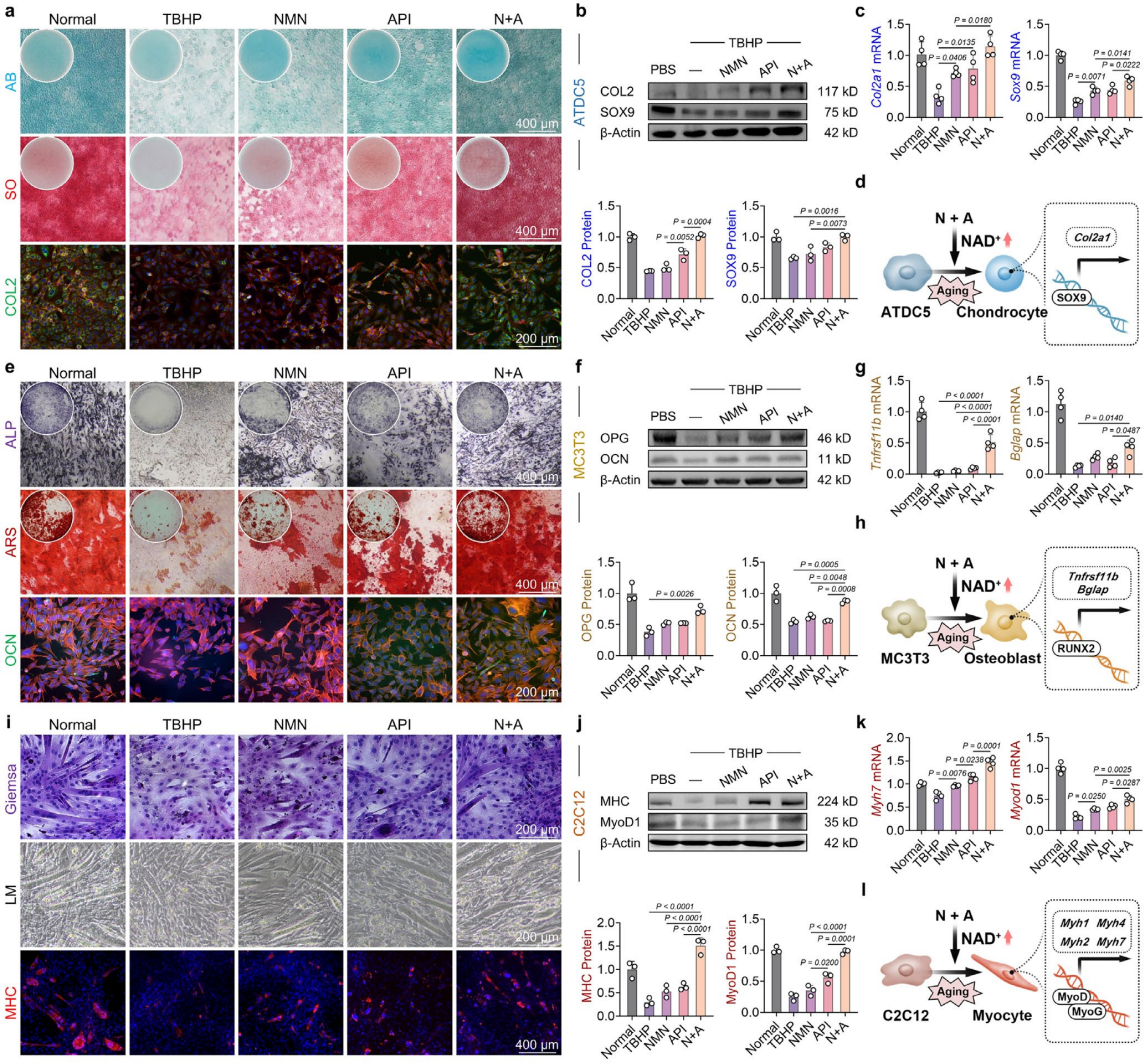
The N + A formulation simultaneously enhanced chondrogenic, osteogenic, and myogenic differentiation in the TBHP‐induced cellular senescence model. (a) Representative images of Alcian blue, safranin O, and COL2 immunofluorescence staining illustrating the effects of intervention on ATDC5 cell differentiation. (b) Western blot analysis of COL2 and SOX9 protein expression in ATDC5 cells following drug intervention (*n* = 3). (c) The mRNA expression levels of *Col2a1* and *Sox9* in ATDC5 cells as determined by quantitative real‐time RT‐PCR (*n* = 4). (d) Schematic diagram illustrating the mechanism by which NAD^+^ supplementation promotes ATDC5 cell differentiation. (e) Representative images of ALP, ARS, and OCN immunofluorescence staining demonstrating the effects of intervention on MC3T3 cell differentiation. (f) Western blot analysis of OCN and OPG protein expression in MC3T3 cells following drug intervention (*n* = 3). (g) The mRNA expression levels of *Tnfrsf11b* and *Bglap* in MC3T3 cells as determined by quantitative real‐time RT‐PCR (*n* = 4). (h) Schematic diagram illustrating the mechanism by which NAD^+^ supplementation promotes MC3T3 cell differentiation. (i) Representative images of Giemsa staining, light microscopy, and MHC immunofluorescence staining showing the effects of intervention on C2C12 cell differentiation. (j) Western blot analysis of MHC and MyoD1 protein expression in C2C12 cells following intervention (*n* = 3). (k) The mRNA expression levels of *Myh7* and *Myod1* in C2C12 cells as determined by quantitative real‐time RT‐PCR (*n* = 4). (l) Schematic diagram illustrating the mechanism by which NAD^+^ supplementation promotes C2C12 cell differentiation. Data are presented as mean ± SD; statistical significance was assessed using one‐way ANOVA, with significant differences between groups indicated by *p* < 0.05.

Given the robust anti‐aging effects of the N + A formulation in musculoskeletal precursor cells, we subsequently investigated its potential to restore tri‐lineage differentiation capacity under aging conditions. In the context of chondrogenic differentiation, N + A treatment enhanced the secretion of cartilage extracellular matrix (C‐ECM) such as glycosaminoglycans (GAGs) and covalently bound proteoglycans (aggrecan) (Figure 3a). Additionally, N + A treatment enhanced cartilage‐enriched type II collagen (COLII) synthesis at both transcriptional and translational levels. Mechanistically, our findings demonstrated that N + A treatment upregulated the transcription factor SOX9, thereby activating the transcriptional activity of the COLII‐encoding gene *Col2a1* (Figure 3b–d and Figure S18). In terms of osteogenic differentiation, N + A treatment promoted the activity of alkaline phosphatase (ALP)—a biomarker of early‐stage osteogenesis—and the deposition of calcium nodules (an indicator of late‐stage osteogenesis) for ossified extracellular matrix (O‐ECM) synthesis (Figure 3e). The marker proteins of osteogenic differentiation, including osteoprotegerin (OPG, encoded by *Tnfrsf11b*) and osteocalcin (OCN, encoded by *Bglap*), were upregulated following treatment with N + A formulation. Additionally, we validated that the transcriptional activation mediated by the transcription factors RUNX family transcription factor 2 (RUNX2) and SP7 regulated the increased osteogenesis (Figure 3f–h and Figure S19). Regarding myogenic differentiation, N + A treatment facilitated both myogenic differentiation and myotube formation, as evidenced by increased myotube width and diameter (Figure 3i). Skeletal muscle is typically classified into two major fiber types: slow‐twitch fibers (which express *Myh7*‐encoded β‐MyHC) and fast‐twitch fibers (which include *Myh2*‐encoded MyHC IIa, *Myh4*‐encoded MyHC IIb, and *Myh1*‐encoded MyHC IIX). These fiber types exhibit distinct contractile properties and metabolic profiles (Motohashi et al. 2019). In N + A‐treated cells, the expression of myogenic differentiation markers, such as the myosin heavy chain (MHC), was upregulated. Notably, treatment with N + A enhanced the expression of myogenic transcription factors, including myogenic differentiation 1 (MyoD1) and myogenin (MYOG), thereby inducing the transcriptional activation of multiple genes within the MyHC superfamily (Figure 3j–l and Figure S20). Taken together, these findings reveal that N + A effectively promoted the tri‐lineage differentiation of musculoskeletal precursor cells.

### 2.4 Oral Administration of N + A Exerted Protective Effects Against Cartilage Damage, Bone Loss, and Muscle Atrophy

**FIGURE 4:**
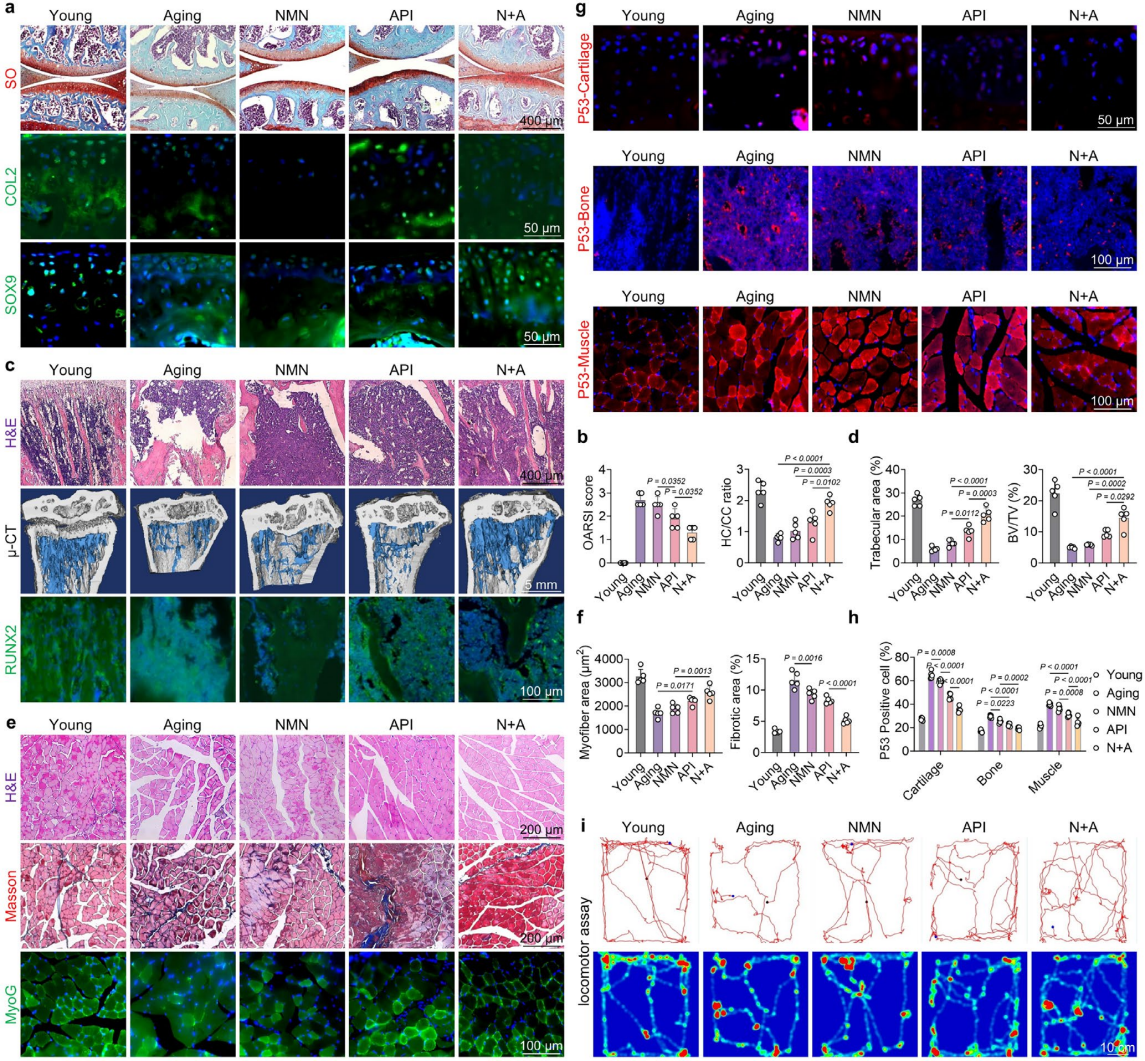
Oral administration of N + A protected against cartilage damage, bone loss, and muscle atrophy in the 20‐month‐old aged mice. (a) Representative images of H&E staining and immunohistofluorescence staining for COL2 and SOX9 in 20‐month‐old aged mouse joints. (b) Quantification of OARSI scores and the hyaline cartilage/calcified cartilage (HC/CC) ratio (*n* = 5). (c) Representative images of H&E staining, micro‐CT (μCT), and immunohistofluorescence staining for RUNX2 in mouse tibias. (d) Quantification of tibial trabecular number (Tb.N) and bone volume fraction (BV/TV, %) in mice based on H&E staining and μCT analysis (*n* = 5). (e) Representative images of H&E staining, Masson's trichrome staining, and immunohistofluorescence staining for MYOG in mouse quadriceps femoris. (f) Quantification of muscle cross‐sectional area and the percentage of fibrosis (*n* = 5). (g) Expression levels of P53 in cartilage, bone, and muscles. (h) Quantitative immunofluorescence analysis of P53 expression (*n* = 5). (i) Representative images from the open field test in mice. Data are presented as mean ± SD; statistical significance was assessed using one‐way ANOVA, with significant differences between groups indicated by *p* < 0.05.

Owing to the excellent anti‐senescent and pro‐differentiation effects observed in the previous in vitro studies, we subsequently evaluated the therapeutic effects of orally administered N + A formulation on musculoskeletal degeneration including cartilage, bone, and muscle, in a naturally aged mouse model. With advancing age, articular cartilage undergoes pronounced surface erosion accompanied by structural disorganization of chondrocytes. Meanwhile, the proportion of SOX9‐ or COLII‐positive chondrocytes was significantly reduced in aging mice. Treatment with N + A reduced the OARSI score, enhanced the hyaline cartilage to calcified cartilage ratio, and reactivated the SOX9‐COLII pathway, demonstrating attenuated degeneration of the cartilage (Figure 4a,b and Figure S21a). Regarding either age‐related OP or senile OP, oral administration of the N + A restored the proportion of trabecular bone area, as demonstrated by H&E staining. Micro‐computed tomography (micro‐CT, μ‐CT) analysis demonstrated that both cancellous bone parameters and cortical bone indices were significantly enhanced following treatment with N + A formulation. Additionally, the expression of the RUNX2‐OCN signaling pathway was upregulated after the treatment with N + A formulation (Figure 4c,d and Figure S21b–e). Furthermore, N + A treatment ameliorated the impaired skeletal muscle morphology, as indicated by increased myofiber area and reduced fibrotic area. The expression levels of various myogenic markers within the skeletal muscle were upregulated in aged mice following treatment with N + A formulation (Figure 4e,f and Figure S21f,g). Furthermore, in vivo immunofluorescence staining revealed that administration of the N + A formulation significantly reduced senescence‐associated biomarkers in cartilage, bone, and muscle tissues. These findings supported the potent and robust anti‐aging effects of N + A formulation on the musculoskeletal system (Figure 4g,h and Figure S22a–c). Ultimately, we comprehensively evaluated the functional parameters of the N + A‐treated aged mice. Following treatment with the N + A formulation, the gross morphology of the muscle was restored, characterized by normalized gait parameters and improved paw grip strength (Figure S23a,b). The open‐field locomotor assay demonstrated that treatment with N + A formulation enhanced motor function by improving multiple aspects of movement vigor including duration, distance, and velocity (Figure 4i and Figure S23c,d). Collectively, these findings reveal that oral administration of N + A prevented musculoskeletal degeneration under aging conditions and promoted functional recovery.

### 2.5 N + A Formulation Mitigated Musculoskeletal Aging via a Mitochondrial SIRT3‐Dependent Mechanism

**FIGURE 5:**
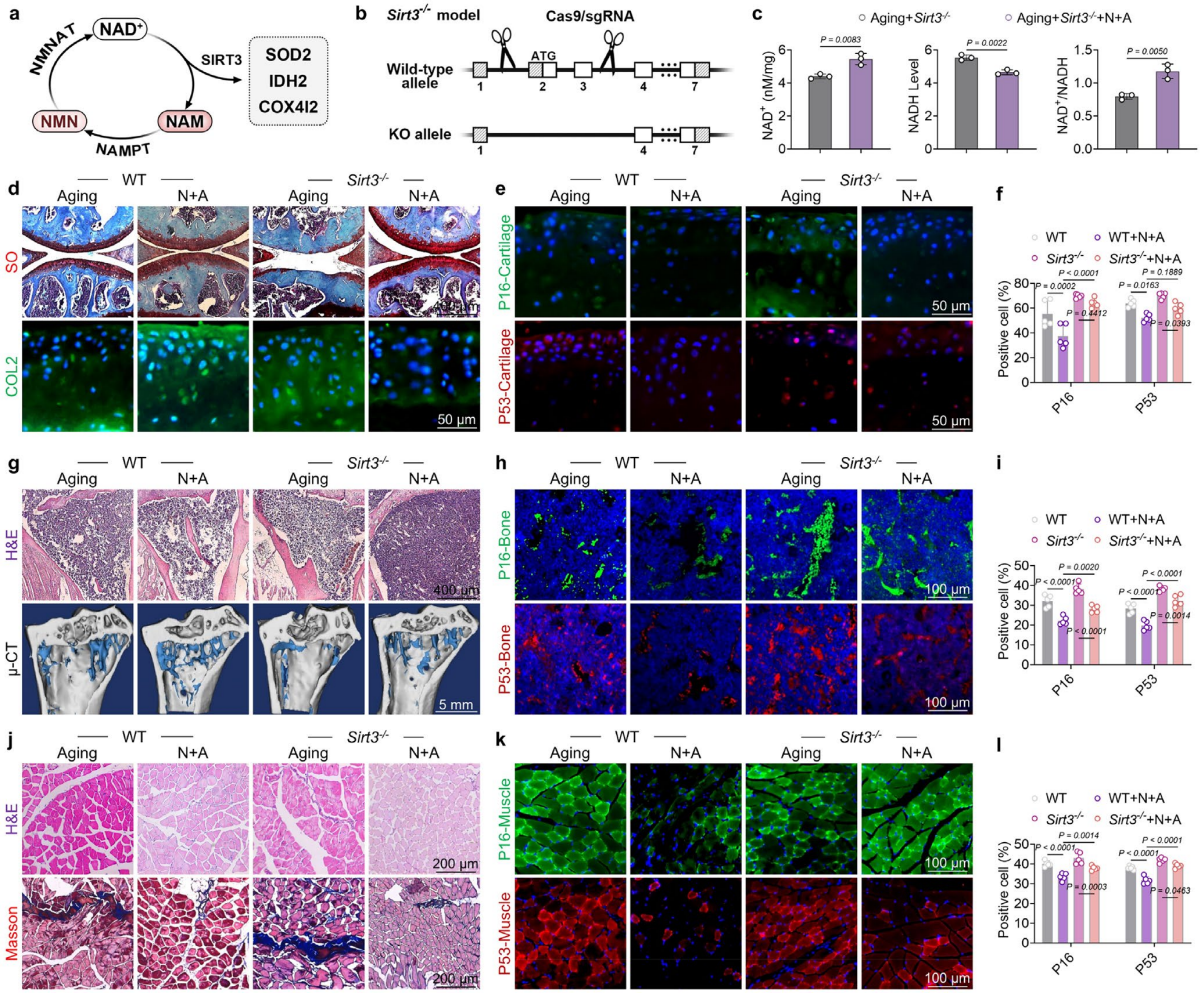
N + A formulation mitigated musculoskeletal aging through a mitochondrial SIRT3‐dependent mechanism. (a) A schematic diagram illustrating the relationship between NAD^+^ metabolism, regulatory pathways, and SIRT3. (b) Technical workflow outlining the SIRT3 gene knockout model. (c) Quantitative analysis of NAD^+^ levels in 20‐month‐old aged SIRT3 knockout mice following NMN and API intervention (*n* = 3). (d) Representative images of Safranin O (S.O.) staining and COL2 immunofluorescence in 20‐month‐old aged SIRT3 knockout mice after NMN and API intervention. (e) Representative images of P16 and P53 immunofluorescence in the cartilage of 20‐month‐old aged SIRT3 knockout mice following NMN and API intervention. (f) Quantitative analysis of P16 and P53 immunofluorescence in mouse cartilage (*n* = 5). (g) Representative images of H&E staining and micro‐CT (μCT) following SIRT3 gene knockout. (h) Representative images of P16 and P53 immunofluorescence in the tibia of 20‐month‐old aged SIRT3 knockout mice after NMN and API intervention. (i) Quantitative analysis of P16 and P53 immunofluorescence in 20‐month‐old aged mouse bone (*n* = 5). (j) Representative images of H&E and Masson's trichrome staining following SIRT3 gene knockout. (k) Representative images of P16 and P53 immunofluorescence in the quadriceps femoris of 20‐month‐old aged SIRT3 knockout mice after NMN and API intervention. (l) Quantitative analysis of P16 and P53 immunofluorescence in 20‐month‐old aged mouse muscle (*n* = 5). Data are presented as mean ± SD; statistical significance was assessed using one‐way ANOVA, with significant differences between groups indicated by *p* < 0.05.

To investigate the mechanisms underlying N + A formulation‐mediated anti‐aging effects in senile musculoskeletal tissues, given the established role of sirtuin 3 (SIRT3) in regulating the NAD^+^ cycling pathway, as well as maintaining mitochondrial redox balance and energy production, we generated a mitochondrial SIRT3 global knockout mouse model (*Sirt3* ^−/−^) using CRISPR‐Cas9 technology (Figure 5a,b). The wild‐type (WT) and SIRT3‐deficient aged mice were orally administered either saline or the N + A formulation. The NAD^+^/NADH quantification assay demonstrated that the N + A formulation significantly elevated both the NAD^+^ levels and the NAD^+^/NADH ratio in the peripheral blood of the SIRT3‐deficient aged mice (Figure 5c). In the context of cartilage protection, SIRT3 knockout diminished the beneficial effects of N + A treatment on articular cartilage integrity and C‐ECM marker expression. Additionally, the senescence‐related proteins P53, P21, and P16 associated with DNA damage were upregulated in the articular cartilage of N + A‐treated mice in the absence of SIRT3 (Figure 5d–f and Figure S24a–c). Regarding bone mass recovery, the impaired clearance of senescent skeletal stem cells caused by SIRT3 deletion in vivo partially attenuated the beneficial effects of N + A on both cortical and cancellous bone parameters (Figure 5g–i and Figure S25a–d). The improvement in the structure of the skeletal muscle induced following treatment with N + A formulation was attenuated in SIRT3‐deficient aged mice, accompanied by exacerbated muscular senescence (Figure 5j–l and Figure S26a–c). In contrast, in aged mice with SIRT3 knockout, the protective effects of N + A formulation on motor function recovery were partially attenuated, as evidenced by declines in both muscle strength and physical activity (Figure S27a–d). Moreover, assays of the acetylation level of mitochondrial proteins demonstrated that N + A decreased the acetylation level of mitochondrial proteins, which may be partially attributed to the enhanced activity of the mitochondrial deacetylase SIRT3 (Figure S28). Taken together, these results show that treatment with N + A formulation delayed musculoskeletal aging and functional impairment, partially through the SIRT3 signaling pathway.

### 2.6 N + A Formulation Regulated the Intestinal Flora to Delay Tissue Senescence and Promote Regenerative Capacity

**FIGURE 6:**
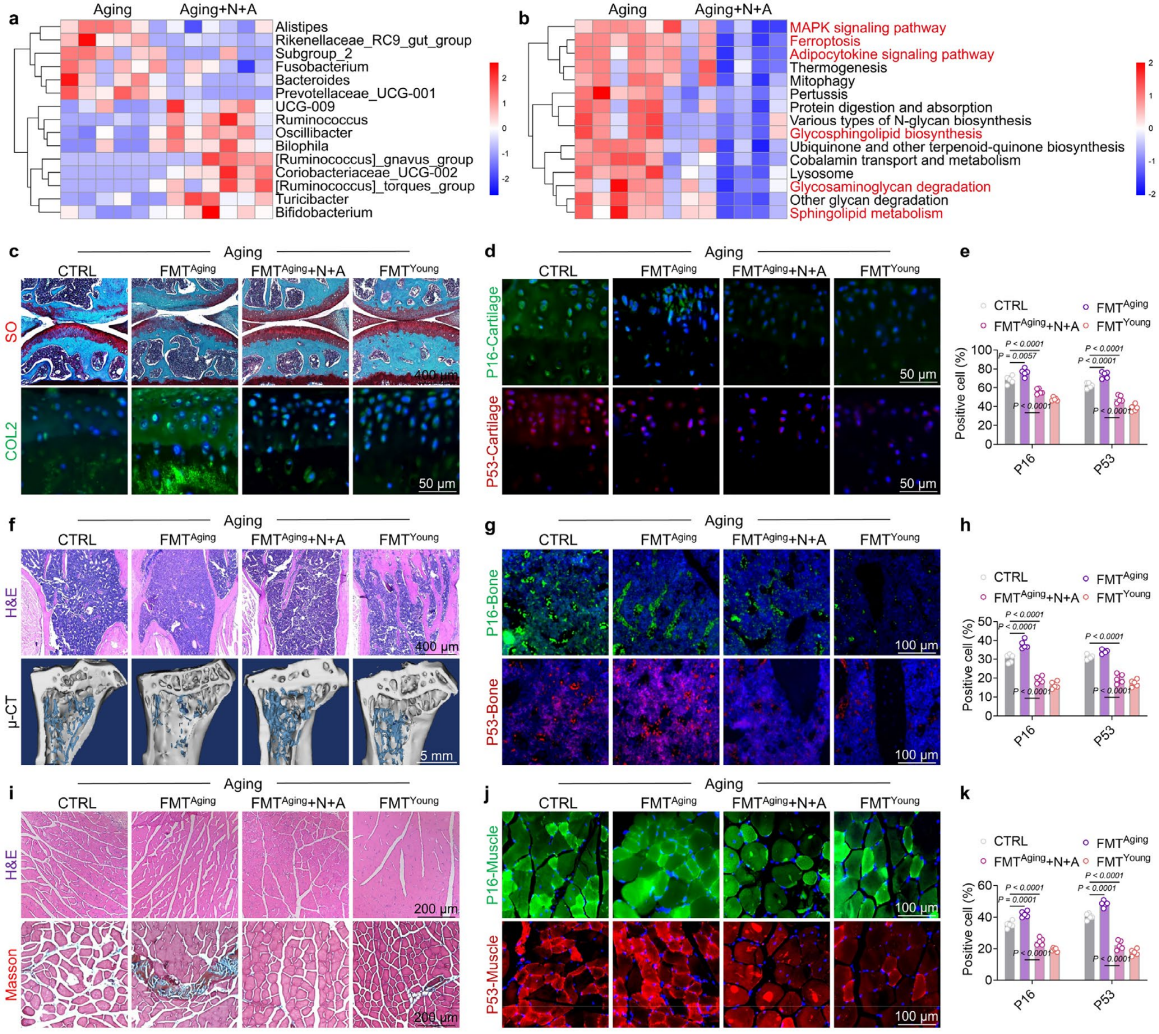
N + A formulation regulated the intestinal flora to delay tissue senescence and promote regenerative capacity. (a, b) Heatmaps representing (a) the differentially expressed gut microbiota; (b) the metabolic/signaling pathways following drug intervention. (c) Representative images of S.O. staining and COL2 immunofluorescence after FMT. (d) Representative images of P16 and P53 immunofluorescence in mouse cartilage following FMT. (e) Quantitative analysis of P16 and P53 immunofluorescence in 20‐month‐old aged mouse cartilage (*n* = 5). (f) Representative images of H&E staining and μCT after FMT. (g) Representative images of P16 and P53 immunofluorescence in 20‐month‐old aged mouse bone following FMT. (h) Quantitative analysis of P16 and P53 immunofluorescence in 20‐month‐old aged mouse bone (*n* = 5). (i) Representative images of H&E and Masson staining after FMT. (j) Representative images of P16 and P53 immunofluorescence in 20‐month‐old aged mouse muscle following FMT. (k) Quantitative analysis of P16 and P53 immunofluorescence in 20‐month‐old aged mouse muscle (*n* = 5). Data are presented as mean ± SD; statistical significance was assessed using one‐way ANOVA, with significant differences between groups indicated by *p* < 0.05.

Recent research establishes that gut microbiota dysbiosis and ecological imbalances are crucial hallmarks of aging, yet they are underexplored mechanisms (López‐Otín et al. 2023). Given the oral route of administration, the intestine is expected to be the primary organ to initially respond to N + A treatment. Therefore, we investigated age‐related pathological changes in both the ileum and colon of aging mice following treatment with the N + A formulation. Histological staining results revealed that N + A treatment increased the number of goblet cells in the intestine, suggesting an enhanced restorative capacity of the gut. Following N + A treatment, the proportion of cells positive for the tight junction protein ZO‐1 (Tjp1) and mucin 2 (Muc2) was significantly increased, indicating a potential restoration of intestinal mucosal barrier integrity (Figure S29). Based on these findings, we subsequently assessed the fecal microbiota composition in aging and N + A‐treated aging mice using 16S rRNA sequencing. The results revealed that N + A treatment significantly enhanced microbial alpha diversity, as evidenced by the increased Shannon, Chao1, and Simpson indices (Figure S30). Additionally, there was a significant difference in microbial beta diversity between the two groups (Figure S31). Subsequently, the composition of the bacterial community was characterized across multiple taxonomic levels including genus, family, order, class, and phylum (Figures S32 and S33). Notably, following treatment with the N + A regimen, at the genus level, the relative abundances of *Alistipes*, *Bacteroides*, *Rikenellaceae_RC9_gut_group*, and *Prevotellaceae_UCG‐001* were decreased, whereas those of *Ruminococcus*, *Oscillibacter*, *Turicibacter*, *Coriobacteriaceae_UCG‐002*, *UCG*‐009, and *[Ruminococcus]_torques_group* were significantly increased (Figure 6a and Figure S34). Kyoto Encyclopedia of Genes and Genomes (KEGG) enrichment analysis revealed that treatment with N + A downregulated the MAPK signaling pathway and ferroptosis; additionally, it inhibited the degradation of GAGs (Figure 6b and Figure S35). Based on these findings, we proposed that the gut microbiota might contribute to the musculoskeletal protective effects mediated by the N + A formulation. To validate this hypothesis, we performed microbiota depletion experiments combined with FMT, whereby gut microbiota were transferred from three donor groups (aged mice, aged mice that had received N + A treatment, and young mice) to recipient aged mice. Compared to the control group, FMT from aged mice exacerbated senescence in cartilage (Figure 6c–e and Figure S36a–c), bone (Figure 6f–h and Figure S37a–d), and muscle tissues (Figure 6i–k and Figure S38a–c), thereby inducing profoundly severe musculoskeletal degeneration. Conversely, FMT from N + A‐treated aged mice reversed senescence and restored regenerative functions among the three tissues, with the effect closely resembling that of FMT from young mice (Figure S39a–d). NMN supplementation ameliorated the disruptions of intestinal homeostasis through the restoration of deoxycholic acid for the prevention of intestinal infections (Fang et al. 2023). Apigenin directly binds to FGR, which contributes to the enhanced phagocytosis of bacteria by macrophages, thereby enhancing the antibacterial ability associated with aging (Gu et al. 2025). Moreover, it is reported that gut microbiota mediates the deamidation of orally administered NMN and its conversion to nicotinic acid (NA) for NAD generation through the Preiss‐Handler pathway (Yaku et al. 2025). Recent views have demonstrated that NAD precursors not only sustain an increase in systemic NAD^+^ levels but also serve as a potent modulator of gut health (Christen et al. 2026). Collectively, these findings reveal that FMT from N + A formulation‐treated mice ameliorated cartilage, bone, and muscle senescent phenotype in aged mice.

### 2.7 Administration of Gut Metabolite PHS Facilitated Musculoskeletal Degeneration and Facilitated Regeneration

**FIGURE 7:**
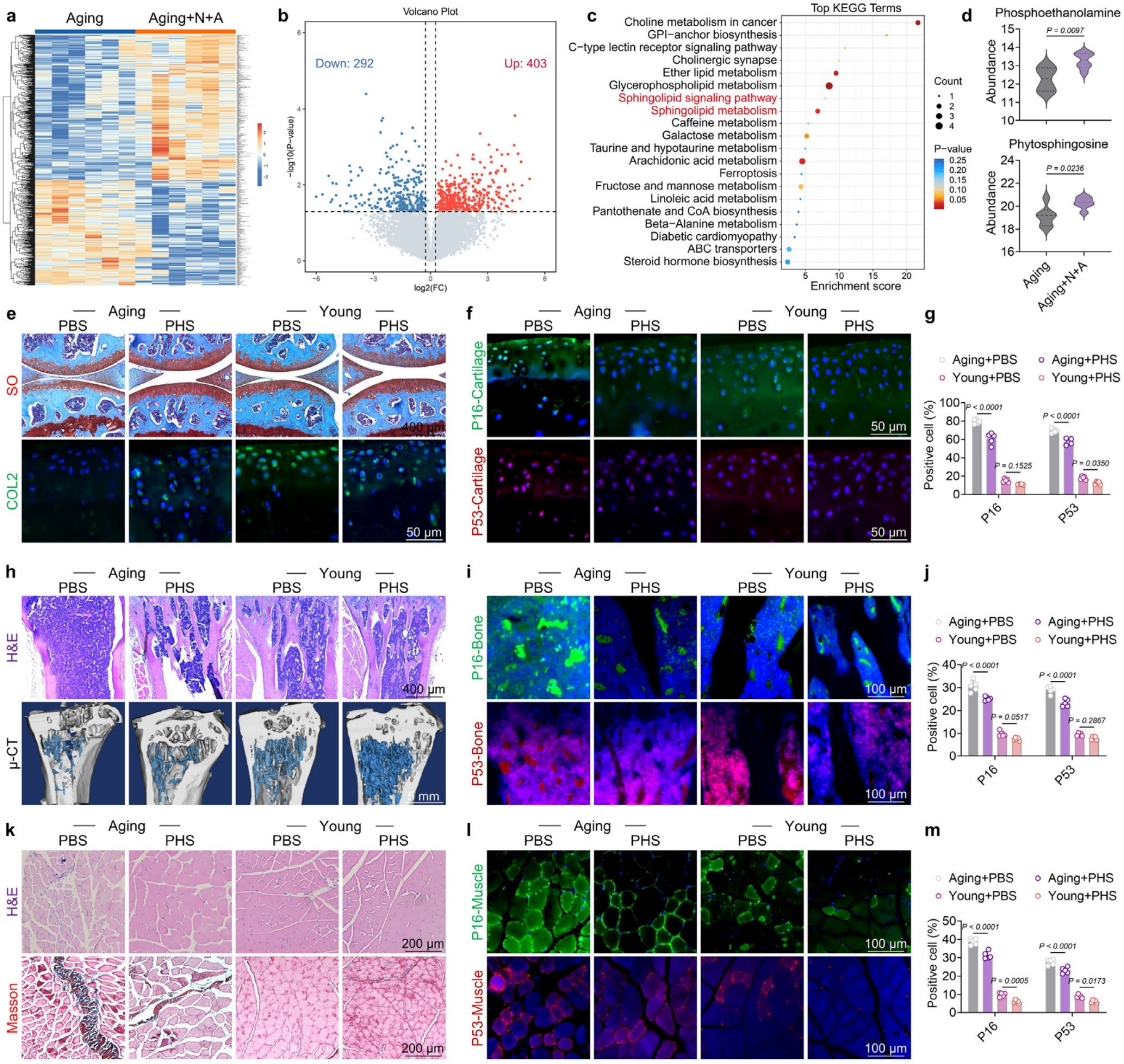
Administration of gut metabolite PHS facilitated musculoskeletal degeneration and facilitated regeneration. (a–d) Multi‐omics differential analysis: (a) Heatmap of differential metabolites; (b) Volcano plot of differentially expressed metabolites; (c) KEGG pathway enrichment analysis of differential metabolites; (d) Box plot depicting the expression levels of key metabolites. (e) Representative images of S.O. staining and COL2 immunofluorescence after PHS treatment. (f) Representative images of P16 and P53 immunofluorescence in young and 20‐month‐old aged mouse cartilage following PHS treatment. (g) Quantitative analysis of P16 and P53 immunofluorescence in young and 20‐month‐old aged mouse cartilage (*n* = 5). (h) Representative images of H&E staining and μCT after PHS treatment. (i) Representative images of P16 and P53 immunofluorescence in mouse bone following PHS treatment. (j) Quantitative analysis of P16 and P53 immunofluorescence in young and 20‐month‐old aged mouse bone (*n* = 5). (k) Representative images of H&E and Masson staining after PHS treatment. (l) Representative images of P16 and P53 immunofluorescence in young and 20‐month‐old aged mouse muscle following PHS treatment. (m) Quantitative analysis of P16 and P53 immunofluorescence in young and 20‐month‐old aged mouse muscle (*n* = 5). Data are presented as mean ± SD; statistical significance was assessed using one‐way ANOVA, with significant differences between groups indicated by *p* < 0.05.

Gut microbiota plays a significant role in the regulation of intestinal homeostasis by producing a wide range of metabolites. These metabolites can penetrate the intestinal mucosa and, in some cases, traverse the intestinal barrier to reach distant organs via the circulatory system (Ezzine et al. 2022). To elucidate key metabolites associated with N + A treatment, untargeted metabolomic profiling was conducted on stool samples collected from both groups. Following administration of the N + A formulation in aged mice, a total of 292 metabolites were downregulated, whereas 403 were upregulated (Figure 7a,b and Figures S40 and S41). The KEGG enrichment analysis revealed that the sphingolipid signaling pathway and sphingolipid metabolism were significantly influenced by the N + A administration. Notably, the sphingolipid metabolism pathway exhibited differential characteristics as revealed by the metabolomic sequencing results (Figure 7c). After screening sphingolipid‐related metabolites from the differential metabolite analysis, two metabolites—phosphoethanolamine (PE) and phytosphingosine (PHS)—were identified as potential candidates. Given the relatively higher abundance and the potential negative effects of PE on senescence (Tighanimine et al. 2024), the PHS was selected as the therapeutic option for degenerative musculoskeletal disorders (Figure 7d). Combined therapeutic strategy of PHS and gamma‐radiation may enhance the sensitivity of cancer cells to radiotherapy (Park et al. 2005). Similarly, supplementation with *Flavonifractor plautii* ‐biosynthesized PHS alleviated metabolic disorders by enhancing PHS levels along the gut–hepatic axis (Li et al. 2025). The co‐analysis of microbes and metabolites demonstrated a strong positive correlation between *Coriobacteriaceae_UCG‐002* (Cintado et al. 2025) and *Ruminococcus* (Vereecke and Elewaut 2017) with PHS (correlation coefficient > 0.5 and *p* value < 0.01), indicating that these intestinal microbiotas may serve as potential contributors to PHS (Figure S42). Ultimately, we evaluated the effects of orally administered PHS on mitigating age‐related musculoskeletal impairments. Treatment with PHS significantly alleviated cartilage degeneration (Figure 7e–g and Figure S43a–c), bone loss (Figure 7h–j and Figure S44a–d), and muscle atrophy (Figure 7k–m and Figure S45a–c), accompanied by the suppression of the P53‐P21 and P16 signaling pathways. Besides architectural improvements, treatment with PHS significantly enhanced muscle strength and mobility performance, especially in aged mice (Figure S46a–c). Taken together, these findings indicate that intestinal metabolite PHS may serve as a promising candidate for the rejuvenation of musculoskeletal degeneration.

## 3 Discussion

The maintenance of intracellular NAD^+^ levels represents a critical regulatory node in cellular and tissue regeneration, and enzymes involved in NAD^+^ biosynthetic pathways constitute promising therapeutic targets. Our results indicated that the NAD^+^ precursor NMN effectively enhances NAD^+^ biosynthesis in musculoskeletal progenitor cells, thereby promoting lineage differentiation into specialized functional cells involved in cartilage, bone, and muscle repair. Besides NMN, other NAD precursors including niacin (also referred to as nicotinic acid, NA or vitamin B3), NR, and NAM, demonstrate promising potential in improving disease phenotypes. Notably, NA participates in the NAMPT‐independent salvage pathway involved in NAD^+^ biosynthesis. Disruption and deficiency of NAD^+^ result in congenital malformations, whereas maternal supplementation with NA during gestation prevents these malformations in mice (Shi et al. 2017). Meanwhile, NAD^+^ repletion therapy with NA has been shown to enhance mitochondrial metabolism, thereby alleviating cancer‐induced cachexia (Beltrà et al. 2023) and mitochondrial myopathy (Pirinen et al. 2020). Another NAD^+^ precursor, NR, is converted into NMN by specific nicotinamide riboside kinases. The NICE randomized clinical trial showed that oral administration of NR enhanced motor function in patients with peripheral artery disease (McDermott et al. 2024). The NADPARK study reported that NR recipients exhibited increased brain NAD^+^ levels, which were closely associated with mild clinical improvement in Parkinson's disease (Brakedal et al. 2022). The novel combination therapy of NR and pterostilbene, beyond the effects of either agent alone, has been shown to effectively attenuate hepatic inflammation in non‐alcoholic fatty liver disease (NAFLD) (Dellinger et al. 2023). Nicotinamide is a vitamin B3 derivative and commonly regarded as a specific inhibitor of the deacetylase enzyme SIRT1. Treatment with NAM promotes mitochondrial metabolism and osteoblast differentiation by activating the SIRT3‐FOXO3A signaling axis (Yoon et al. 2023). The newly identified NAM cell membrane transporters, equilibrative nucleoside transporter 1 and 2 (ENT1 and 2, encoded by SLC29A1 and SLC29A2), improve our understanding of NAD^+^‐related cellular fuel respiration and energy production (Chen et al. 2025). Besides common precursors, several new agents have been identified as potential precursors for NAD^+^ biosynthesis. Nicotinic acid riboside (NaR) is metabolized into NAD^+^ via the action of the cytosolic enzyme NAD^+^ synthase. Oral supplementation of NaR in aged mice has been established to upregulate serum nicotinamide levels and elevate NAD^+^ concentrations across multiple organs (Song et al. 2025). Additionally, the natural alkaloid trigonelline, which is structurally related to NA, enhances muscle function during aging and represents a novel therapeutic option for sarcopenia (Membrez et al. 2024). Notably, the interaction between the NAD metabolism and gut microbiota is attracting more and more attention. NMN supplementation ameliorated the disruptions of intestinal homeostasis through the restoration of deoxycholic acid for the prevention of intestinal infections (Fang et al. 2023). Apigenin directly binds to FGR, which contributes to the enhanced phagocytosis of bacteria by macrophages, thereby enhancing the antibacterial ability associated with aging (Gu et al. 2025). Moreover, it is reported that gut microbiota mediates the deamidation of orally administered NMN and its conversion to nicotinic acid (NA) for NAD generation through the Preiss‐Handler pathway (Yaku et al. 2025). Recent views have demonstrated that NAD precursors not only sustain an increase in systemic NAD^+^ levels but also serve as a potent modulator of gut health (Christen et al. 2026).

To sustain cellular NAD^+^ levels following precursor‐driven enhancement of NAD^+^ biosynthesis, the CD38 inhibitor API was incorporated to limit NAD^+^ consumption. Our results revealed that CD38 inhibition achieves the dominant synergistic effect in increasing NAD^+^ levels and counteracting musculoskeletal senescence. Similarly, the administration of API demonstrated geroprotective effects via the suppression of the SASP, thereby enhancing the efficacy of chemotherapy (Zhang et al. 2025). Additionally, various CD38 enzymatic inhibitors, such as MK‐0159 (Chen et al. 2022) or compound 78c (Tarragó et al. 2018), reverse tissue NAD^+^ depletion, thereby enhancing mitochondrial fitness and improving age‐associated metabolic function. Treatment with the novel anti‐CD38 monoclonal antibody, CM313, alleviates immune thrombocytopenia by mediating the targeted depletion of CD38‐positive cells (Chen et al. 2024). Besides CD38 inhibition, NAD^+^ can be degraded through the activity of NAD^+^‐dependent enzymes such as sirtuins and PARP. Sirtuins catalyze NAD^+^‐dependent lysine deacetylation, coupling the hydrolysis of NAD^+^ to the generation of nicotinamide (NAM), adenosine diphosphate ribose (ADPR), and deacetylated substrate proteins. Our findings indicate that the N + A formulation exerts anti‐aging effects, partially via the mitochondrial SIRT3 signaling pathway. In addition to SIRT3, in the context of musculoskeletal regeneration, liver‐specific deficiency of SIRT2 suppresses osteoclastogenesis and mitigates OP by modulating LRG1 transport (Lin et al. 2023). SIRT6 directly interacts with and deacetylates STAT5, thereby resulting in the attenuation of chondrocyte senescence (Collins et al. 2023; Ji et al. 2022). Targeting SIRT1 therapy through its upstream E3 ubiquitin ligase TRIM16 (Guo et al. 2024) or the downstream mitochondrial biogenesis regulator PGC1α (Shin et al. 2025) can alleviate age‐related muscle dysfunction. In addition to sirtuin‐mediated deacetylation, poly(ADP‐ribose) polymerases (PARPs) constitute another major NAD^+^‐consuming pathway, catalyzing post‐translational ADP‐ribosylation—either mono‐(MARylation) or poly‐ADP‐ribosylation (PARylation)—to regulate DNA damage repair (Chappidi et al. 2024). Inhibition of PARP using either talazoparib (Kyula‐Currie et al. 2025) or olaparib (Abraham et al. 2025) has been increasingly recognized as a promising therapeutic combination strategy along with chemotherapeutic agents for the treatment of tumors. NAD^+^ repletion has been shown to enhance mitochondrial function and reduce PARylation modification, thereby improving muscle function in muscular dystrophy (Ryu et al. 2016). Furthermore, Tankyrase, also known as PARP5a, plays a significant role in the preventing cartilage degeneration by uncoupling SOX9 from a PARylation‐dependent protein degradation pathway (Kim et al. 2019). Additionally, PARP1 suppressed NFATc1 expression and osteoclast formation by promoting PARylation of histone H2B, thereby reducing its occupancy at the NFATc1 promoter, leading to mitigating or prevention of OP (Wang et al. 2020).

## 4 Conclusion

Given the NAD^+^ deficiency observed in aged musculoskeletal tissues, we propose a rational combinatorial regimen employing NMN and API to enhance NAD^+^ biosynthesis while simultaneously inhibiting its degradation, thereby effectively preserving the intracellular NAD^+^ pool. Various animal models have shown that oral administration of the N + A regimen alleviates cartilage, bone, and muscle degeneration in a SIRT3‐dependent manner, with its therapeutic effect being further modulated by the gut microbiota‐derived metabolite PHS. Thus, this study presents a viable strategy for maintaining the NAD^+^ pool, with potential clinical translational value for enhancing musculoskeletal regeneration.

## Author Contributions

J.Y., M.H., and Y.Z. designed the experiments; J.Y., M.H., Y.D., C.Y., and X.X. conducted the experiments; Y.Z., X.L., H.Y., and D.J. were responsible for preparing the original draft of the writing; W.X., Y.Z., and X.Z. contributed to the review and editing of the writing; Y.L. and K.K. were in charge of the visualization. All authors have read and approved the article.

## Funding

This study was supported by National Nature Science Foundation of China (82272494, 82472452, 82402864, 82502126), National Key R&D Program of China (2022YFC2502902), Key Project of Jiangsu Health Commission (K2023079), Natural Science Foundation of Jiangsu Province (BK20240368, BK20250353), Basic Research Pilot Project Suzhou (SSD2024062, SSD2025026), China Postdoctoral Science Foundation (2024M762313, 2025T180666), Suzhou Applied Basic Research (Medical and Health) (SYW2024076), and the Priority Academic Program Development of Jiangsu Higher Education Institutions (PAPD).

## Conflicts of Interest

The authors declare no conflicts of interest.

## Supporting information


**Figure S1:** acel70468‐sup‐0001‐Supinfo.docx.
**Figure S2:** acel70468‐sup‐0001‐Supinfo.docx.
**Figure S3:** acel70468‐sup‐0001‐Supinfo.docx.
**Figure S4:** acel70468‐sup‐0001‐Supinfo.docx.
**Figure S5:** acel70468‐sup‐0001‐Supinfo.docx.
**Figure S6:** acel70468‐sup‐0001‐Supinfo.docx.
**Figure S7:** acel70468‐sup‐0001‐Supinfo.docx.
**Figure S8:** acel70468‐sup‐0001‐Supinfo.docx.
**Figure S9:** acel70468‐sup‐0001‐Supinfo.docx.
**Figure S10:** acel70468‐sup‐0001‐Supinfo.docx.
**Figure S11:** acel70468‐sup‐0001‐Supinfo.docx.
**Figure S12:** acel70468‐sup‐0001‐Supinfo.docx.
**Figure S13:** acel70468‐sup‐0001‐Supinfo.docx.
**Figure S14:** acel70468‐sup‐0001‐Supinfo.docx.
**Figure S15:** acel70468‐sup‐0001‐Supinfo.docx.
**Figure S16:** acel70468‐sup‐0001‐Supinfo.docx.
**Figure S17:** acel70468‐sup‐0001‐Supinfo.docx.
**Figure S18:** acel70468‐sup‐0001‐Supinfo.docx.
**Figure S19:** acel70468‐sup‐0001‐Supinfo.docx.
**Figure S20:** acel70468‐sup‐0001‐Supinfo.docx.
**Figure S21:** acel70468‐sup‐0001‐Supinfo.docx.
**Figure S22:** acel70468‐sup‐0001‐Supinfo.docx.
**Figure S23:** acel70468‐sup‐0001‐Supinfo.docx.
**Figure S24:** acel70468‐sup‐0001‐Supinfo.docx.
**Figure S25:** acel70468‐sup‐0001‐Supinfo.docx.
**Figure S26:** acel70468‐sup‐0001‐Supinfo.docx.
**Figure S27:** acel70468‐sup‐0001‐Supinfo.docx.
**Figure S28:** acel70468‐sup‐0001‐Supinfo.docx.
**Figure S29:** acel70468‐sup‐0001‐Supinfo.docx.
**Figure S30:** acel70468‐sup‐0001‐Supinfo.docx.
**Figure S31:** acel70468‐sup‐0001‐Supinfo.docx.
**Figure S32:** acel70468‐sup‐0001‐Supinfo.docx.
**Figure S33:** acel70468‐sup‐0001‐Supinfo.docx.
**Figure S34:** acel70468‐sup‐0001‐Supinfo.docx.
**Figure S35:** acel70468‐sup‐0001‐Supinfo.docx.
**Figure S36:** acel70468‐sup‐0001‐Supinfo.docx.
**Figure S37:** acel70468‐sup‐0001‐Supinfo.docx.
**Figure S38:** acel70468‐sup‐0001‐Supinfo.docx.
**Figure S39:** acel70468‐sup‐0001‐Supinfo.docx.
**Figure S40:** acel70468‐sup‐0001‐Supinfo.docx.
**Figure S41:** acel70468‐sup‐0001‐Supinfo.docx.
**Figure S42:** acel70468‐sup‐0001‐Supinfo.docx.
**Figure S43:** acel70468‐sup‐0001‐Supinfo.docx.
**Figure S44:** acel70468‐sup‐0001‐Supinfo.docx.
**Figure S45:** acel70468‐sup‐0001‐Supinfo.docx.
**Figure S46:** acel70468‐sup‐0001‐Supinfo.docx.
**Table S1:** acel70468‐sup‐0001‐Supinfo.docx.

## Data Availability

The data that support the findings of this study are available on request from the corresponding author. The data are not publicly available due to privacy or ethical restrictions.
