# Supplementary material for: Double‐Pronged NAD Preservation: Delaying Cellular Senescence and Initiating Musculoskeletal Regeneration
Source: Aging Cell. 2026 Apr 7;25(4):e70468. doi: 10.1111/acel.70468 (PMC13054677; doi:10.1111/acel.70468)
Supplement: Supplementary file 1 — Figure S1: acel70468‐sup‐0001‐Supinfo.docx. Figure S2: acel70468‐sup‐0001‐Supinfo.docx. Figure S3: acel70468‐sup‐0001‐Supinfo.docx. Figure S4: acel70468‐sup‐0001‐Supinfo.docx. Figure S5: acel70468‐sup‐0001‐Supinfo.docx. Figure S6: acel70468‐sup‐0001‐Supinfo.docx. Figure S7: acel70468‐sup‐0001‐Supinfo.docx. Figure S8: acel70468‐sup‐0001‐Supinfo.docx. Figure S9: acel70468‐sup‐0001‐Supinfo.docx. Figure S10: acel70468‐sup‐0001‐Supinfo.docx. Figure S11: acel70468‐sup‐0001‐Supinfo.docx. Figure S12: acel70468‐sup‐0001‐Supinfo.docx. Figure S13: acel70468‐sup‐0001‐Supinfo.docx. Figure S14: acel70468‐sup‐0001‐Supinfo.docx. Figure S15: acel70468‐sup‐0001‐Supinfo.docx. Figure S16: acel70468‐sup‐0001‐Supinfo.docx. Figure S17: acel70468‐sup‐0001‐Supinfo.docx. Figure S18: acel70468‐sup‐0001‐Supinfo.docx. Figure S19: acel70468‐sup‐0001‐Supinfo.docx. Figure S20: acel70468‐sup‐0001‐Supinfo.docx. Figure S21: acel70468‐sup‐0001‐Supinfo.docx. Figure S22: acel70468‐sup‐0001‐Supinfo.docx. Figure S23: acel70468‐sup‐0001‐Supinfo.docx. Figure S24: acel70468‐sup‐0001‐Supinfo.docx. Figure S25: acel70468‐sup‐0001‐Supinfo.docx. Figure S26: acel70468‐sup‐0001‐Supinfo.docx. Figure S27: acel70468‐sup‐0001‐Supinfo.docx. Figure S28: acel70468‐sup‐0001‐Supinfo.docx. Figure S29: acel70468‐sup‐0001‐Supinfo.docx. Figure S30: acel70468‐sup‐0001‐Supinfo.docx. Figure S31: acel70468‐sup‐0001‐Supinfo.docx. Figure S32: acel70468‐sup‐0001‐Supinfo.docx. Figure S33: acel70468‐sup‐0001‐Supinfo.docx. Figure S34: acel70468‐sup‐0001‐Supinfo.docx. Figure S35: acel70468‐sup‐0001‐Supinfo.docx. Figure S36: acel70468‐sup‐0001‐Supinfo.docx. Figure S37: acel70468‐sup‐0001‐Supinfo.docx. Figure S38: acel70468‐sup‐0001‐Supinfo.docx. Figure S39: acel70468‐sup‐0001‐Supinfo.docx. Figure S40: acel70468‐sup‐0001‐Supinfo.docx. Figure S41: acel70468‐sup‐0001‐Supinfo.docx. Figure S42: acel70468‐sup‐0001‐Supinfo.docx. Figure S43: acel70468‐sup‐0001‐Supinfo.docx. Figure S44: acel70468‐sup‐0001‐Supinfo.docx. Figure S45: acel70468‐sup‐000 [file ACEL-25-e70468-s001.docx]

**Supplementary Materials and Methods**

**Animal Models**

The experimental protocol received approval from the Ethics Committee of Soochow University (approval number: SUDA20250320A02) and adhered strictly to the guidelines set forth by the National Institutes of Health. Mice were sourced from the Experimental Animal Center of Soochow University and housed under controlled conditions at 25°C with a 12-hour light/dark cycle. They were provided with sterile food and water. Following a one-month acclimatization period, the mice were used for experimental procedures. All animal experiments were approved by the Laboratory Animal Committee. During the study, mouse body weights were recorded daily. Upon completion of the experiment, the mice were euthanized, weighed, and biological samples were collected for subsequent analysis.

**Gene Knockout Models**

Global Sirt3 knockout (*Sirt3*^−/−^) mice used in the gene knockout model were derived according to our previous study(Zhang et al., 2023) and maintained until 20 months of age to generate aged *Sirt3*^−/−^ mice. Genetic characterization was performed on tail-derived DNA following protocols from The Jackson Laboratory. Mice were housed in a controlled environment with constant temperature, a 12-hour light/dark cycle, and consistent humidity, while being fed a standard diet.

**Microcomputed Tomography (μCT) Analysis**

After euthanasia, knee joints were fixed and subjected to high-resolution microcomputed tomography (μCT) using the Skyscan 1176 system (Bruker, Kontich, Belgium), with a resolution of 9 μm, and instrument settings of 50 kV and 200 μA. Scans were reconstructed using NRecon v1.6 (Bruker), and three-dimensional models were generated with Dataviewer v1.5.2.4 (Bruker), CTAn v1.13.8.1 software (Bruker), and Mimics Research v19.0 software (Materialise, Belgium). Relevant specimen parameters were extracted from the target areas using CTAn software. The following indicators were measured: bone mineral density (BMD), bone volume fraction (BV/TV), trabecular number (Tb.N), trabecular thickness (Tb.Th), trabecular separation (Tb.Sp), cortical thickness (Ct.Th), and cortical bone area fraction (Ct.Ar/Tt.Ar)(Fukuda et al., 2018).

**Cell Culture**

ATDC5 and C2C12 cells were cultured in high-glucose DMEM medium (Keygen Bio ECH) supplemented with 10% fetal bovine serum (FBS, Vazyme, Nanjing, China) and 1% penicillin-streptomycin solution (Thermo Fisher Scientific, Waltham, MA, USA). MC3T3 cells were cultured in α-MEM medium (Procell, Wuhan, China) containing 10% fetal bovine serum and 1% penicillin-streptomycin solution. All cells were incubated in a temperature-controlled environment at 37°C with 5% CO_2_.

**Myogenic Differentiation of C2C12**

Once C2C12 cells reached 70%–80% confluence in high-glucose DMEM (Keygen Bio ECH) medium supplemented with 10% FBS, the original medium was discarded, and the cells were washed twice with PBS. The medium was then replaced with differentiation induction medium, consisting of high-glucose DMEM with 1% penicillin-streptomycin (Thermo Fisher Scientific) and 2% horse serum (Biosharp, Guangzhou, China). Fresh medium was replenished every two days during differentiation. Upon the formation of myotubes, the staining procedure was initiated. Cells were fixed with 4% paraformaldehyde for 15 minutes, followed by three PBS washes to remove residual fixative. Giemsa staining solution (Beyotime) was applied at room temperature for 10–15 minutes. After staining, cells were gently rinsed three times with distilled water to remove excess stain, leaving a clear background. Imaging was performed using a camera mounted on an upright microscope, with myotube fibers, rich in proteins, appearing as dark purple structures. The total number of nuclei in each image was manually counted, and the fusion index was calculated as the percentage of nuclei within myotubes relative to the total number of nuclei(Tan et al., 2023).

**Osteogenic Differentiation of MC3T3**

When cell confluence reached 80%–90%, adherent MC3T3 cells were digested with 0.25% trypsin/EDTA (Thermo Fisher Scientific), subcultured until 90% confluence, or seeded in 24-well plates at a density of 2 × 10^5^ cells/well to induce osteogenic differentiation(Ambrosi et al., 2025). Osteoblast differentiation medium (α-MEM) supplemented with 10% FBS, 10 mM β-glycerophosphate (Sigma-Aldrich), 50 μg/mL ascorbic acid (Sigma-Aldrich), and 1% penicillin-streptomycin solution was used(Zhong et al., 2024). Differentiation was induced for 7–14 days, with medium changes every two days. After seven days, alkaline phosphatase (ALP) activity was analyzed using an ALP staining kit (Beyotime, Shanghai, China). After 14 days, mineralized nodule formation was assessed by ARS staining (Solarbio, Beijing, China), followed by extraction of ARS with 10% cetylpyridinium chloride to quantify mineralization. Absorbance was measured at 595 nm(Kim et al., 2023).

**Chondrogenic Differentiation of ATDC5**

ATDC5 cells were cultured in DMEM/F-12 medium (Keygen Bio ECH) supplemented with 10% FBS and 1% penicillin-streptomycin (Gibco). Upon reaching confluence, the medium was replaced with DMEM/F-12 containing 5% FBS, 1% insulin-transferrin-selenium (Absin), 1% penicillin-streptomycin, 10 mM β-glycerophosphate, and 50 μg/mL L-ascorbic acid-2-phosphate (Sigma-Aldrich)(Marchan-Alvarez et al., 2024). The cells were maintained in a temperature-controlled incubator, with medium changes every two days. When confluence reached 80%-90%, cells were digested with 0.25% trypsin/EDTA (Thermo Fisher Scientific), passaged to 90% confluence, and seeded into 24-well plates at a density of 2 × 10^5^ cells/well. Following differentiation, the medium was removed, and cells were washed with PBS before fixation with 4% paraformaldehyde. Pre-treatment with alcian acidification solution (Solarbio) was applied for 3 minutes, followed by staining with alcian blue for 30 minutes. After rinsing with distilled water for 1 minute, cells were observed and photographed using an optical microscope (Carl Zeiss, Oberkochen, Germany).

**Quantitative Reverse Transcription Polymerase Chain Reaction (RT-PCR)**

For RNA extraction, cells were cultured in 6-well plates and treated accordingly. Total RNA was extracted using TRIzol® reagent (Thermo Fisher Scientific), and RNA concentration and purity were assessed using a NanoDrop ND-2000 spectrophotometer (Thermo Fisher Scientific). cDNA synthesis was performed using a cDNA synthesis kit (Vazyme), utilizing the extracted RNA as a template. Quantitative reverse transcription PCR (RT-PCR) was conducted using a real-time fluorescence quantitative PCR machine, SYBR qPCR premix (Vazyme), and primer sequences listed in Supplementary Table 1. The comparative Ct method (2^−ΔΔCt^) was used for quantification.

**Western Blotting**

Protein samples were collected using RIPA lysis buffer (NCM Biotech, Suzhou, China). After centrifugation at 12,000g for 15 minutes at 4°C, the supernatant was isolated. Protein concentration was determined using a BCA protein quantification kit (Vazyme). The protein was mixed with loading buffer and boiled at 100°C for 5 minutes. Equal protein amounts were loaded onto SDS-PAGE gels (Vazyme) for electrophoresis. Following electrophoresis, proteins were transferred to nitrocellulose membranes. After blocking for 30 minutes at room temperature with blocking solution (Beyotime), membranes were incubated overnight at 4°C with primary antibodies diluted as recommended. Membranes were then incubated with horseradish peroxidase (HRP)-conjugated secondary antibodies (goat anti-rabbit IgG (H+L) or goat anti-mouse IgG (H+L), 1:10,000, Affinity Biosciences) for 1 hour at room temperature. After washing, membranes were treated with ultra-sensitive enhanced chemiluminescence (ECL) reagent (NCM). Protein bands were captured using a chemiluminescence imaging system (SH-523, Shenhua Technology, Hangzhou, China), and band intensity was quantified using Image J software (National Institutes of Health, Bethesda, MD, USA).

**Mitochondrial Membrane Potential Detection**

Following the intervention, the culture plate was removed, the supernatant discarded, and the cells gently washed three times with pre-warmed PBS solution at 37°C. To ensure even coverage of the staining solution, serum-free medium containing 5 μmol/L JC-1 fluorescent probe (Sigma-Aldrich) was added to each well. The plate was then incubated at 37°C with 5% CO_2_ for 20 minutes in the dark. After incubation, the cells were washed twice with PBS to remove any unbound probe, and the stained solution was discarded. Fluorescence imaging was performed immediately using a Zeiss Axiovert 40CFL upright fluorescence microscope. Fluorescence images were captured in the same field of view under both the red channel (590 nm) and the green channel (525 nm). The mitochondrial membrane potential (ΔΨm) was objectively quantified by calculating the ratio of red fluorescence intensity at 590 nm to green fluorescence intensity at 525 nm.

**NAD and NADH level measurement**

The NADH content in cells was quantified using the Coenzyme I NAD(H) content detection kit (Elabscience, Wuhan, China). Typically, 10% homogenates of fresh muscle, cartilage, and bone tissues were prepared in lysis buffer through repeated freeze-thaw cycles to ensure complete homogenization. Alternatively, 1.5×10⁶ cells were resuspended in lysis buffer for lysis. After centrifugation at 12,000 g for 10 minutes at 4°C, the supernatant was transferred to a new tube for protein content measurement. A microplate reader was used to measure absorbance at 450 nm after adding 20 μL of the samples and 120 μL of the reaction working solution to each well. The plate was incubated for 30 minutes at 37°C. Under the influence of an electron-coupling reagent, NADH converted WST-8 into an orange-yellow product with a maximum absorption peak at approximately 450 nm. After sample extraction, the solution was heated at 60°C for 30 minutes to break down NAD⁺ and preserve NADH. The NAD⁺/NADH ratio was calculated by first determining the total amounts of NAD⁺ and NADH from the OD values, subtracting the NADH amount from the total to obtain the NAD⁺ content.

**ATP Level**

Intracellular ATP levels were assessed using the ATP Detection Kit (Beyotime). After cell lysis, the lysate was transferred to a 1.5 mL centrifuge tube and centrifuged at 12,000 g for 5 minutes at 4°C. The supernatant was combined with the ATP detection working solution in a white 96-well plate, mixed gently, and incubated in a chemiluminescence microplate reader. After a 5-minute reaction at room temperature in the dark, the relative luminescence intensity of each well was measured. ATP concentration was normalized to total protein content.

**Assay of CD38 Enzymatic Activity**

CD38 activity was measured in strict accordance with the manufacturer's protocol (JONLNBIO, China). Briefly, the cells were trypsinized, resuspended in PBS supplemented with protease inhibitors, and then underwent three freeze-thaw cycles in liquid nitrogen, followed by homogenization. The mixture was centrifuged at 12,000×g for 10 minutes at 4°C. Immediately after centrifugation, the supernatant was harvested and assayed, and the CD38 activity was calculated using the standard curve provided by the kit.

**Measurement of Oxygen Consumption Rate**

The oxygen consumption rate (OCR) was measured using an XFe24 analyzer (Seahorse Bioscience, North Billerica, Massachusetts, USA). Briefly, cells were seeded into XF24 microplates at a density of 2×10^5^ cells/well and pre-incubated overnight. Before the assay, the cells were equilibrated in unbuffered XF assay medium for 1 hour. The XFe Cell Mito Stress Test Kit was used according to the manufacturer's instructions, with automated injection of inhibitors (2 μM oligomycin, a complex V inhibitor; 1 μM FCCP, an uncoupler; and 1 μM antimycin A and rotenone, complexes III and I inhibitors). The OCR was detected sequentially, and core mitochondrial respiration parameters were calculated from the OCR curves.

**Experimental grouping and treatment**

In the first experiment, wild-type (WT) mice were randomly divided into five groups based on age and intervention: the young control group (2-month-old), the aged control group (20-month-old), the NMN group (20-month-old; NMN was orally administered at a dose of 300 mg/kg/day, prepared as a 30 mg/mL solution in sterile normal saline from Macklin), the API group (20-month-old; API was orally administered at a dose of 100 mg/kg/day, prepared as a 10 mg/mL suspension with 0.5% carboxymethyl cellulose sodium (CMC-Na) as the solvent from Macklin), and the N+A combination group (20-month-old; NMN and API were co-administered orally at the aforementioned doses). All 20-month-old groups received the respective interventions via oral gavage for two months.

In the second experiment, 20-month-old WT and *Sirt3^⁻/⁻^* mice were randomly assigned to two groups according to their genotype: an aging control group, which was administered sterile water, and an N+A treatment group, which received a combination of NMN and apigenin. All mice underwent the respective treatments for two months under the aforementioned regular housing conditions.

In the third experiment, 20-month-old WT mice were selected as transplant recipients. The fecal microbiota donors consisted of three cohorts of WT mice: 2-month-old young mice, 20-month-old mice, and 20-month-old mice pre-treated with N+A for 2 months. The recipients were randomly and evenly divided into four experimental groups: the blank control group (CTRL), the group receiving fecal microbiota transplantation from aged donors (FMT Aging), the group receiving FMT from N+A-pre-treated donors (FMT N+A), and the group receiving FMT from young donors (FMT Young). All recipient groups, excluding the CTRL group, underwent antibiotic-mediated gut microbiota depletion to clear the intestinal flora prior to the FMT intervention. After successful gut microbiota depletion, the mice in the three FMT groups were administered fresh donor fecal slurry by gavage every 3 days for a total of 2 months. The CTRL group was given an equal volume of sterile PBS by gavage following the same schedule as the FMT groups.

In the fourth experiment, 2-month-old and 20-month-old WT mice were independently randomized into two groups for each age group: the control group, which received oral gavage of PBS, and the treatment group, which received oral gavage of phytosphingosine (PHS; 25 mg/kg/day, Macklin). All mice underwent continuous daily gavage for a total of two months. Mice were administered 25 mg/kg of phytosphingosine by oral gavage daily for 2 months(Montenegro-Burke et al., 2021).

**Skeletal Muscle Histomorphometry**

At the conclusion of the experimental protocol, mice were euthanized, and the quadriceps femoris, along with the surrounding muscle tissue, was excised. These tissues were rinsed with saline solution and fixed in 4% paraformaldehyde for more than 48 hours. After fixation, the muscle tissue was weighed, then processed by embedding, dehydration, and immersion fixation before being sectioned into 5-μm paraffin slices. Hematoxylin-eosin (H&E) staining and immunofluorescent labeling of muscle markers were performed on the dewaxed skeletal muscle sections. The cross-sectional area (CSA) of muscle fibers in the H&E-stained sections was quantified using Image J software.

**Bone Histomorphometry**

Following euthanasia, mouse knee joint and tibia samples were fixed in 4% paraformaldehyde and decalcified using 10% ethylenediaminetetraacetic acid (EDTA, Sigma-Aldrich, USA). The samples were then dehydrated through a graded ethanol series, embedded in paraffin, and sectioned into 6-μm slices. The sections were stained with H&E (Jiancheng, Nanjing, China) and safranin O-fast green (S.O.) (Sigma-Aldrich) for histological analysis. Quantitative assessments of tibial trabecular structure were performed. The ratio of hyaline cartilage (HC) to calcified cartilage (CC) was calculated, and cartilage degeneration was evaluated according to the criteria established by the International Osteoarthritis Research Society (OARSI).

**Immunofluorescent Staining**

Immunofluorescent Labeling of Treated Cells: Cells were cultured on 24-well plates with coverslips. Following treatment, the medium was removed, and the cells were washed with PBS (Procell). They were then fixed with 4% paraformaldehyde for 20 minutes and permeabilized with 0.3% Triton X-100 (Beyotime) for 15 minutes, followed by washing. Next, the cells were blocked using QuickBlock™ blocking buffer (Beyotime) for 30 minutes, prior to overnight incubation with the target primary antibody at a dilution of 1:300 at 4°C. The following day, the primary antibody was removed, and the cells were incubated with either Fluor488-conjugated or CY3-conjugated goat anti-rabbit IgG (H+L) secondary antibodies (1:500, Affinity Biosciences) for 1 hour. The cells were then stained with FITC phalloidin or TRITC phalloidin (1:200, Solarbio) for 30 minutes and counterstained with DAPI for 1 minute. All procedures were carried out in darkness. Fluorescence images were captured using a fluorescence microscope, and the fluorescence intensity was quantified using Image J software (National Institutes of Health).

Immunofluorescent Labeling of Tissue Samples: Tissue sections were deparaffinized, and endogenous peroxidase activity was inhibited by immersion in 3% hydrogen peroxide (Sigma-Aldrich) for 10 minutes. Antigen retrieval was performed by treating the sections with 0.125% trypsin (Thermo Fisher Scientific) at 37°C for 30 minutes. The sections were then incubated with various primary antibodies at a dilution of 1:200 at 4°C overnight. The following day, sections were incubated with Fluor488 or CY3-conjugated goat anti-rabbit IgG (H+L) secondary antibody for 1 hour, followed by counterstaining with DAPI in the dark. Fluorescent signals were visualized and documented using a fluorescence microscope, and positively stained cells were quantified using Image J software.

**Antibiotic Treatment**

The antibiotic solution was prepared by dissolving ampicillin (0.25 mg/mL), metronidazole (0.25 mg/mL), neomycin (0.25 mg/mL), and vancomycin (0.125 mg/mL) (MACKLIN, Shanghai, China) in autoclaved water in appropriate proportions(Yu et al., 2025). The solution was then sterilized by filtration through a 0.22 μm sterile filter, dispensed into sterile reagent bottles, and stored in the dark at 4°C. It was intended for use within 72 hours of preparation. Seven days prior to the experimental intervention, mice designated for gut microbiota depletion were administered the antibiotic solution. To avoid the lingering effects of the antibiotics on subsequent experiments, the mice were switched to regular autoclaved water immediately after the seven-day administration period.

**Fecal Microbiota Transplantation (FMT)**

One week before transplantation, recipient mice were treated with antibiotics to deplete their native gut microbiota. Fecal samples from donor mice were collected from aged mice that had received oral treatment for 2 months (experimental group), as well as from age-matched untreated mice and younger mice. Fresh fecal pellets were obtained using sterile forceps, and precisely 100 mg of feces was placed into a centrifuge tube containing 1 mL of sterile PBS for homogenization. The mixture was centrifuged at 300 g for 30 seconds, and the supernatant was collected as the bacterial solution, which was used immediately for transplantation(Tang et al., 2019). The entire transplantation procedure was completed within 30 minutes. During transplantation, 100 μL of the bacterial solution was gradually administered to the recipient mice *via* a gavage needle. Mice in the experimental group received fecal bacterial solutions derived from aged, untreated, and young mice.

**Intestinal histopathology**

For histological analysis, the intestinal tissues (small intestine and colon) of the mice were excised, washed with PBS to remove luminal contents, and fixed in 4% paraformaldehyde for 48 hours before being paraffin-embedded and sectioned. Small intestinal sections underwent Alcian Blue-Periodic Acid-Schiff (AB-PAS) staining using a kit from Biosharp to highlight the structural features of the small intestine and colon, with particular focus on goblet cells. Representative regions were selected for quantitative assessment of goblet cell populations. Sections of the small intestine and colon were also stained with an H&E staining kit. Immunofluorescence staining of intestinal tissues was performed using Muc2 (ABclonal Technology, China) and Tjp1 (ABclonal Technology, China) antibodies; nuclei were counterstained with DAPI. Fluorescence intensity was measured and recorded using a fluorescence microscope (Zeiss).

**Gait Analysis**

Gait analysis was systematically conducted on mice from all experimental groups during the second month of the study. The forepaws of the mice were marked, and the hind paws were marked with green marking fluid. A pristine white recording paper was placed on a flat surface in the laboratory, and the mice, with their paws marked, were individually positioned at the starting point of a dark enclosure. This enclosure was designed as a closed rectangular tube with a quiet, dimly lit interior. The starting gate of the enclosure was opened, allowing the mice to move freely from one end to the other without external interference. During the procedure, experimenters only observed and recorded the data, without any intervention. Each mouse underwent the procedure three times, with a five-minute interval between trials. After the experiment, three consecutive footprints were selected from the recording paper for statistical analysis.

**Grip Strength Test**

The YLIDA-DS2 digital dynamometer was used to measure the grip strength of the mice. Before experimentation, the dynamometer's metal grid was adjusted to an appropriate height, aligned with the natural extension of the mice's forepaws, and calibrated to ensure an accurate zero point. The posterior part of each mouse's trunk was gently held, and once the forepaws securely grasped the metal grid, the device was gradually and steadily pulled backward in a horizontal direction. Throughout the procedure, the dynamometer's real-time readings were closely monitored. The maximum force exerted by the mouse was recorded at the moment it released the grid, indicating it had reached its muscular strength limit, representing the individual muscle grip strength. Each mouse underwent two trials, with a 15-minute interval between tests. The average of these two measurements was calculated to represent the muscle grip strength of each individual mouse.

**Skeletal Muscle Contraction Force Test**

Prior to euthanasia, the mice were anesthetized, and the quadriceps femoris muscle along with the femoral nerve was meticulously isolated from each experimental group. The femoral nerve was positioned vertically between two parallel electrodes, while the tendon was secured and connected to a force sensor (Zhongshi Technology, Beijing, China). The muscle tissues were maintained in a hydrated state using Ringer's solution at 37°C. Tetanic muscle contractions were induced through electrical stimulation (250 Hz, 25 V, 1 second)(Wang et al., 2023). To prevent muscle fatigue from compromising the stability of contractile force, a minimum interval of 5 minutes was observed between each stimulation. Each muscle was subjected to three repeated measurements on both sides. The tension curves of muscle contraction were recorded synchronously using a data acquisition system (Zhongshi Technology). The absolute contractile force, defined as the difference between the maximum contractile force and the baseline tension, was calculated and subsequently normalized to muscle mass. After the experiment, the connective tissues on the surface of the quadriceps femoris muscles were carefully removed, and the muscles were blotted dry using filter paper before being weighed. Normalizing the absolute contractile force by muscle mass provided the specific contractile force, which helped mitigate the influence of muscle size variability on the assessment of contractile function. All procedures were completed within 30 minutes following anesthesia to minimize the impact of anesthesia depth fluctuations on neuromuscular excitability.

**Open-field Test**

This test assessed the general locomotor activity level of the mice. The mice were placed in a quiet, thermostatic, and clean closed open-field platform (45 cm×45 cm×40 cm), allowing them to walk freely, while their movement was recorded with a camera for 3 minutes. TM-vision behavioral monitoring software was used to record total distance traveled, movement speed, and duration of activity. Mice were carefully placed in the central region of the open-field platform, starting from the geometric center. After each trial, the platform was thoroughly cleaned with 75% ethanol and allowed to dry completely before the next trial.

**Cell Counting Kit-8 (CCK-8) Assay**

Cell viability was assessed using the Cell Counting Kit-8 (CCK-8, NCM Biotech). Cells were cultured until optimal confluence, then gently digested with a 0.25% trypsin-EDTA solution. After digestion, cell counts were determined using a hemocytometer, and approximately 5,000 to 10,000 cells were seeded into each well of a 96-well plate. The intervention medium was added to facilitate experimental treatment. Following the intervention, 10 μL of CCK-8 reagent was introduced into each well, and the reaction was incubated at 37°C for one hour. Absorbance was measured at 450 nm using a microplate reader.

**Live/dead staining**

The effects of N + A stimulation on the proliferation of ATDC5, MC3T3, and C2C12 cells were determined using a Calcein/PI Cell Viability Assay Kit. The experimental protocol was as follows: The three aforementioned cell lines were seeded into 12-well plates at a density of 2×10⁵ cells per well. After 5 days of co-incubation with 400 μM NMN and 10 μM API, the cells were rinsed twice with PBS. Subsequently, according to the manufacturer's instructions, 500 μL of the prepared Calcein/PI live-dead staining working solution was added to each well, and the cells were incubated at 37°C in the dark for 30 min. After staining, the cells were rinsed again with PBS, and finally, cell viability was observed and analyzed under a microscope.

**Molecular docking**

The CD38 protein structure was retrieved from the Uniprot database (PDB ID: P56528), and the structure of the API was obtained from PubChem (CID: 5280443). To analyze the potential binding sites between API and CD38, AutoDock Vina (version 1.1.2) was employed. Proteins were designated as receptors, and small molecules were designated as ligands. PyMOL software (version 4.3.0, https://pymol.org/) was used to separate the original ligand from the protein structure, remove water and organic matter. AutodockTools (http://mgltools.scripps.edu/downloads) was used for hydrogenation, to check and calculate the charge, specify the atomic type as AD4 type, and construct the docking grid box of the protein structure. Finally, in AutodockTools, the formats of protein structures and small molecule ligands were converted to "PDBQT". After docking with Vina, the scores of pairwise combinations of proteins and small molecules were calculated, and force analysis and visualization from three-dimensional and two-dimensional angles were performed using Pymol and Discovery Studio software.

**Mitochondrial protein extraction**

N+A-intervened cells were detached with 0.25% trypsin-EDTA. After collecting 2×10⁷ cells and centrifuging, the pellet was resuspended in 1 mL ice-cold mitochondrial extraction buffer (Reagent A) and homogenized on ice until 50–60% cell disruption was achieved. The homogenate was transferred to a 2 mL tube, mixed with 1 mL ice-cold Reagent B, and centrifuged at 600g, 4°C, 10 min. The supernatant was collected and centrifuged again at 10,000g, 4°C, 10 min. The resulting pellet was washed once with ice-cold mitochondrial wash solution, then resuspended in 0.5 mL ice-cold wash solution containing 425 μL Reagent C and 75 μL Reagent D. After vortexing, it was centrifuged at 21,000g, 4°C, 10 min; the supernatant was discarded. The pellet was washed again with ice-cold wash solution (16,000g, 4°C, 5 min), and the final mitochondrial pellet was resuspended in 200 μL ice-cold preservation solution. For integrity assessment, 5 μL of this suspension was mixed with 5 μL Janus Green B and examined by light microscopy. The remaining suspension was centrifuged (16,000g, 4°C, 5 min), the supernatant discarded, and the pellet lysed in 100 μL mitochondrial lysis buffer+protease inhibitor cocktail. Lysates were gently mixed to extract total mitochondrial proteins for Western blot analysis of acetylated mitochondrial proteins.

**Single-cell analysis**

Data analysis was performed on myofibroblasts (MF), muscle stem cells (MuSC), chondrocytes (CH), and osteoblasts (OB) obtained from the GEO database. The Seurat package was used to assess the quality of the single-cell expression matrix. After quality control, the data were normalized using the Seurat package's NormalizeData function. The FindAllMarkers function in Seurat was employed to identify specific marker genes for each cell cluster. The expression distribution of the four cell marker genes was visualized using VlnPlot, and the spatial localization of the four cell types was annotated on the UMAP plot using FeaturePlot. The osteoblast and chondrocyte data were sourced from GSE145477. Sequencing data for skeletal muscle can be accessed at https://www.muscleageingcellatlas.org/.

**Metabolomics analysis**

Prior to euthanasia, fecal samples from mice were collected, rapidly frozen in liquid nitrogen, and stored for subsequent analysis. The samples were thawed in 1.5 mL centrifuge tubes (EP tubes), to which 300 μL of a protein precipitant solution (methanol-water, V:V = 4:1, containing mixed internal standards at a concentration of 4 μg/mL) was added(Nossa et al., 2010). After homogenization, 150 μL of the supernatant was transferred to a liquid chromatography-mass spectrometry (LC-MS) vial and stored at -80°C until LC-MS analysis. Metabolomic data analysis was conducted by Shanghai OE Biotech Co., Ltd. Metabolic profiling was performed using an ACQUITY UPLC I-Class plus system (Waters Corporation, Milford, USA), coupled with a Q-Exactive mass spectrometer and a heated electrospray ionization (ESI) source (Thermo Fisher Scientific) in both positive and negative ion modes. Compound identification and subsequent metabolic pathway analysis were conducted using reference databases, including the Human Metabolome Database (HMDB, http://www.hmdb.ca/), Lipidmaps Database (http://www.lipidmaps.org/), Metlin Database (https://ngdc.cncb.ac.cn/), LuMet-Animal3.0 self-built database, and KEGG Database(Gao et al., 2023).

**16S rDNA gene sequencing analysis of Gut Microbiota.**

Fecal samples from the intervention group of mice were collected before euthanasia, placed into sterile cryopreservation tubes, and transported to a refrigerator maintained at -80°C for preservation. The samples were then sent to Shanghai OE Biotech Co., Ltd. (Shanghai, China) for sequencing. Amplicon quality was assessed using agarose gel electrophoresis. The PCR products were purified with AMPure XP beads (Agencourt) and subjected to a second round of PCR for amplicon enrichment. After re-purification with AMPure XP beads, the final amplicons were quantified using the Qubit dsDNA Assay Kit (Thermo Fisher Scientific), and the concentrations were adjusted for sequencing. Sequencing was performed on an Illumina NovaSeq 6000 (Illumina Inc., San Diego, CA)(Zheng et al., 2024).

S**tatistical Analysis**

Statistical analysis was performed using SPSS 13.0 software (SPSS Inc., Chicago, Illinois, USA). Data are presented as mean ± standard deviation (SD). A one-way analysis of variance (ANOVA) was used to compare multiple groups, while a two-tailed unpaired Student's *t*-test was employed to compare two groups. Statistical significance was set at *P* < 0.05.

**References**

Ambrosi, T. H., Morales, D., Chen, K., Hunt, E. J., Weldon, K. C., Maifeld, A. N., . . . Lane, N. E. (2025). Basigin links altered skeletal stem cell lineage dynamics with glucocorticoid-induced bone loss and impaired angiogenesis. *Nat Commun, 16*(1), 7606. doi:10.1038/s41467-025-62881-w

Fukuda, M., Yoshizawa, T., Karim, M. F., Sobuz, S. U., Korogi, W., Kobayasi, D., . . . Yamagata, K. (2018). SIRT7 has a critical role in bone formation by regulating lysine acylation of SP7/Osterix. *Nat Commun, 9*(1), 2833. doi:10.1038/s41467-018-05187-4

Gao, J., Wang, L., Jiang, J., Xu, Q., Zeng, N., Lu, B., . . . He, X. (2023). A probiotic bi-functional peptidoglycan hydrolase sheds NOD2 ligands to regulate gut homeostasis in female mice. *Nat Commun, 14*(1), 3338. doi:10.1038/s41467-023-38950-3

Kim, J., Kim, B. Y., Lee, J. S., Jeong, Y. M., Cho, H. J., Park, E., . . . Jeong, S. Y. (2023). UBAP2 plays a role in bone homeostasis through the regulation of osteoblastogenesis and osteoclastogenesis. *Nat Commun, 14*(1), 3668. doi:10.1038/s41467-023-39448-8

Marchan-Alvarez, J. G., Teeuwen, L., Mamand, D. R., Gabrielsson, S., Blomgren, K., Wiklander, O. P. B., & Newton, P. T. (2024). A protocol to differentiate the chondrogenic ATDC5 cell-line for the collection of chondrocyte-derived extracellular vesicles. *J Extracell Biol, 3*(9), e70004. doi:10.1002/jex2.70004

Montenegro-Burke, J. R., Kok, B. P., Guijas, C., Domingo-Almenara, X., Moon, C., Galmozzi, A., . . . Wolan, D. W. (2021). Metabolomics activity screening of T cell-induced colitis reveals anti-inflammatory metabolites. *Sci Signal, 14*(702), eabf6584. doi:10.1126/scisignal.abf6584

Nossa, C. W., Oberdorf, W. E., Yang, L., Aas, J. A., Paster, B. J., Desantis, T. Z., . . . Pei, Z. (2010). Design of 16S rRNA gene primers for 454 pyrosequencing of the human foregut microbiome. *World J Gastroenterol, 16*(33), 4135-4144. doi:10.3748/wjg.v16.i33.4135

Tan, A., Younis, A. Z., Evans, A., Creighton, J. V., Coveny, C., Boocock, D. J., . . . Doig, C. L. (2023). PARP1 mediated PARylation contributes to myogenic progression and glucocorticoid transcriptional response. *Cell Death Discov, 9*(1), 133. doi:10.1038/s41420-023-01420-2

Tang, T. W. H., Chen, H. C., Chen, C. Y., Yen, C. Y. T., Lin, C. J., Prajnamitra, R. P., . . . Hsieh, P. C. H. (2019). Loss of Gut Microbiota Alters Immune System Composition and Cripples Postinfarction Cardiac Repair. *Circulation, 139*(5), 647-659. doi:10.1161/circulationaha.118.035235

Wang, R., Wang, F., Lu, S., Gao, B., Kan, Y., Yuan, T., . . . Si, Y. (2023). Adipose-derived stem cell/FGF19-loaded microfluidic hydrogel microspheres for synergistic restoration of critical ischemic limb. *Bioact Mater, 27*, 394-408. doi:10.1016/j.bioactmat.2023.04.006

Yu, C., Sun, R., Yang, W., Gu, T., Ying, X., Ye, L., . . . Yao, S. (2025). Exercise ameliorates osteopenia in mice via intestinal microbial-mediated bile acid metabolism pathway. *Theranostics, 15*(5), 1741-1759. doi:10.7150/thno.104186

Zhang, Y., Liu, Y., Hou, M., Xia, X., Liu, J., Xu, Y., . . . Zhu, X. (2023). Reprogramming of Mitochondrial Respiratory Chain Complex by Targeting SIRT3-COX4I2 Axis Attenuates Osteoarthritis Progression. *Adv Sci (Weinh)*, e2206144. doi:10.1002/advs.202206144

Zheng, Y., Zhang, Z., Fu, Z., Fan, A., Song, N., Wang, Q., . . . Liu, X. (2024). Oral Propolis Nanoemulsions Modulate Gut Microbiota to Balance Bone Remodeling for Enhanced Osteoporosis Therapy. *ACS Nano*. doi:10.1021/acsnano.4c07332

Zhong, C., Li, N., Wang, S., Li, D., Yang, Z., Du, L., . . . Zhang, G. (2024). Targeting osteoblastic 11β-HSD1 to combat high-fat diet-induced bone loss and obesity. *Nat Commun, 15*(1), 8588. doi:10.1038/s41467-024-52965-4

**Supplementary Figures**

**
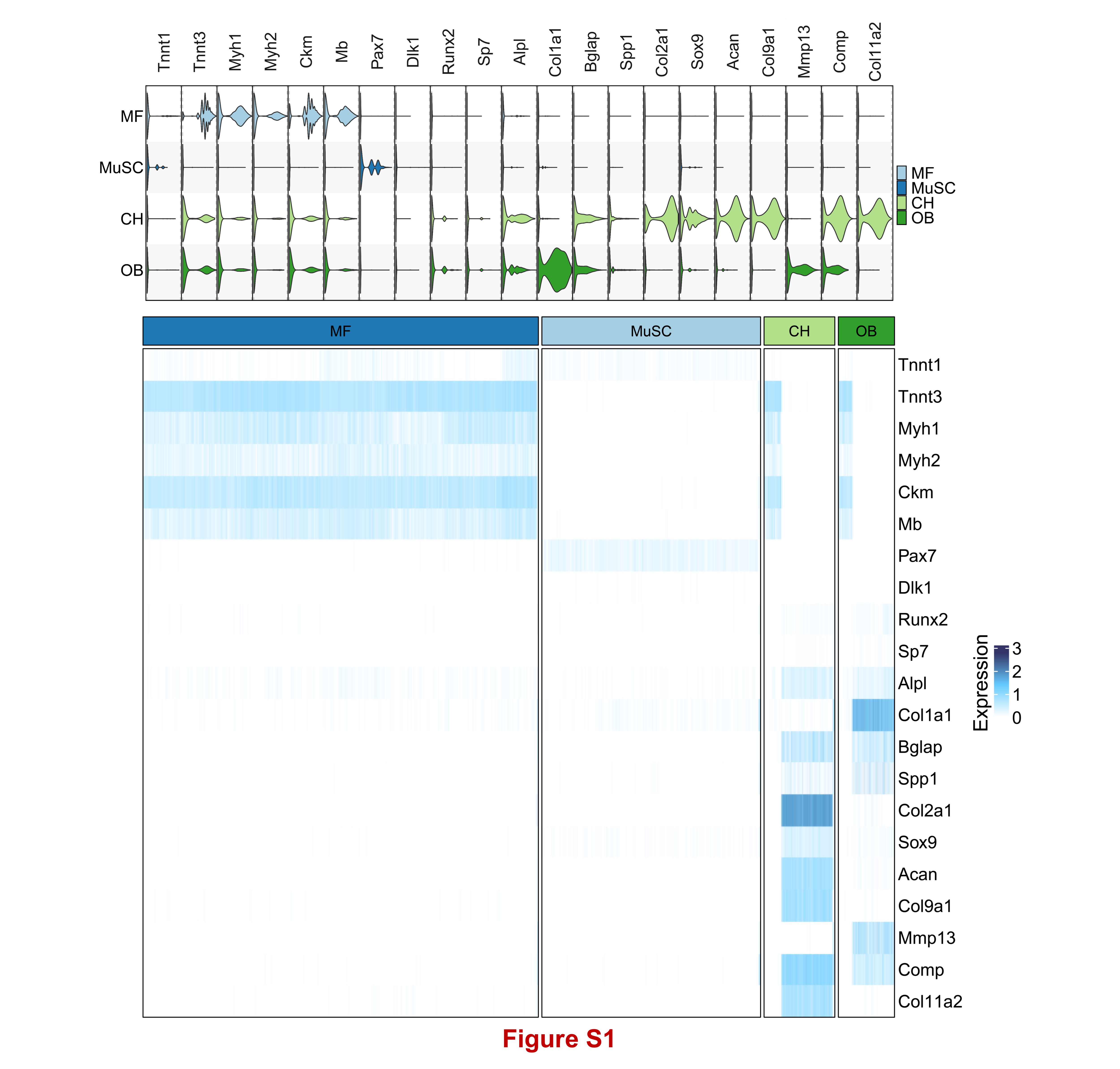
**

**Figure S1.** Single-cell RNA sequencing was performed to analyze the gene expression profiles across different cell types. The plot illustrates the expression patterns of key genes in muscle fibers (MF), muscle stem cells (MuSC), chondrocytes (CH), and osteoblasts (OB).


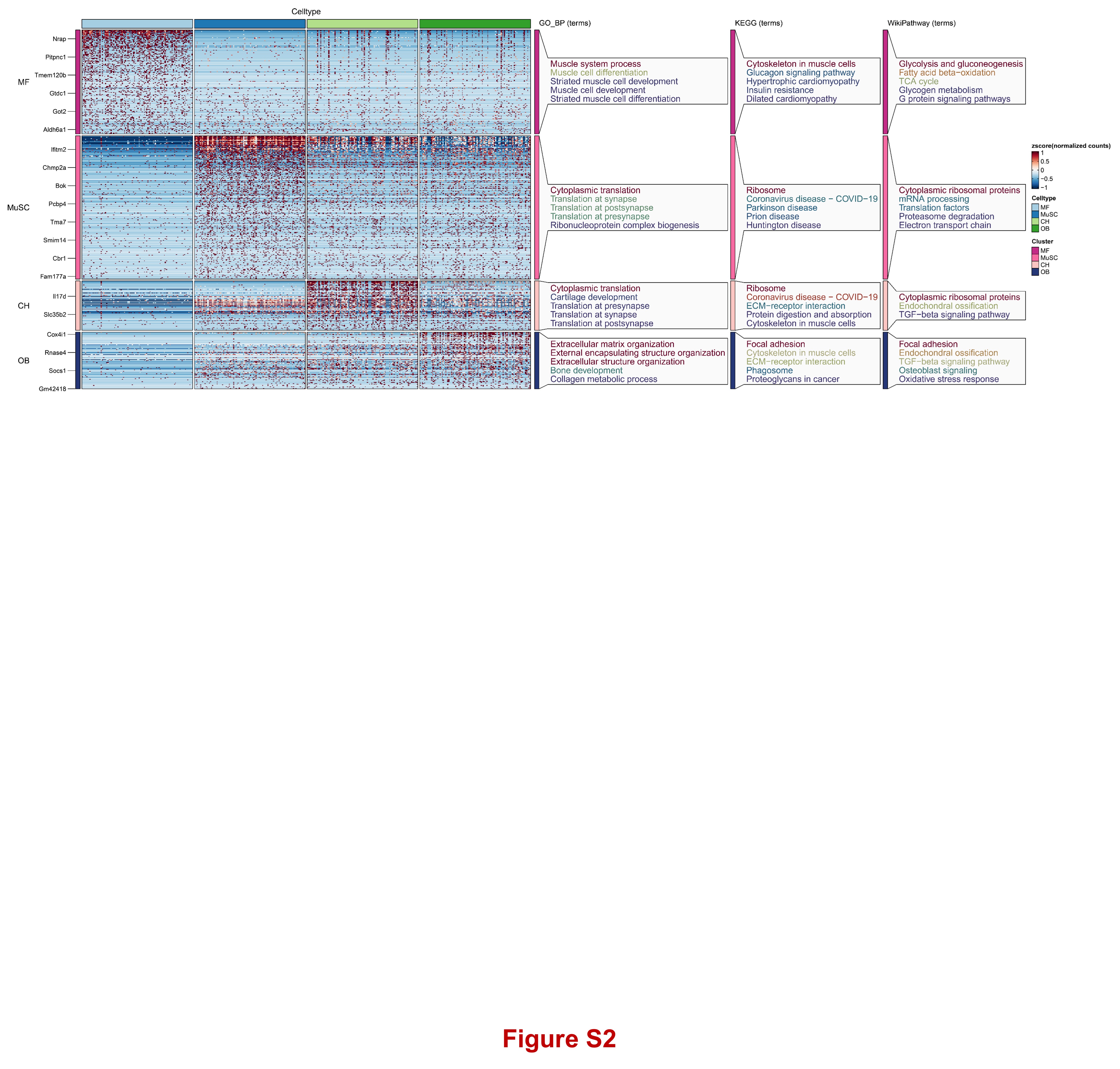


**Figure S2.** Single-cell functional pathway enrichment analysis elucidates the cellular heterogeneity within the musculoskeletal system. The heatmap on the left illustrates the expression patterns of differentially expressed genes across muscle fibers (MF), muscle stem cells (MuSC), chondrocytes (CH), and osteoblasts (OB). On the right, the functional characteristics of each cell type are summarized based on annotations from the GO (Biological Process), KEGG, and WikiPathway databases.


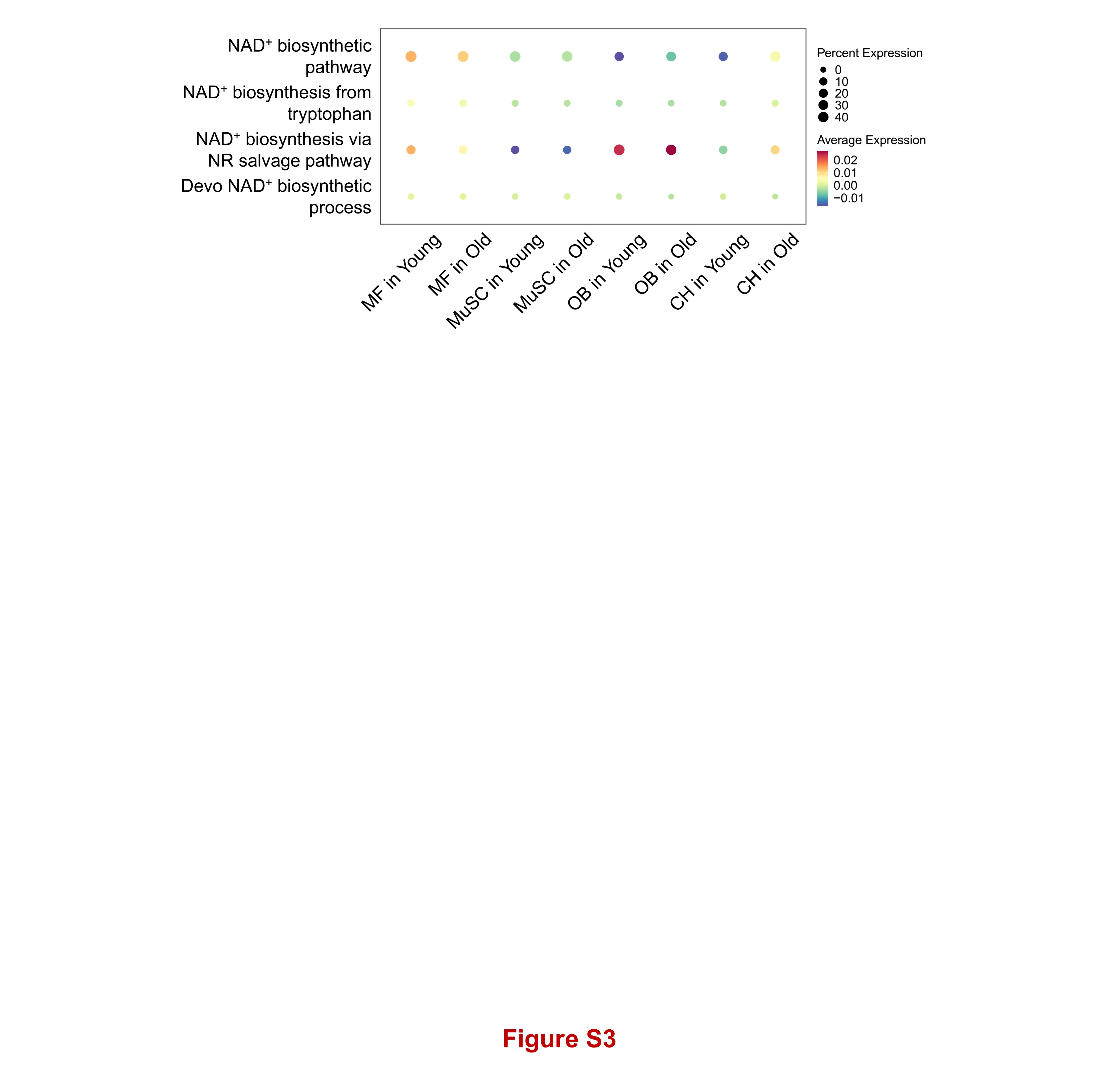


**Figure S3.** Expression profiles of the NAD⁺ biosynthesis pathway across different cell types: muscle fibers (MF), muscle stem cells (MuSC), osteoblasts (OB), and chondrocytes (CH) under both young and old conditions.


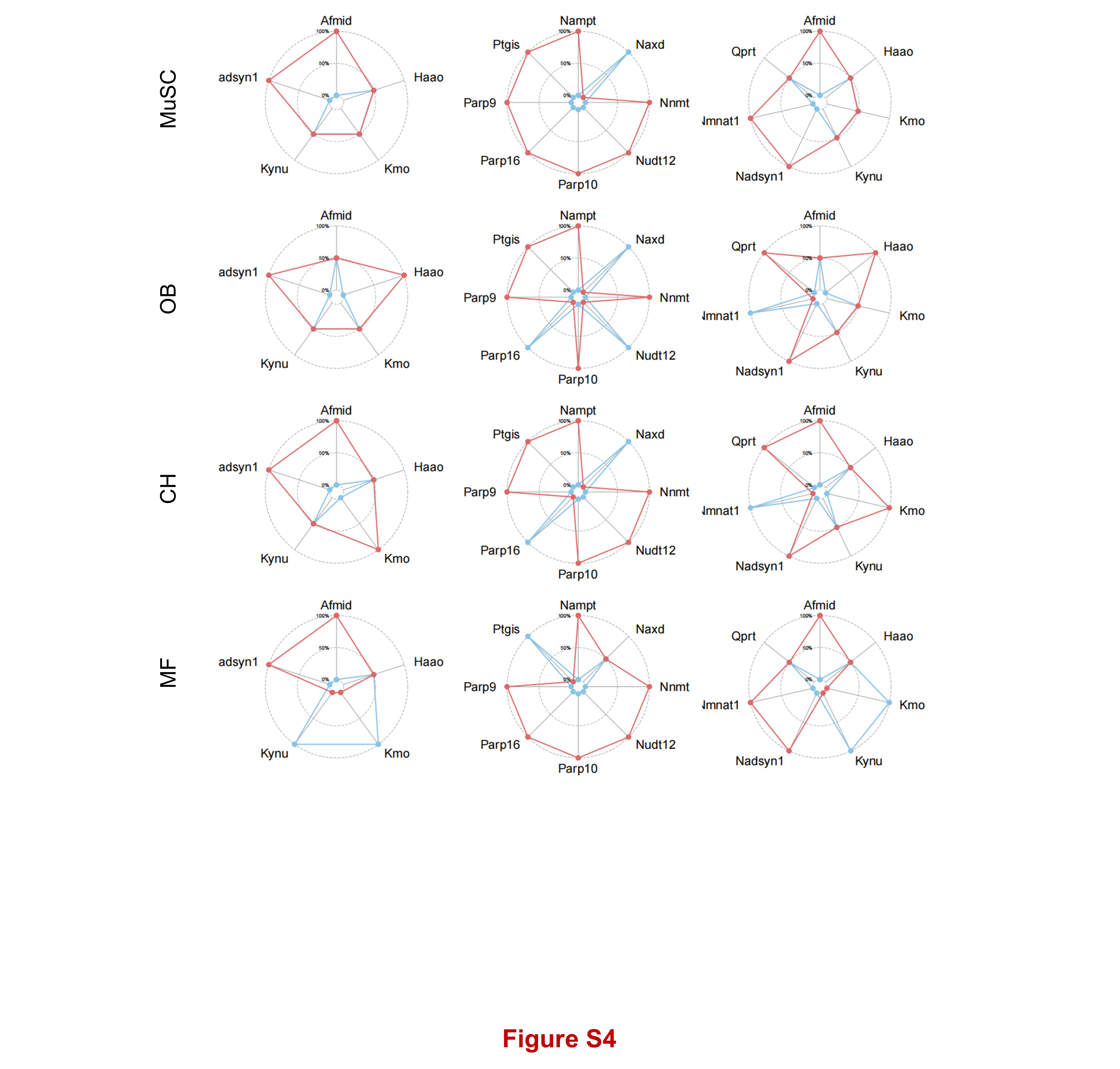


**Figure S4.** Radar chart of the expression characteristics of NAD⁺ metabolism in different cell types: muscle fibers (MF), muscle stem cells (MuSC), osteoblasts (OB), and chondrocytes (CH) under both young (red line) and old conditions (blue line).


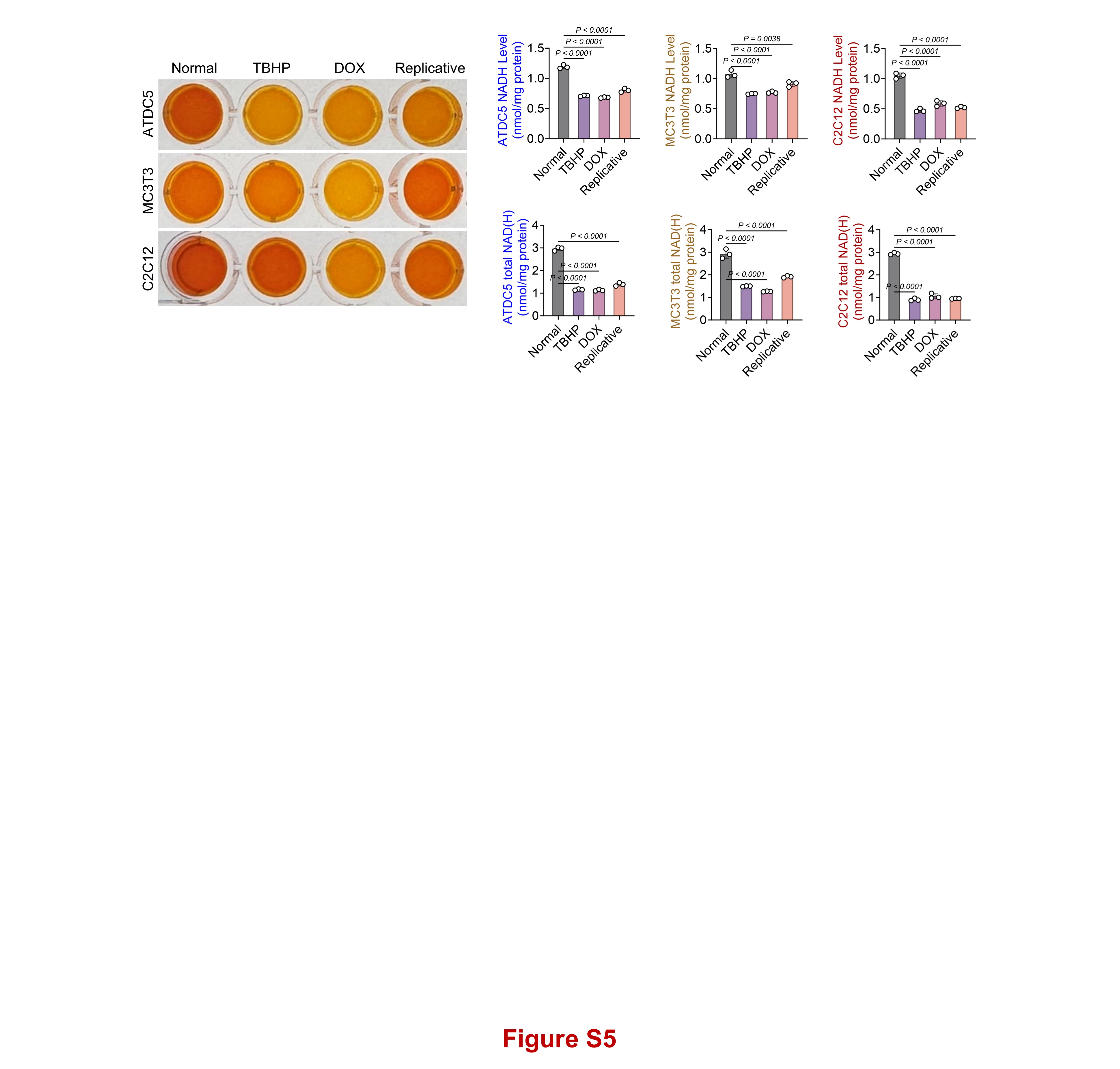


**Figure S5.** Detection results of NADH in ATDC5, MC3T3, and C2C12 cells under various treatment conditions (n = 3). Data are expressed as mean ± SD. Statistical significance was determined using one-way ANOVA, with significant differences between groups indicated by *P* < 0.05.


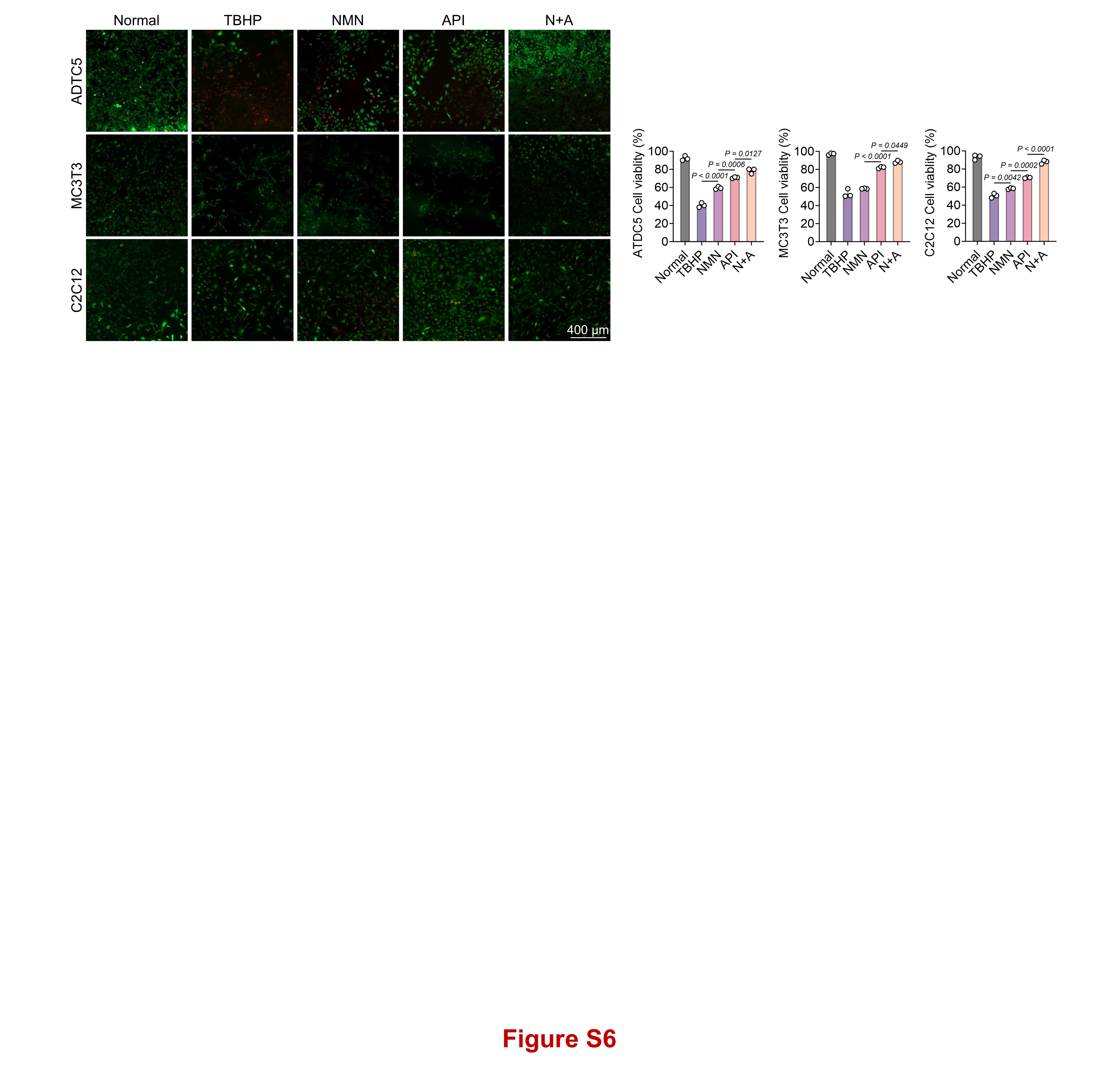


**Figure S6.** Live-dead staining in ATDC5, MC3T3, and C2C12 cells under various treatment conditions (n = 3). Data are expressed as mean ± SD. Statistical significance was determined using one-way ANOVA, with significant differences between groups indicated by *P* < 0.05.


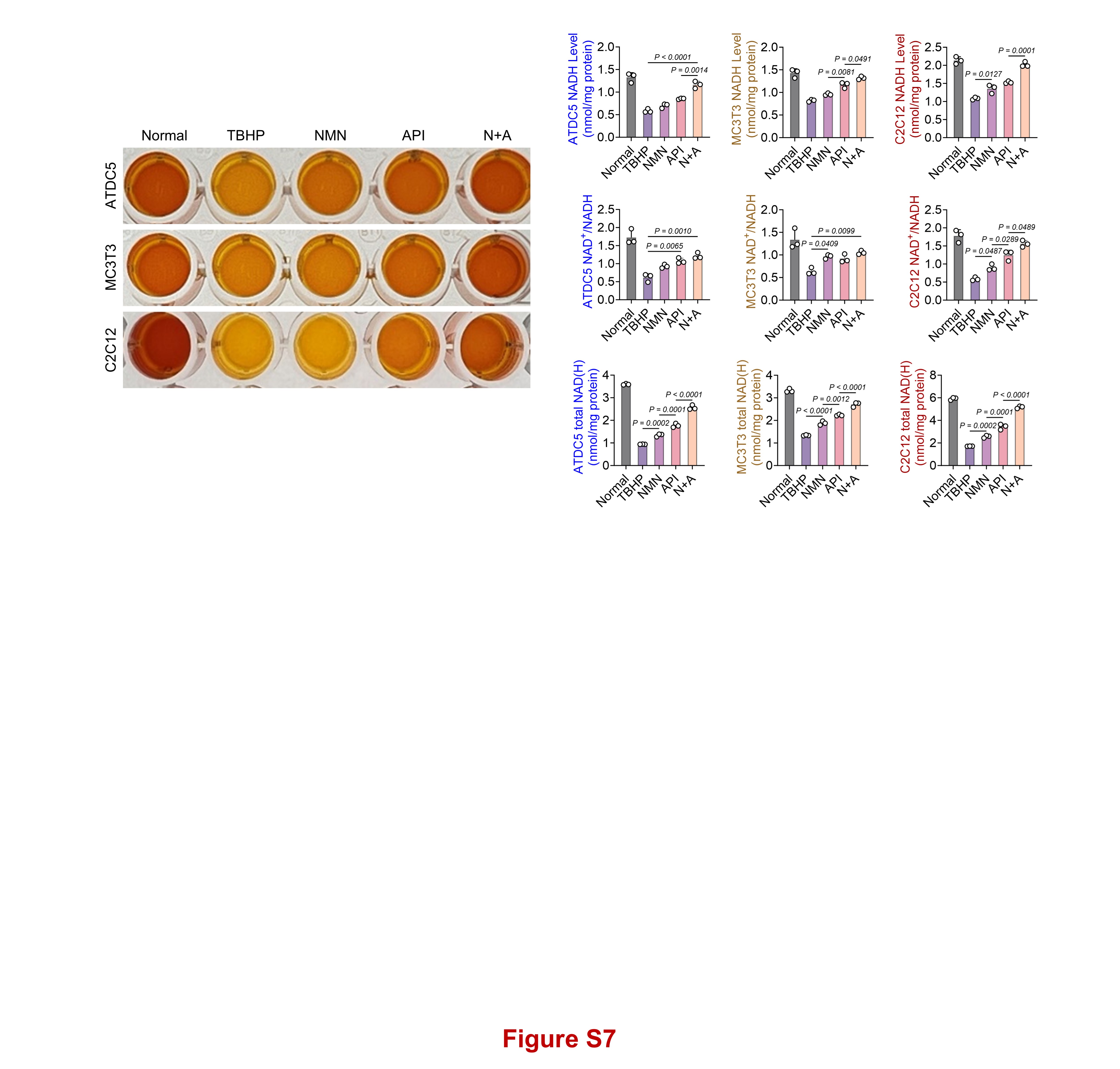


**Figure S7.** Detection results of NADH and NAD^+^/NADH in ATDC5, MC3T3, and C2C12 cells following different treatments (n = 3). Data are expressed as mean ± SD. Statistical significance was determined using one-way ANOVA, with significant differences between groups indicated by *P* < 0.05.


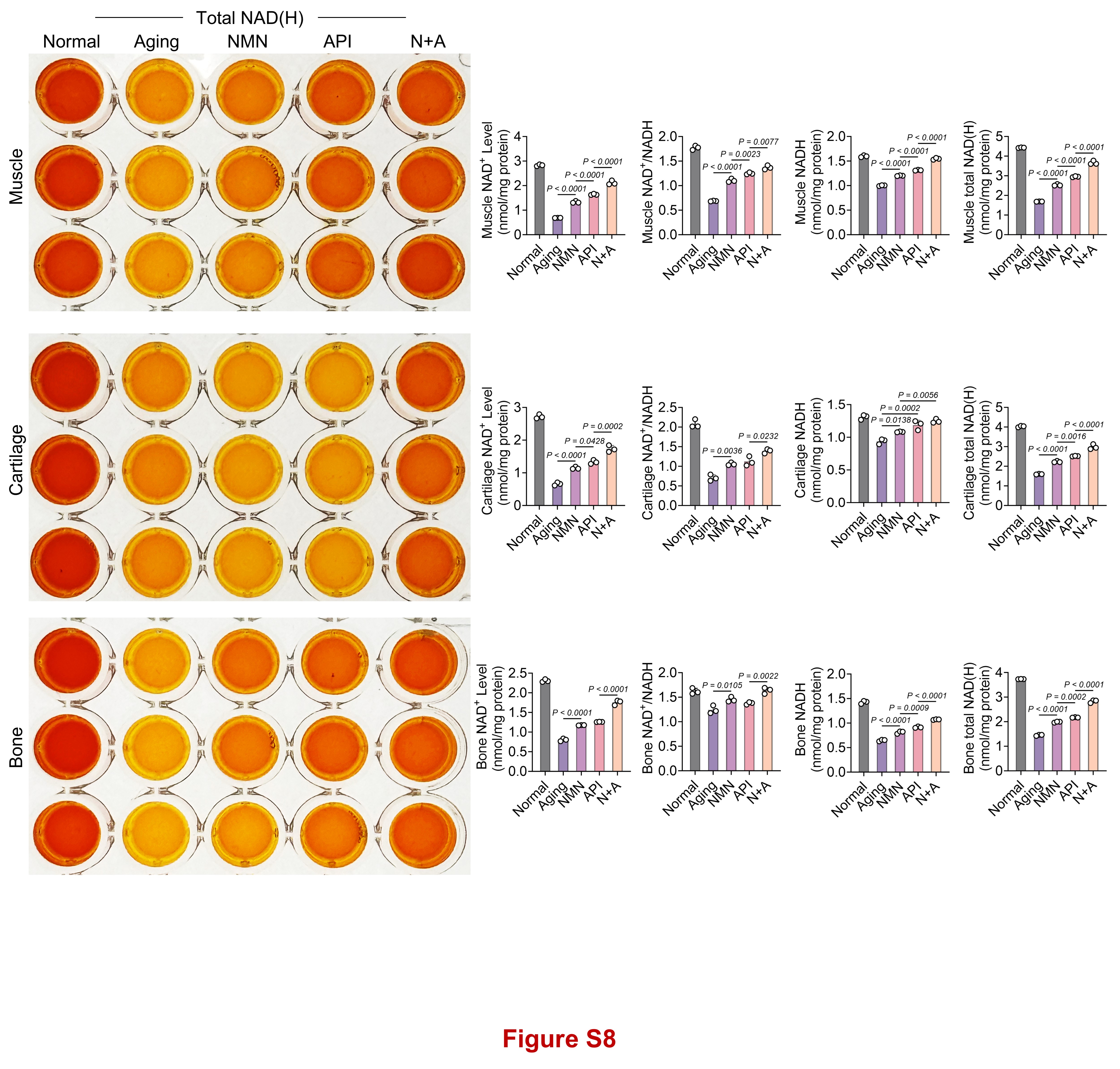


**Figure S8.** NAD⁺-boosting interventions restore NAD(H) metabolic homeostasis in aged muscle, cartilage, and bone tissues. Data are presented as mean ± SD and statistical significance is determined by one-way ANOVA. Statistically significant differences between the indicated groups are denoted by (*P* < 0.05).


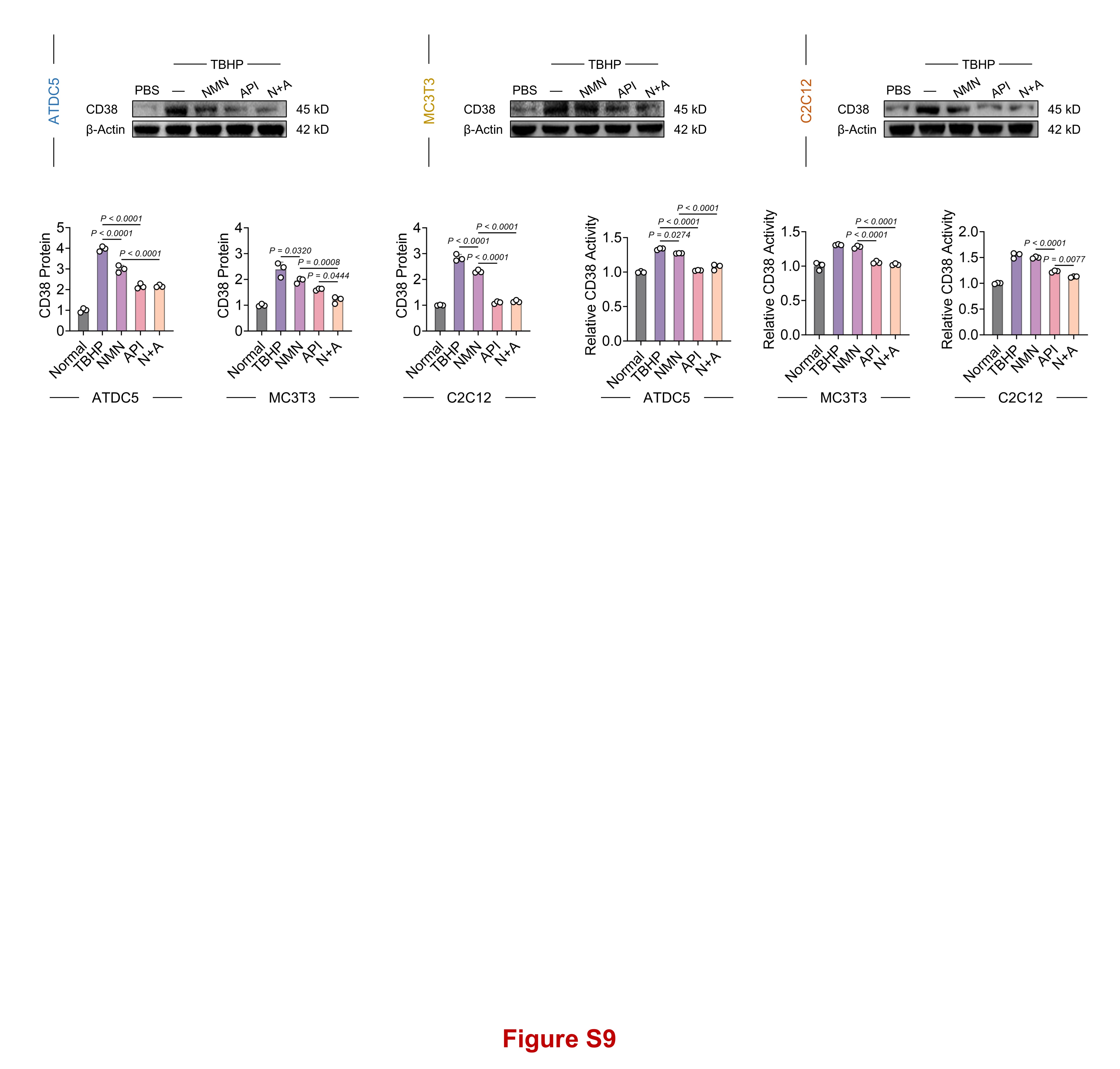


**Figure S9.** NAD⁺-boosting interventions modulate CD38 expression, activity assay and quantitative analysis in TBHP-induced cellular senescence models. Data are presented as mean ± SD and statistical significance is determined by one-way ANOVA. Statistically significant differences between the indicated groups are denoted by (*P* < 0.05).


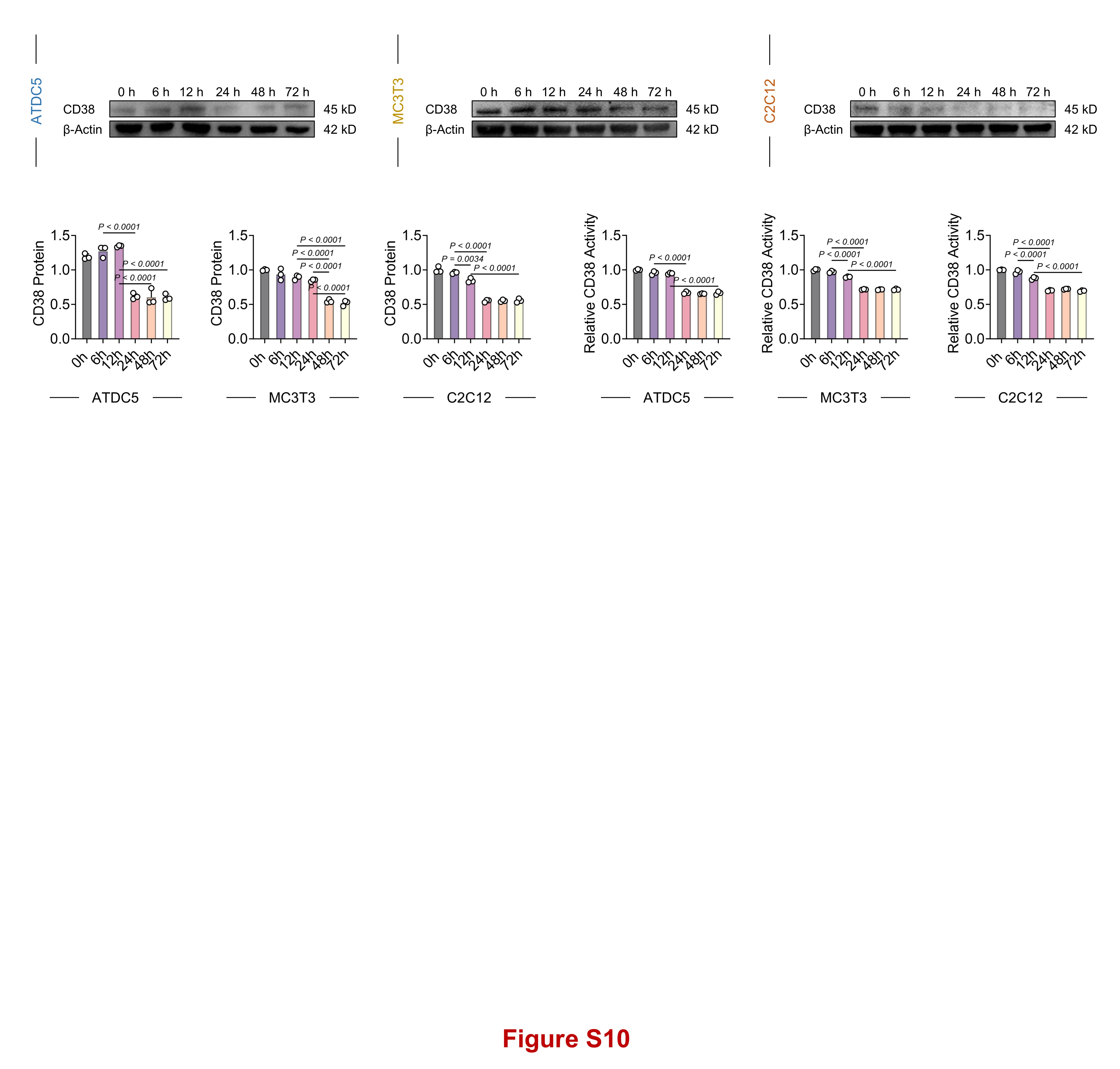


**Figure S10.** Time-dependent changes in CD38 protein expression and enzymatic activity and their quantitative analysis in ATDC5, MC3T3 and C2C12 cells by API treatment. Data are presented as mean ± SD and statistical significance is determined by one-way ANOVA. Statistically significant differences between the indicated groups are denoted by (*P* < 0.05).


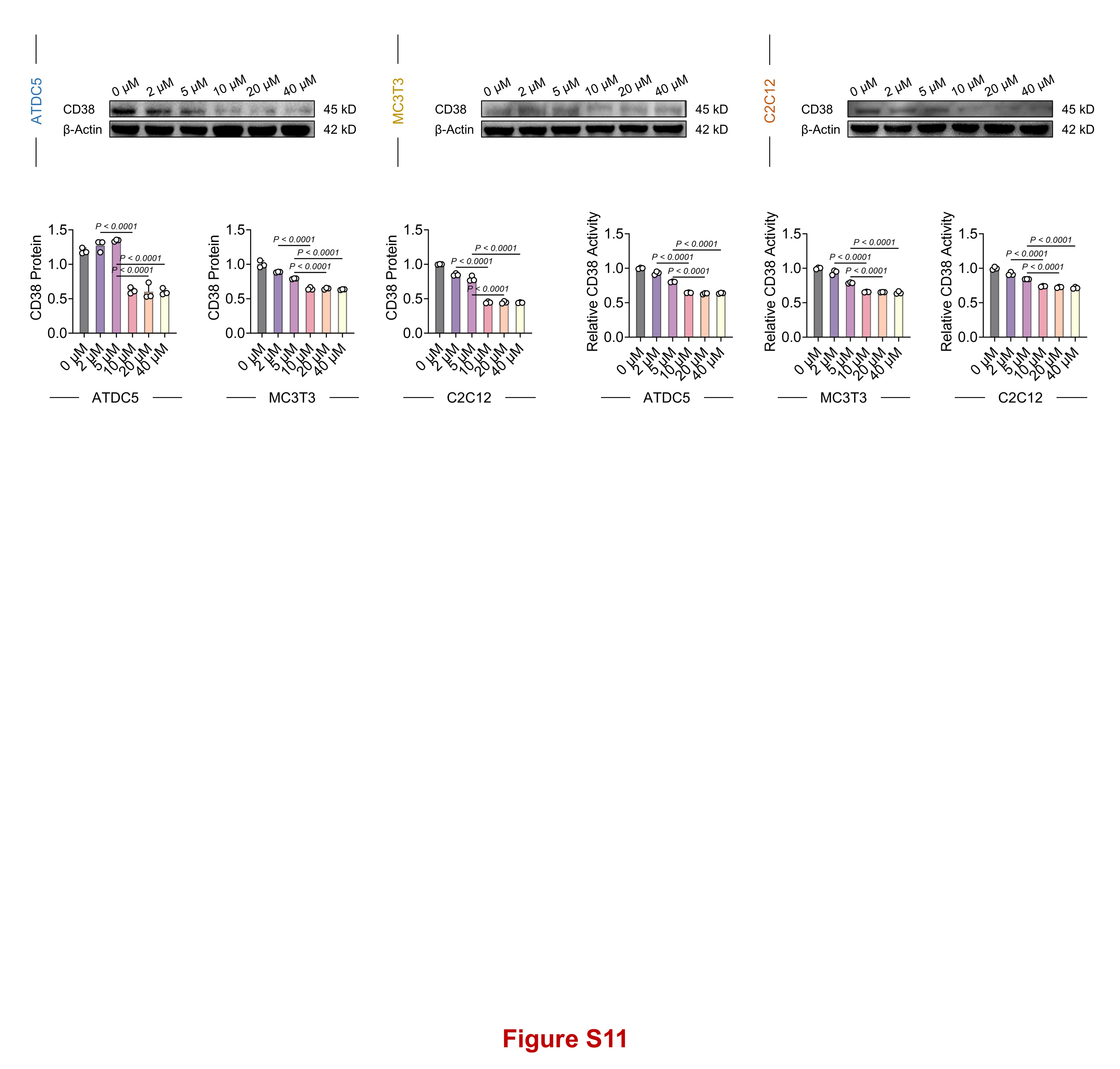


**Figure S11.** Concentration-dependent changes in CD38 protein expression and enzymatic activity and their quantitative analysis in ATDC5, MC3T3 and C2C12 cells by API treatment. Data are presented as mean ± SD and statistical significance is determined by one-way ANOVA. Statistically significant differences between the indicated groups are denoted by (*P* < 0.05).


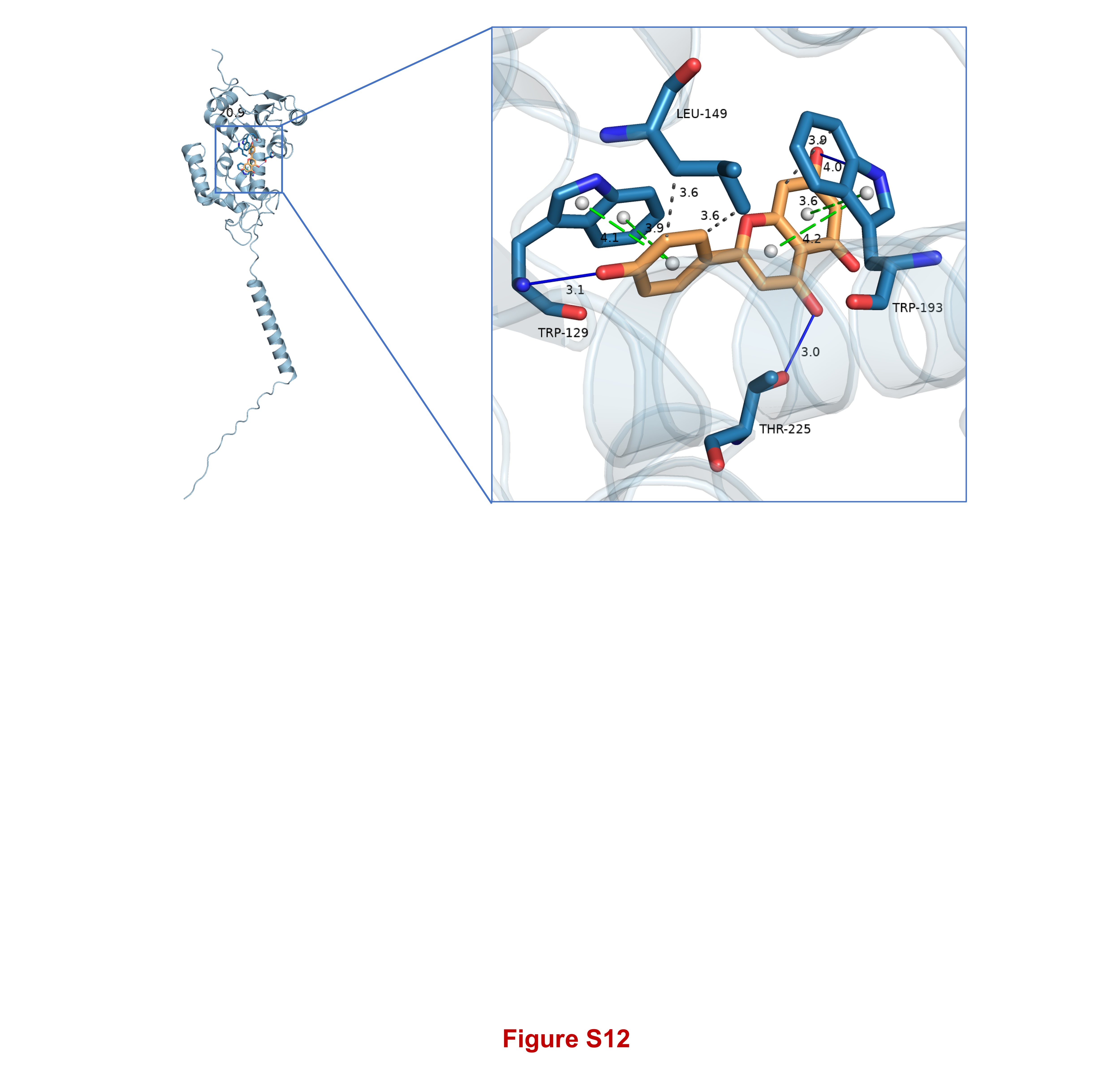


**Figure S12.** Molecular docking analysis of apigenin binding to the catalytic domain of CD38


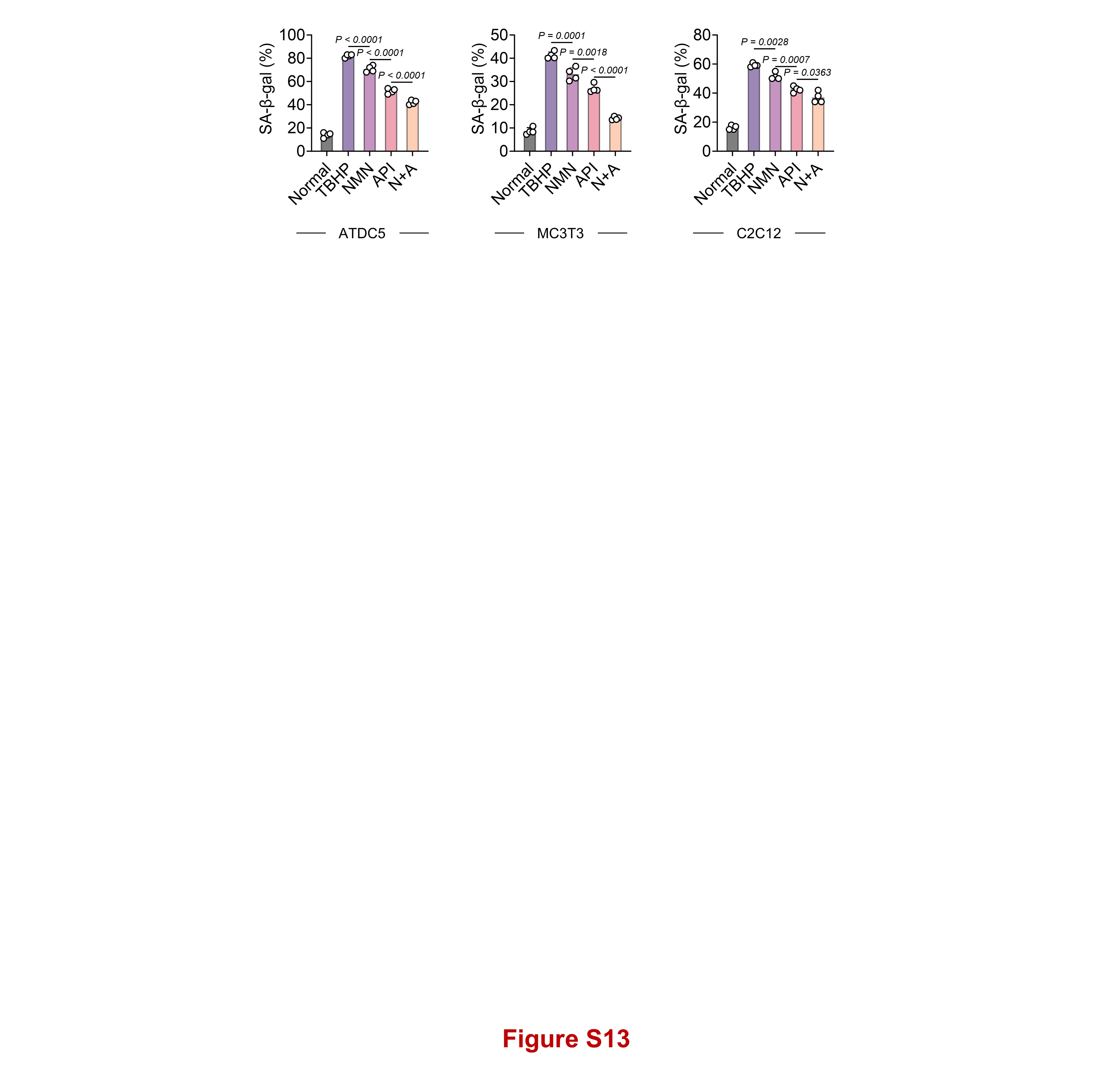


**Figure S13.** Percentage of SA-β-gal positive cells in ATDC5, MC3T3, and C2C12 cells following different treatments (n = 4). Data are expressed as mean ± SD. Statistical significance was determined using one-way ANOVA, with significant differences between groups indicated by *P* < 0.05.


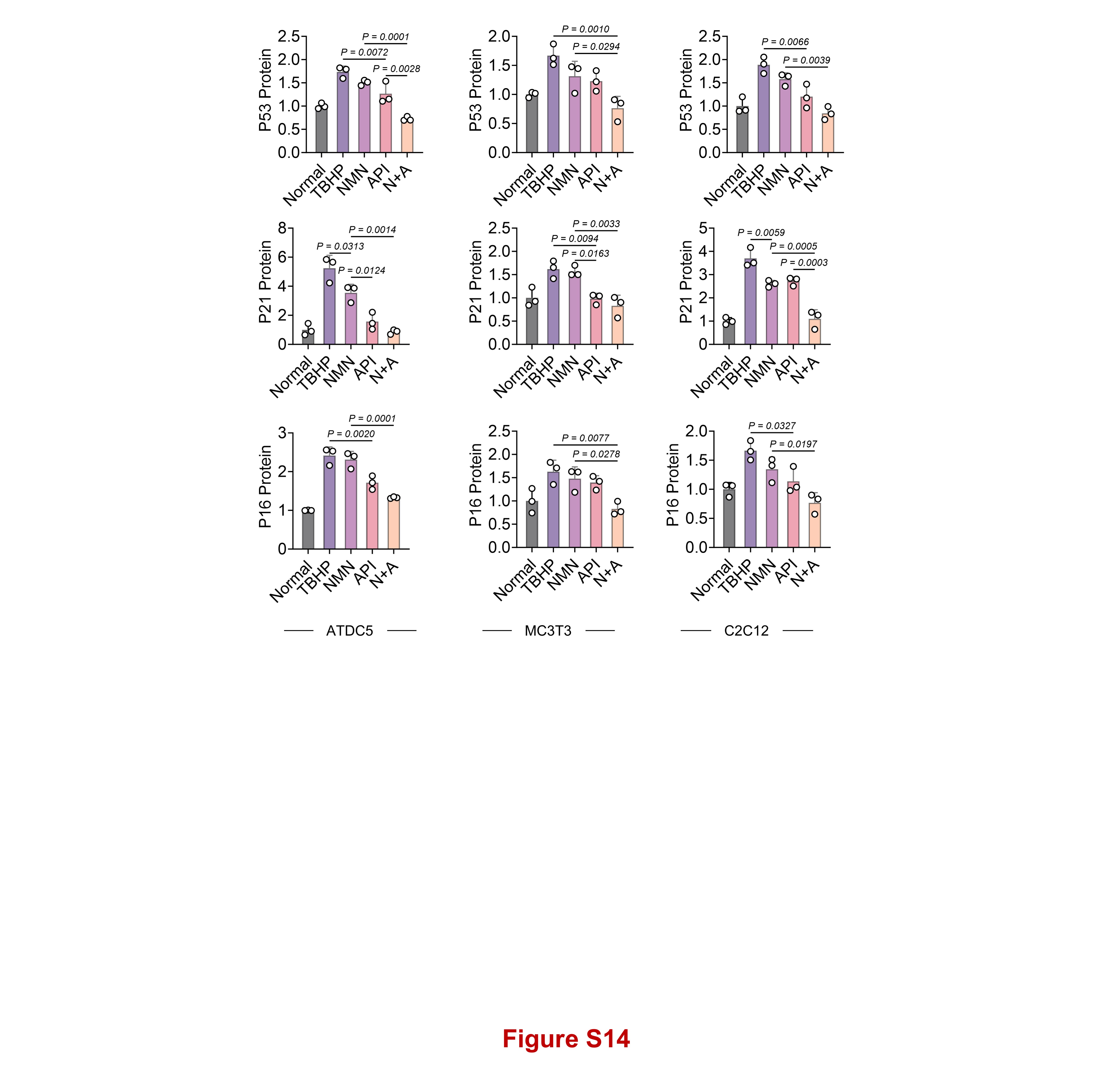


**Figure S14.** Quantification of P53, P21, and P16 at translational levels in ATDC5, MC3T3, and C2C12 cells following different treatments (n = 3). Data are expressed as mean ± SD. Statistical significance was determined using one-way ANOVA, with significant differences between groups indicated by *P* < 0.05.


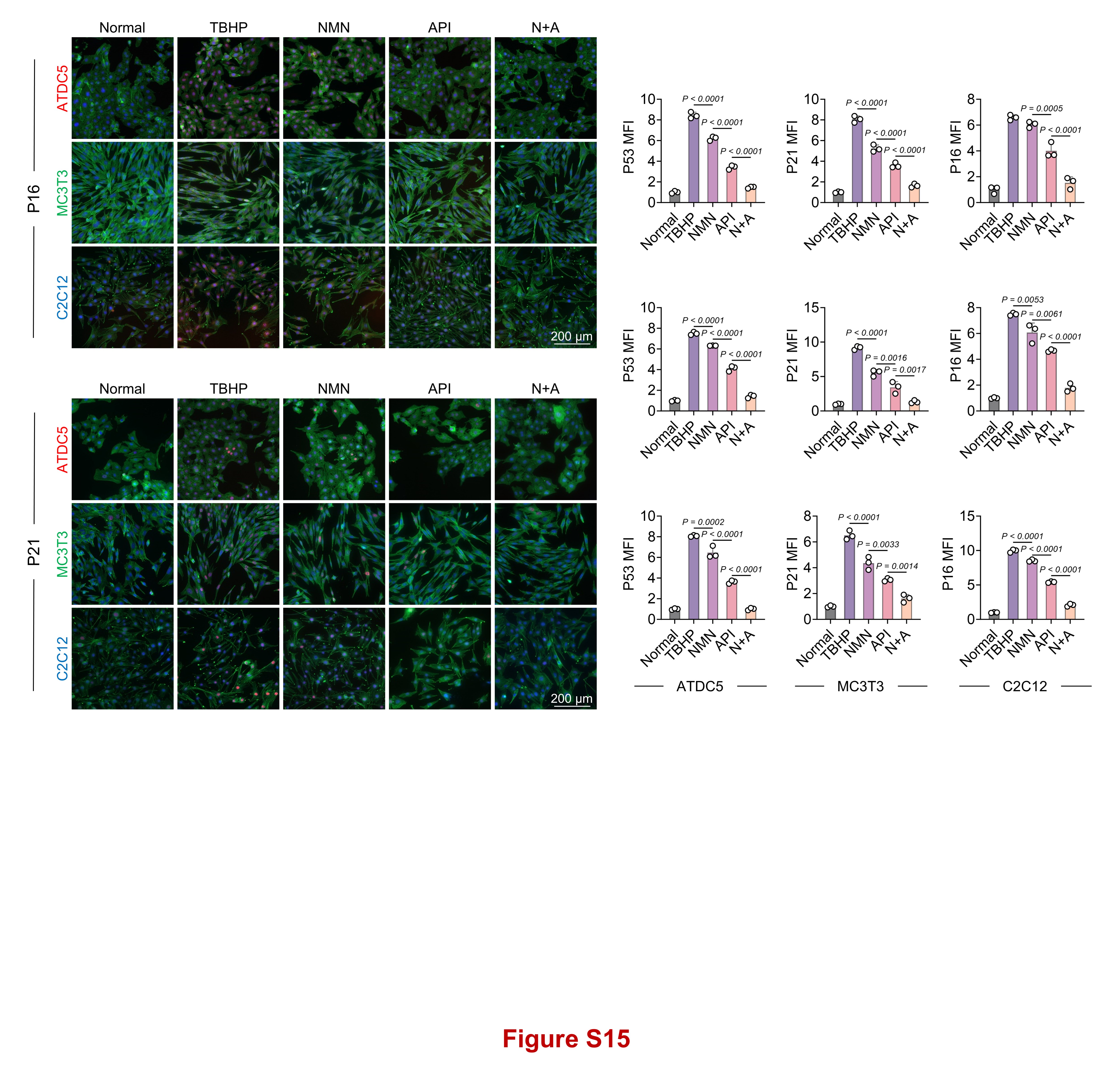


**Figure S15.** Representative immunofluorescence images and quantitative analysis of P21, and P16 positive cells in ATDC5, MC3T3, and C2C12 cells following different treatments (n = 3). Data are expressed as mean ± SD. Statistical significance was determined using one-way ANOVA, with significant differences between groups indicated by *P* < 0.05.


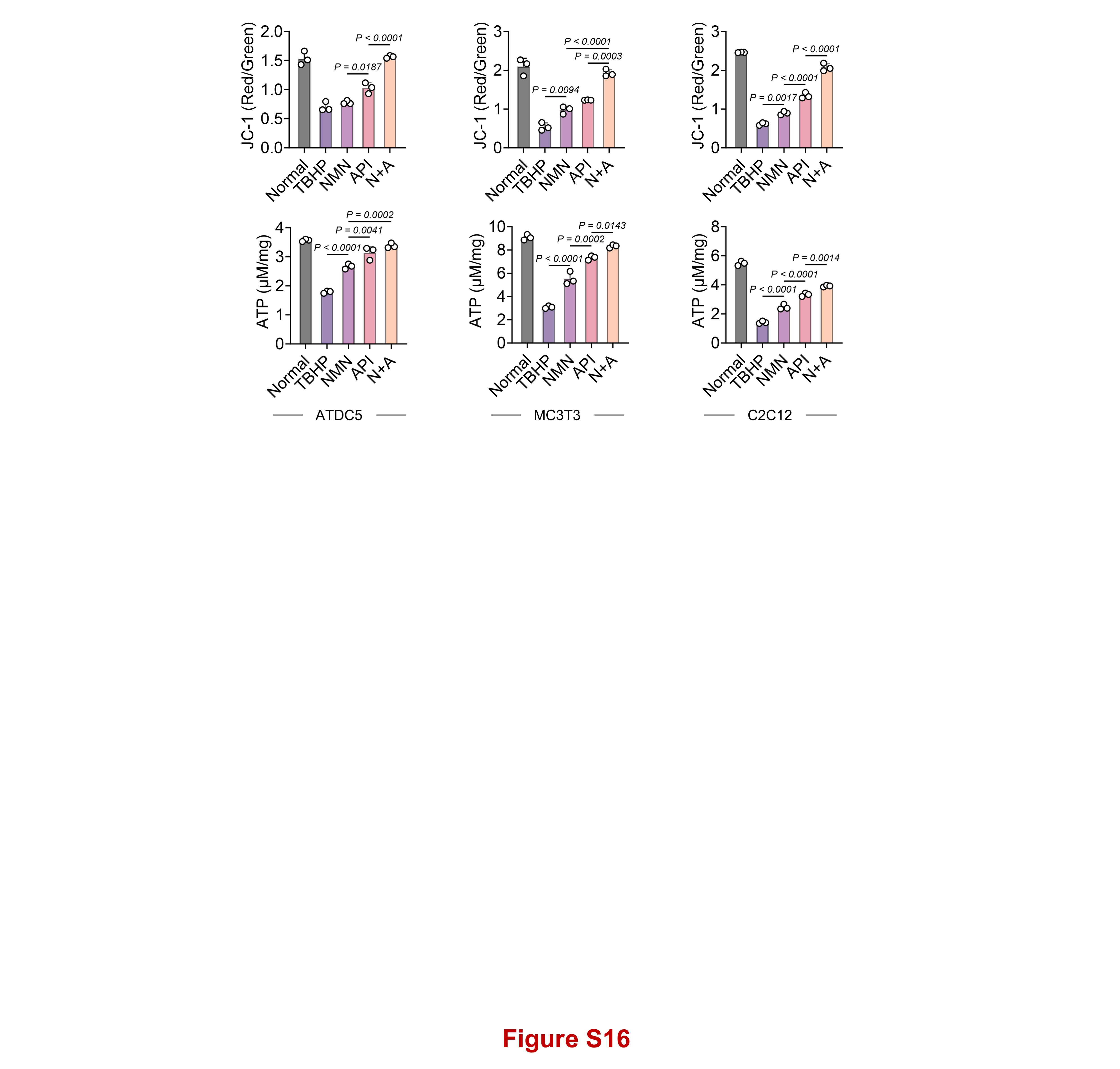


**Figure S16.** Quantitative analysis of JC-1 levels and ATP contents in ATDC5, MC3T3, and C2C12 cells following different treatments (n = 3). Data are expressed as mean ± SD. Statistical significance was determined using one-way ANOVA, with significant differences between groups indicated by *P* < 0.05.


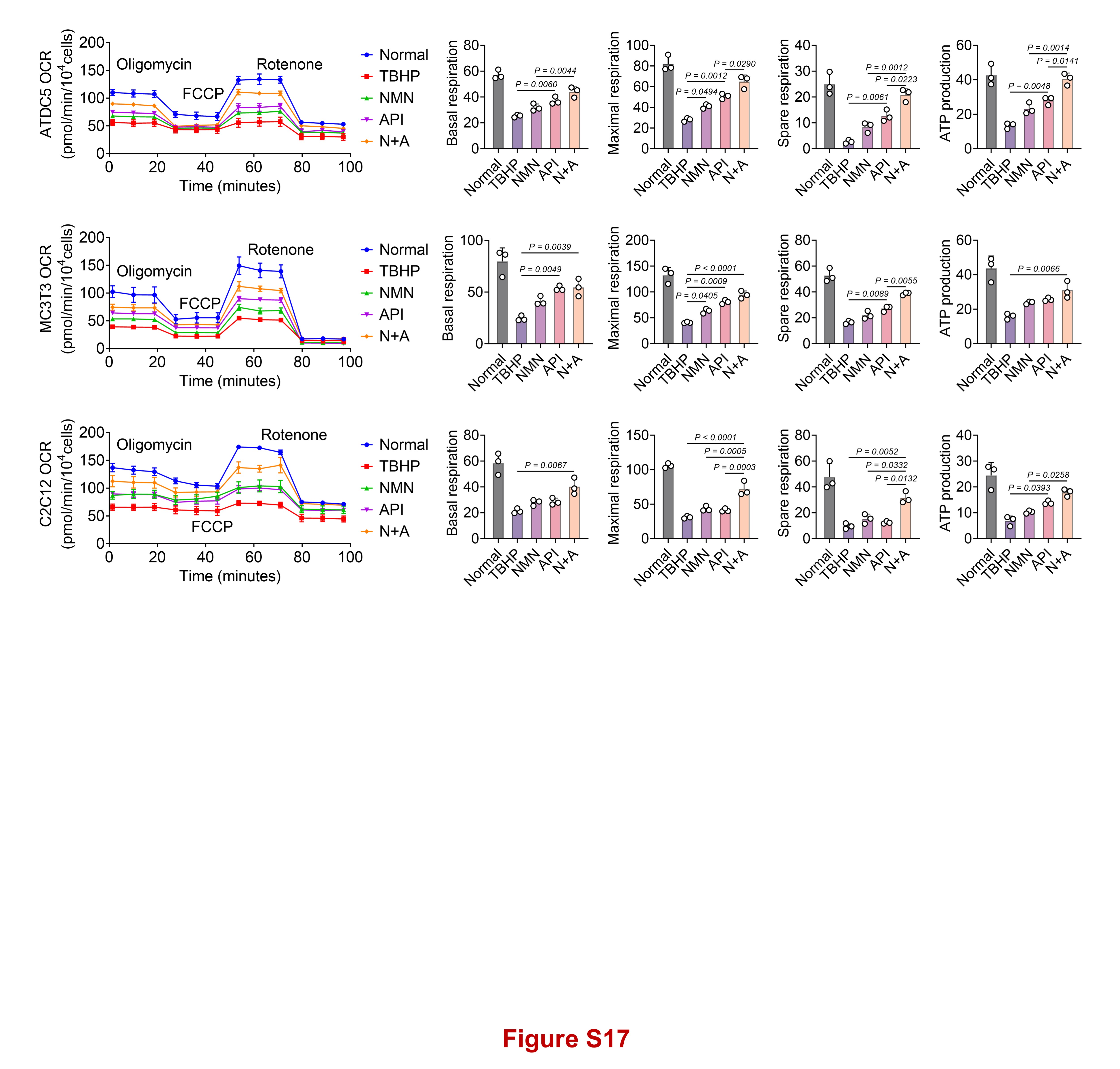


**Figure S17.** OCR measurements were performed using a Seahorse analyzer on senescent cells treated with N+A, and parameters such as basal respiration, maximal respiration, spare respiration, and ATP production of OCR were quantified. Data are presented as mean ± SD and statistical significance is determined by one-way ANOVA. Statistically significant differences between the indicated groups are denoted by (*P* < 0.05).


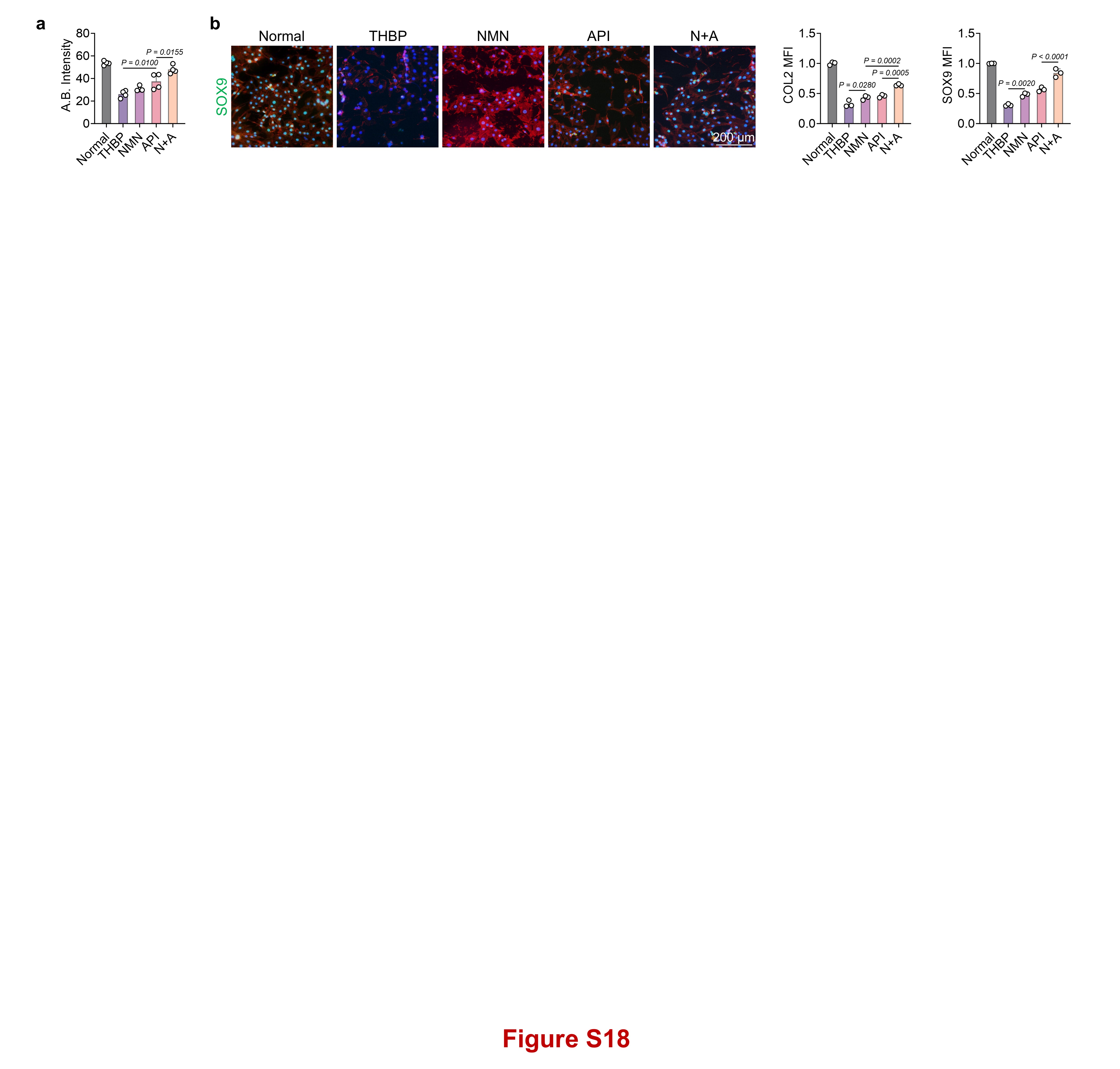


**Figure S18.** The N+A intervention enhances the matrix synthesis capacity of senescent chondrocytes. (a) Quantitative assessment of Alcian Blue (A.B.) staining intensity across various treatment groups; (b) Immunofluorescence staining and quantitative analysis of SOX9 expression (n = 3). Data are expressed as mean ± SD. Statistical significance was determined using one-way ANOVA, with significant differences between groups indicated by *P* < 0.05.

**
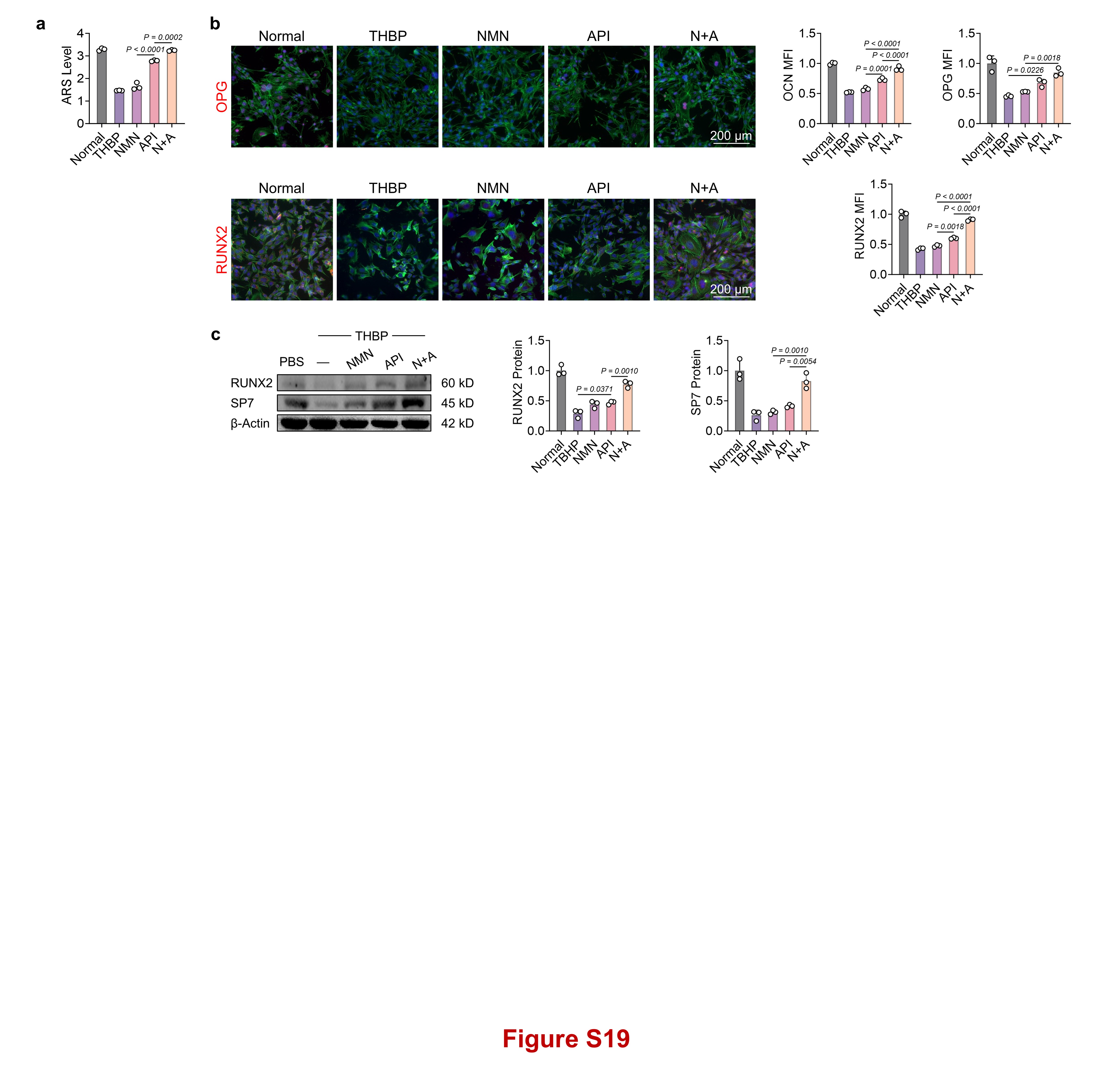
**

**Figure S19.** NAD^+^ supplementation enhances the osteogenic differentiation of MC3T3 cells. (a) Quantitative analysis of Alizarin Red S (ARS) staining during osteogenic differentiation of MC3T3 cells (n = 3). (b) Representative immunofluorescence images and corresponding quantitative analysis of OPG and RUNX2 expression during osteogenic differentiation. (c) Representative images and quantitative assessment of protein expression levels associated with osteogenic differentiation (n = 3). Data are expressed as mean ± SD. Statistical significance was determined using one-way ANOVA, with significant differences between groups indicated by *P* < 0.05.


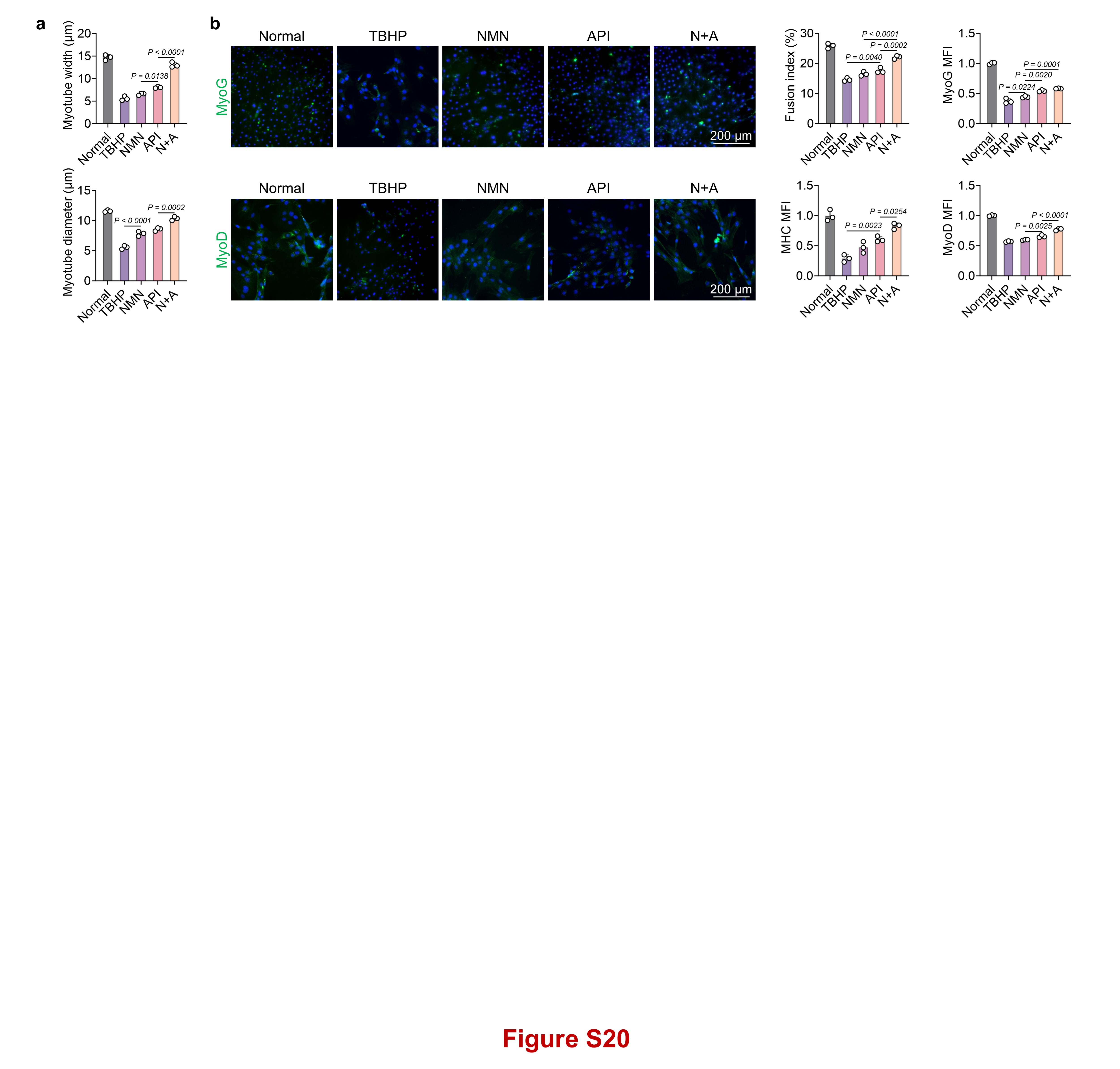


**Figure S20.** Supplementation with NAD+ enhances the myogenic differentiation of C2C12 cells. (a) Quantitative analysis of Giemsa staining and myosin heavy chain (MHC) immunofluorescence in C2C12 cells (n = 3). (b) Representative images and quantitative assessment of MyoG and MyoD immunofluorescence staining during myogenic differentiation (n = 3). Data are expressed as mean ± SD. Statistical significance was determined using one-way ANOVA, with significant differences between groups indicated by *P* < 0.05.


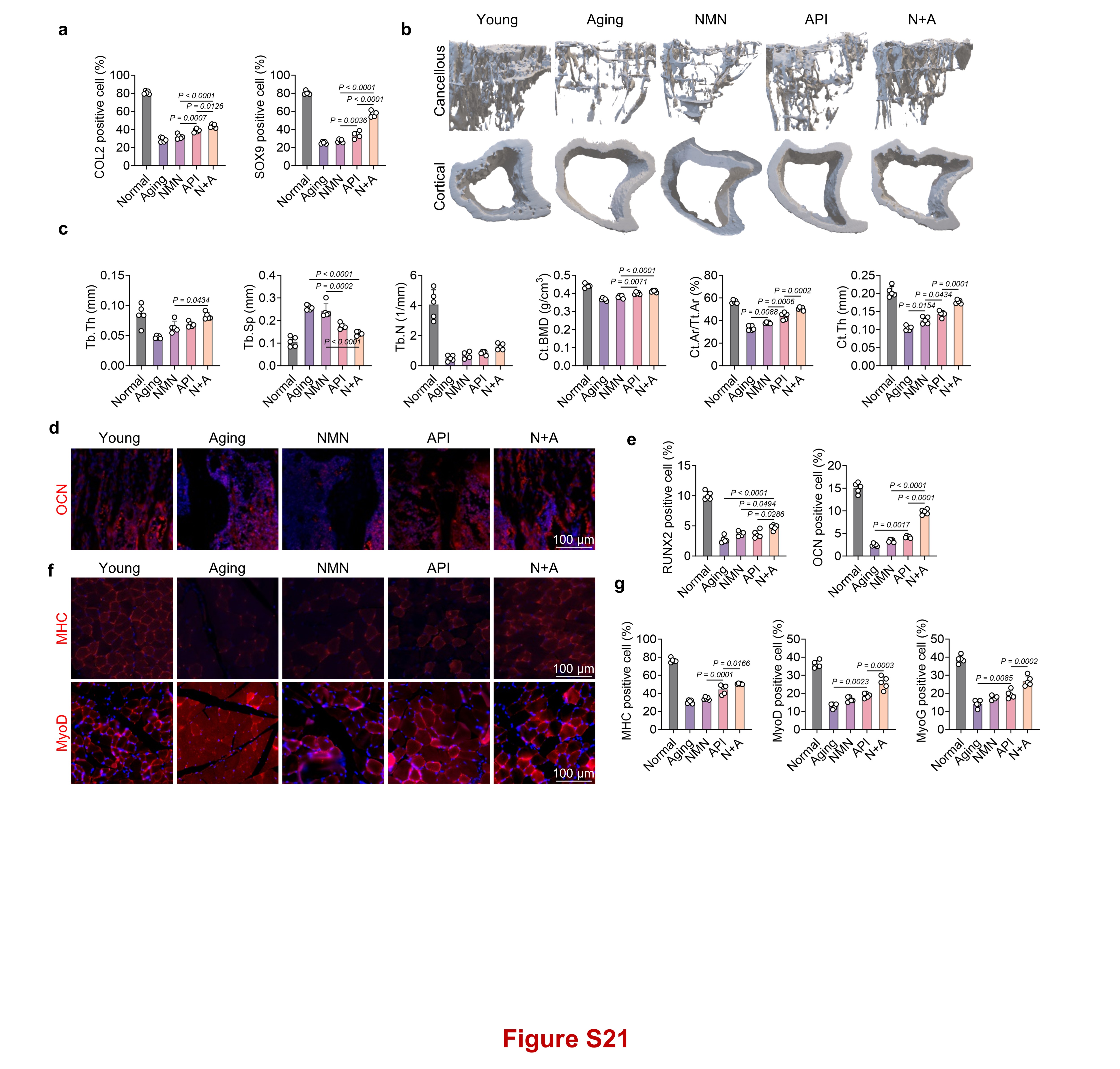


**Figure S21.** The effects of NAD^+^ supplementation on bone and muscle tissues were assessed. (a) Quantitative analysis of COL2 and SOX9 expression was performed using immunofluorescence staining on mouse tissue sections (n = 5). (b–c) Micro-computed tomography (μCT) was employed to generate three-dimensional reconstructions of trabecular and cortical bones in mouse tibias. Structural parameters, including trabecular thickness (Tb.Th), trabecular number (Tb.N), trabecular separation (Tb.Sp), cortical bone mineral density (Ct.BMD), cortical area to total area ratio (Ct.Ar/Tt.Ar), and cortical thickness (Ct.Th), were quantitatively evaluated using μCT (n = 5). (d) Representative immunofluorescence staining images of osteocalcin (OCN) are shown. (e) Quantitative analysis of RUNX2 and OCN expression levels was conducted (n = 5). (f–g) Representative immunofluorescence staining images of muscle-specific markers, including myosin heavy chain (MHC) and MyoD, were obtained from muscle tissues, followed by quantitative analysis of MHC, MyoD, and MyoG expression levels (n = 5). Data are expressed as mean ± SD. Statistical significance was determined using one-way ANOVA, with significant differences between groups indicated by *P* < 0.05.


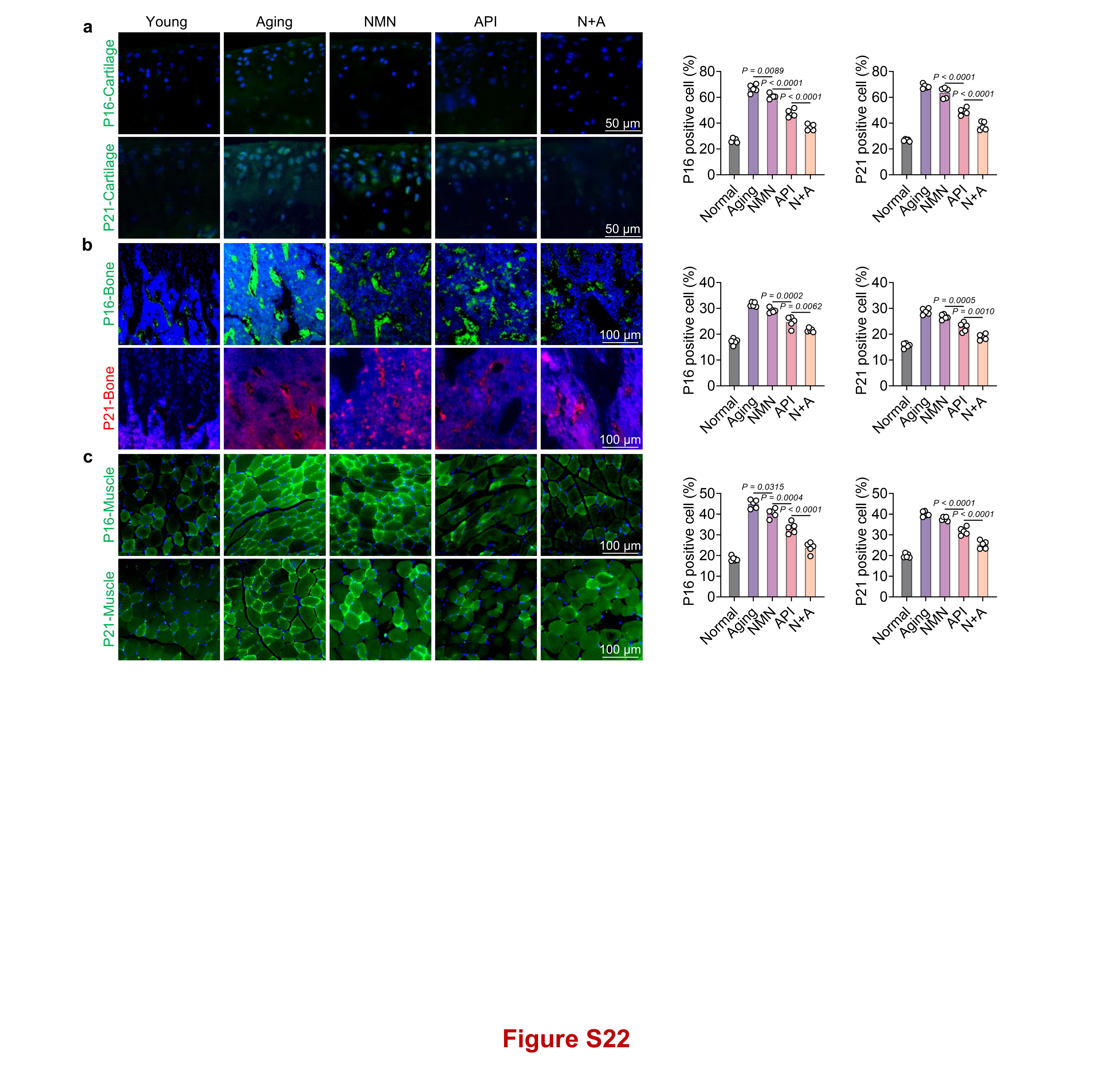


**Figure S22.** NAD^+^ supplementation attenuates markers of cellular senescence *in vivo*. (a–c) Representative images and quantitative analysis of P16 and P21 immunofluorescence staining in mouse cartilage, bone, and skeletal muscle tissues (n = 5). Data are expressed as mean ± SD. Statistical significance was determined using one-way ANOVA, with significant differences between groups indicated by *P* < 0.05.


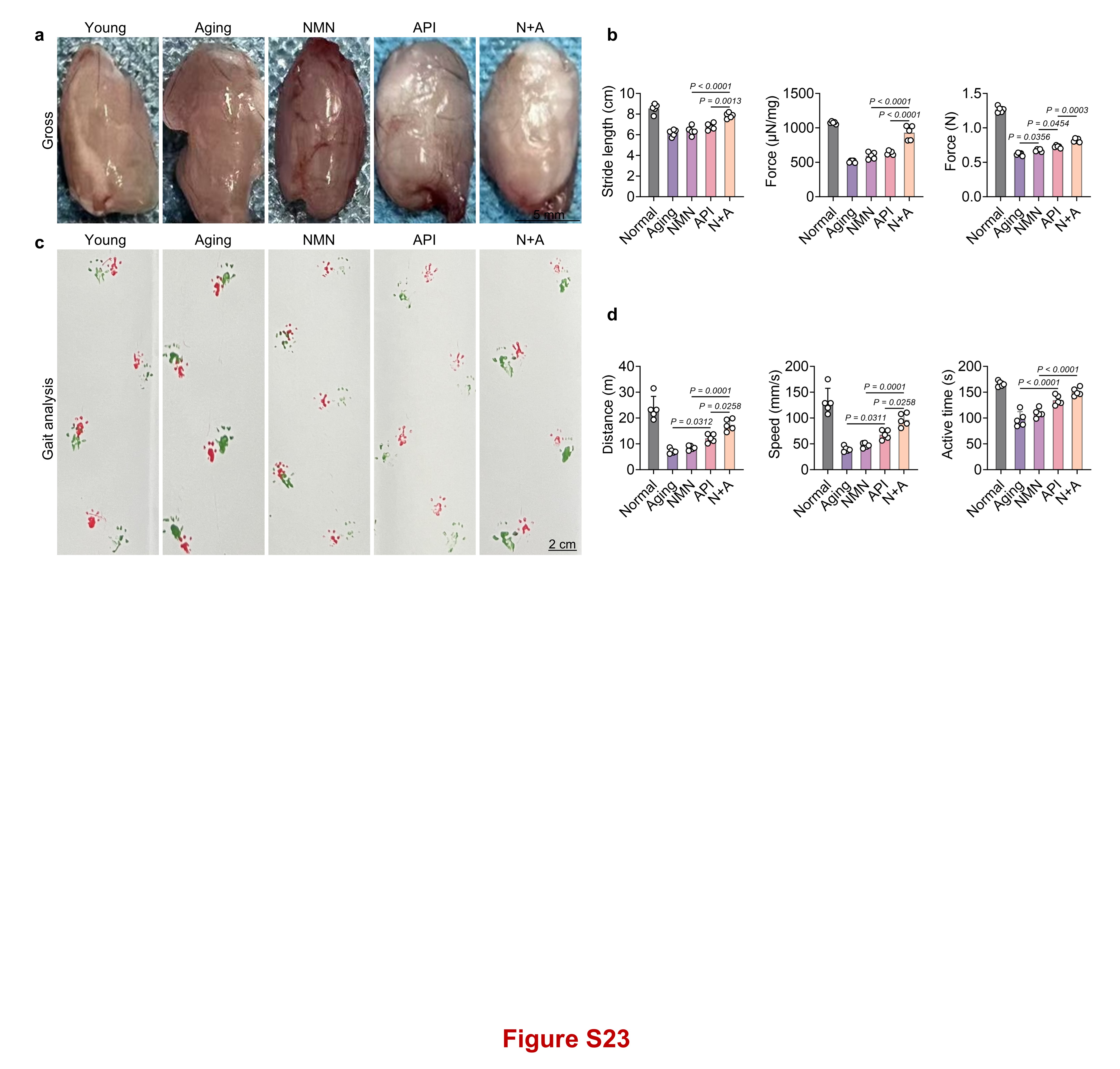


**Figure S23.** The effects of NAD^+^ supplementation on muscle function and motor performance *in vivo*. (a) Gross anatomical view of the quadriceps femoris in mice. (b) Gait analysis, lower limb muscle strength assessment, and upper limb grip strength measurement in mice (n = 5). (c) Representative gait analysis images. (d) Quantitative evaluation of movement distance, locomotor speed, and activity duration in the open field test (n = 5). Statistical significance was determined using one-way ANOVA, with significant differences between groups indicated by *P* < 0.05.


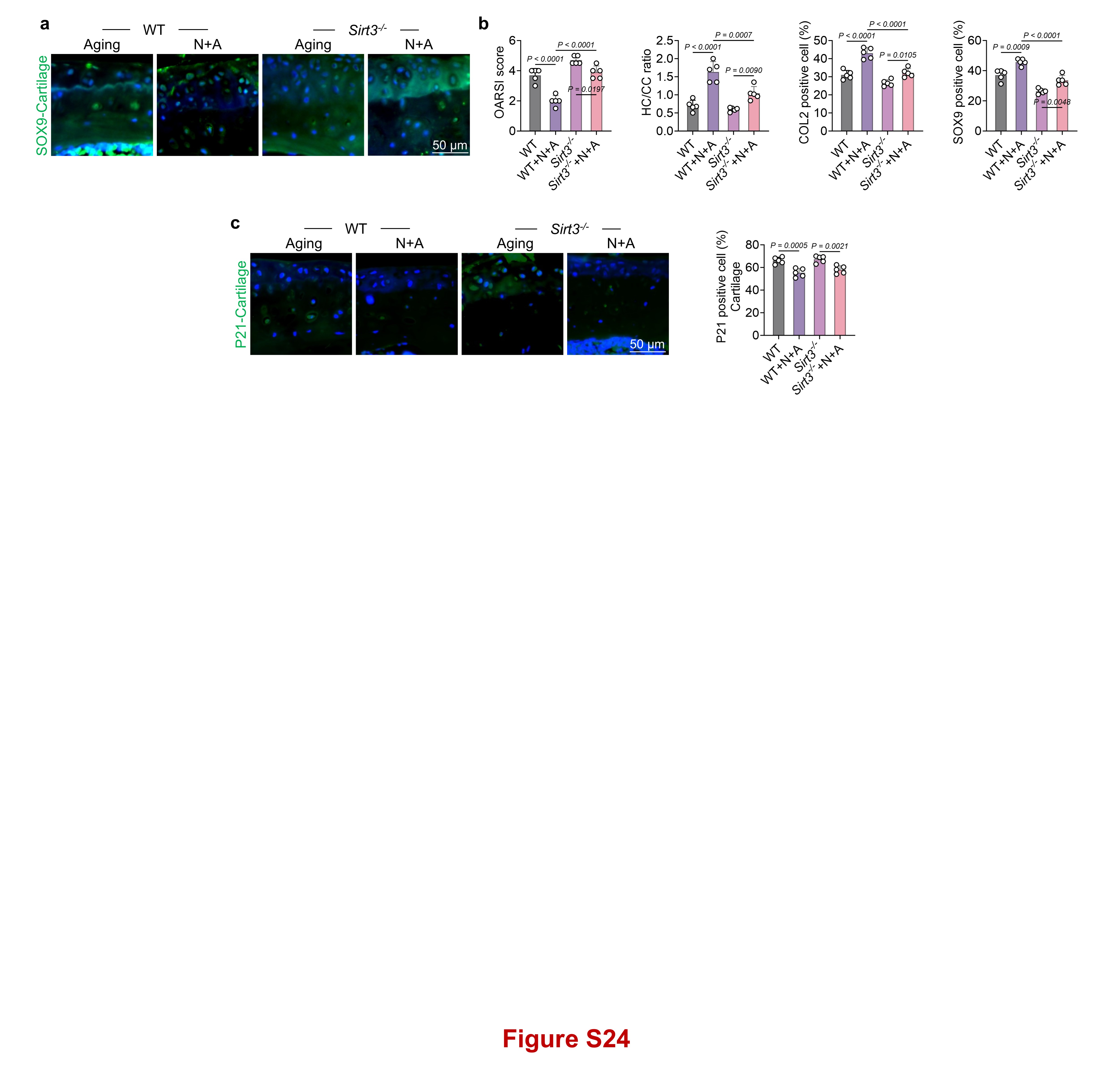


**Figure S24.** The effect of NAD^+^ supplementation on age-related cartilage lesions in SIRT3-knockout mice. (a) Representative images of Sox9 immunofluorescence staining (n = 5). (b) Quantitative analysis of OARSI scores, HC/CC ratios, and the percentage of COL2- and SOX9-positive cells based on immunofluorescence staining (n = 5). (c) Representative images and quantification of P21 immunofluorescence staining (n = 5). Statistical significance was determined using one-way ANOVA, with significant differences between groups indicated by *P* < 0.05.


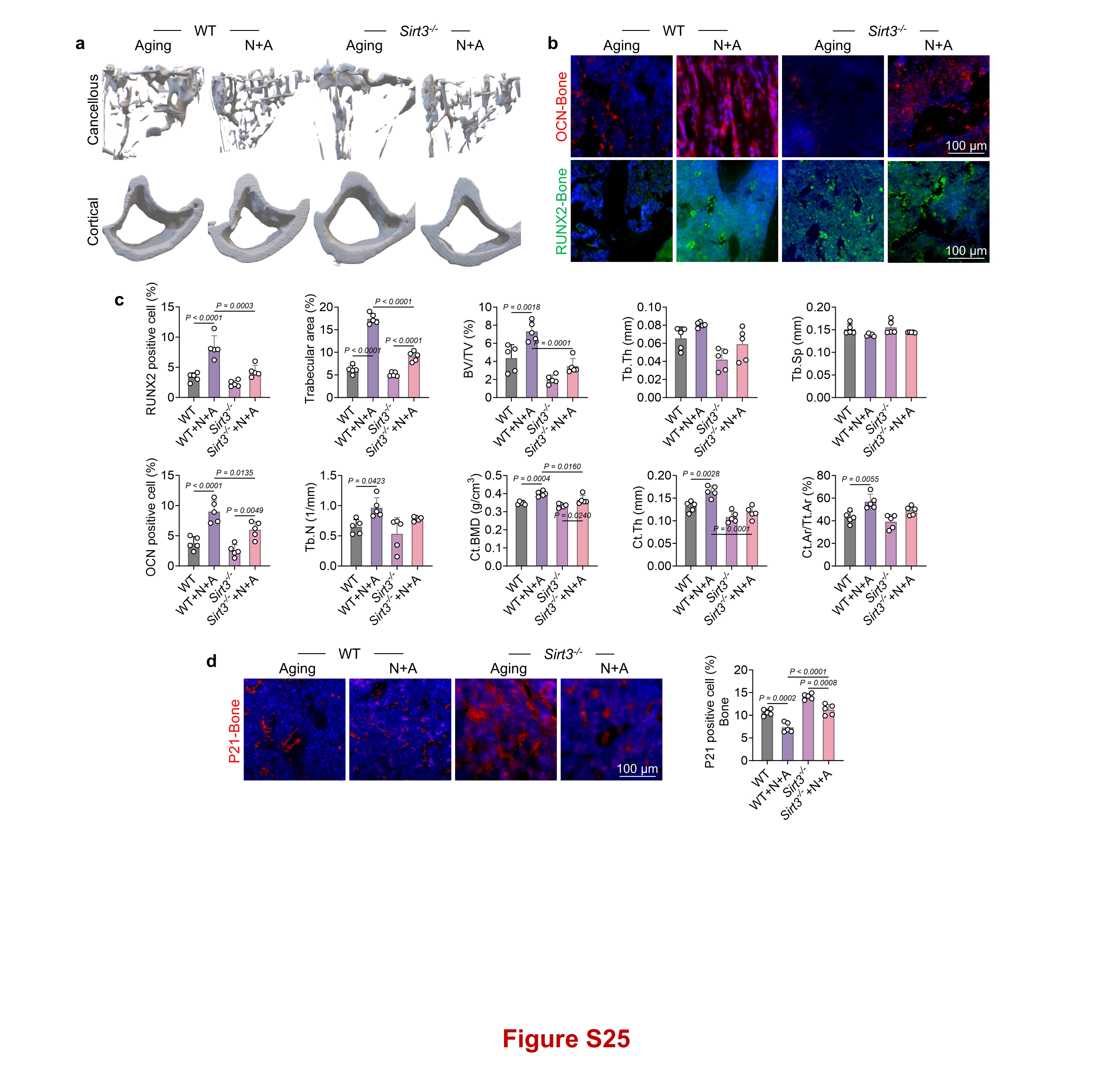


**Figure S25.** Effects of NAD^+^ supplementation on age-related bone changes in SIRT3-knockout mice. (a) μCT three-dimensional reconstruction of trabecular and cortical bones in the tibia following SIRT3 knockout and pharmacological treatment (n = 5). (b) Representative images of OCN and RUNX2 immunofluorescence staining. (c) Quantitative analysis of trabecular bone thickness (Tb.Th), trabecular number (Tb.N), trabecular separation (Tb.Sp), cortical bone mineral density (Ct.BMD), cortical bone area relative to total area (Ct.Ar/Tt.Ar), and cortical bone thickness (Ct.Th), as assessed by μCT, OCN and RUNX2 immunofluorescence staining, and trabecular bone quantification (n = 5). (d) Representative images and quantitative results of P21 immunofluorescence staining (n = 5). Statistical significance was determined using one-way ANOVA, with significant differences between groups indicated by *P* < 0.05.


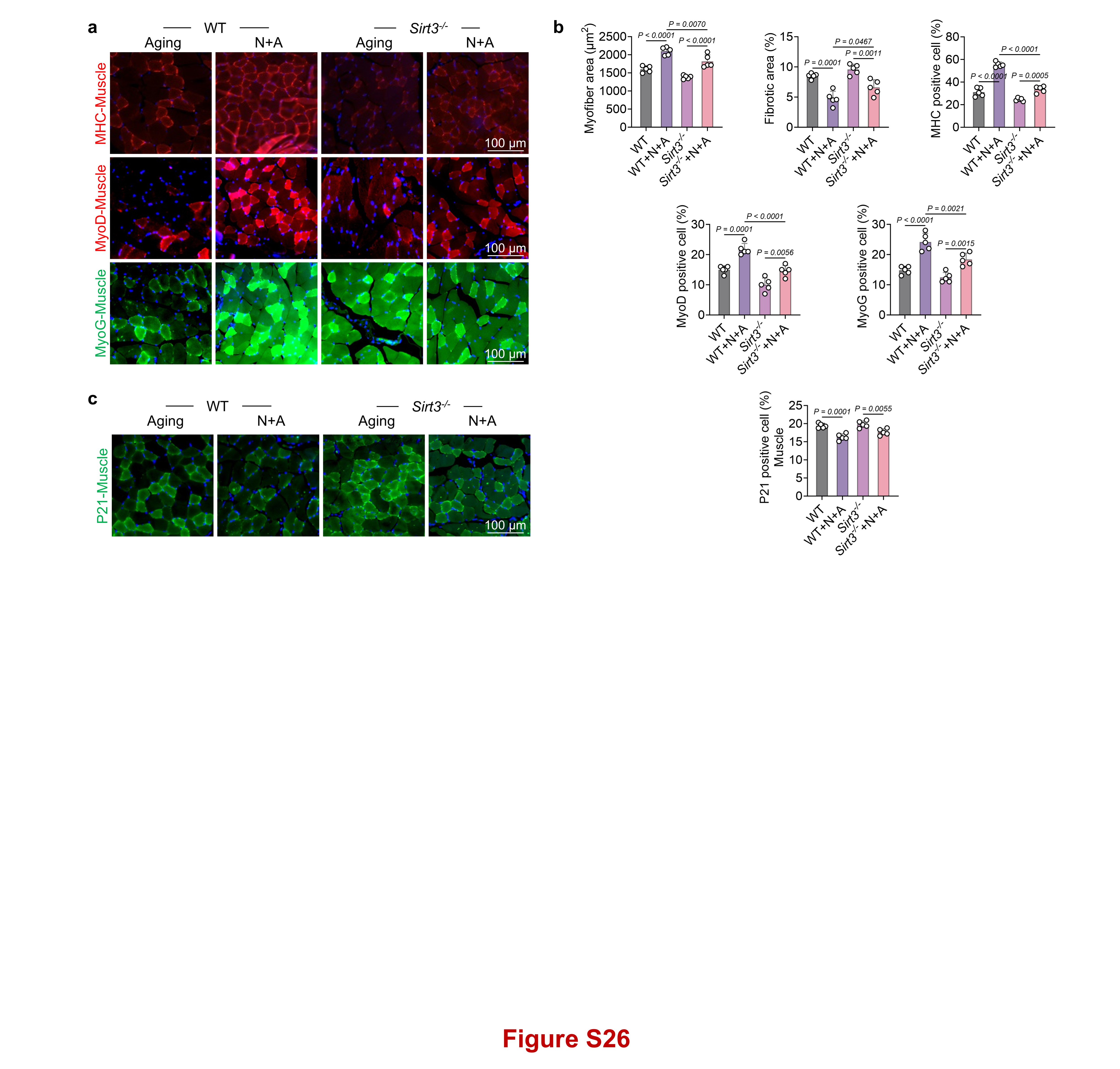


**Figure S26.** Effects of NAD^+^ supplementation on age-related muscle changes in SIRT3-knockout mice. (a-c) Representative images and quantitative analysis of MHC, MyoD, MyoG, and p21 immunofluorescence staining in mouse quadriceps muscles (n = 5). Statistical significance was determined using one-way ANOVA, with significant differences between groups indicated by *P* < 0.05.


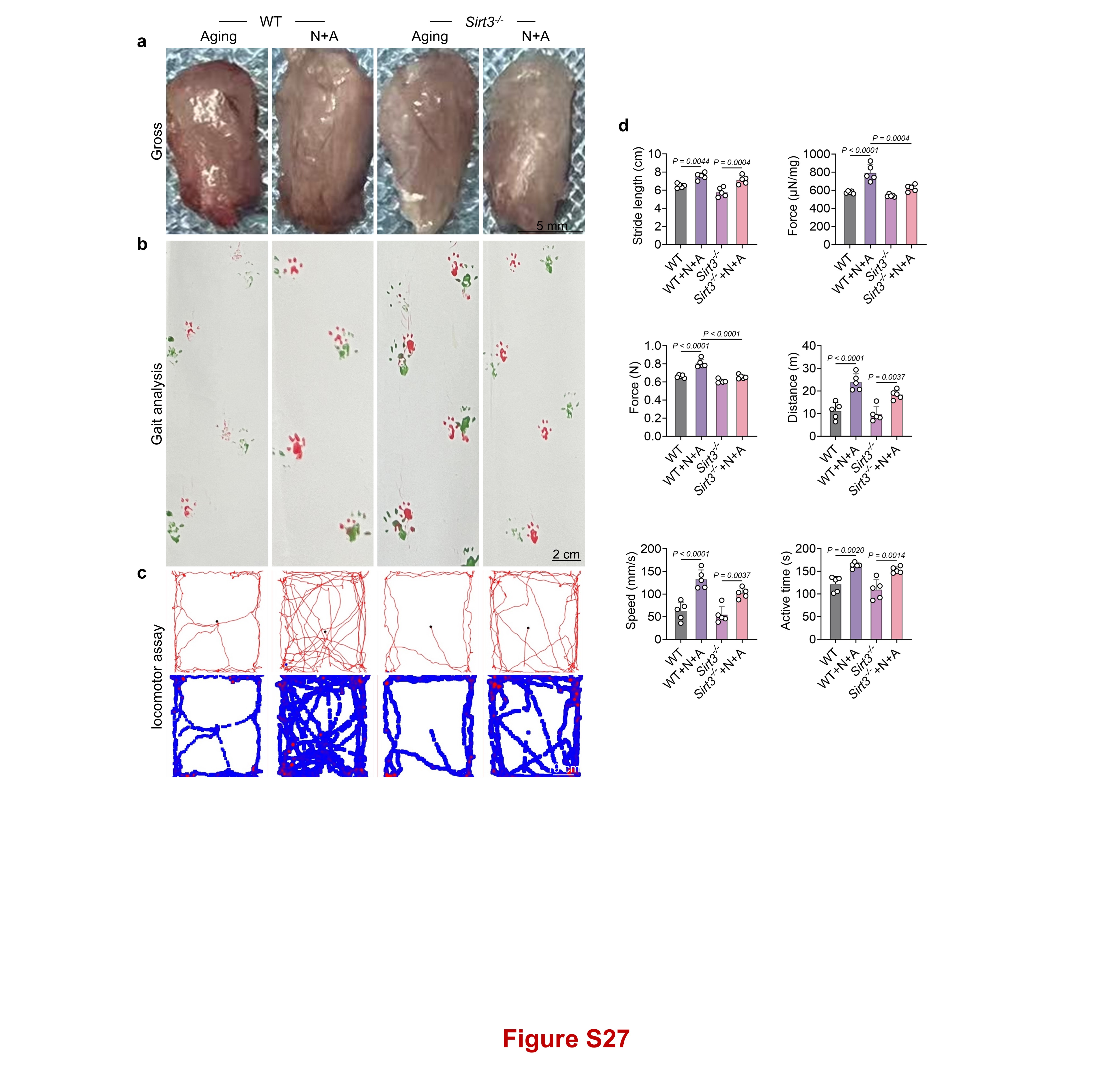


**Figure S27.** Effects of NAD^+^ supplementation on muscle function and motor performance in SIRT3-knockout mice. (a) Gross anatomical view of the quadriceps femoris muscle in mice. (b) Representative images obtained from gait analysis. (c) Representative recordings from the open field test. (d) Quantitative assessment of gait parameters, muscle strength, and forelimb grip strength, as well as total moving distance, average speed, and activity duration in the open field test in mice (n = 5). Statistical significance was determined using one-way ANOVA, with significant differences between groups indicated by *P* < 0.05.


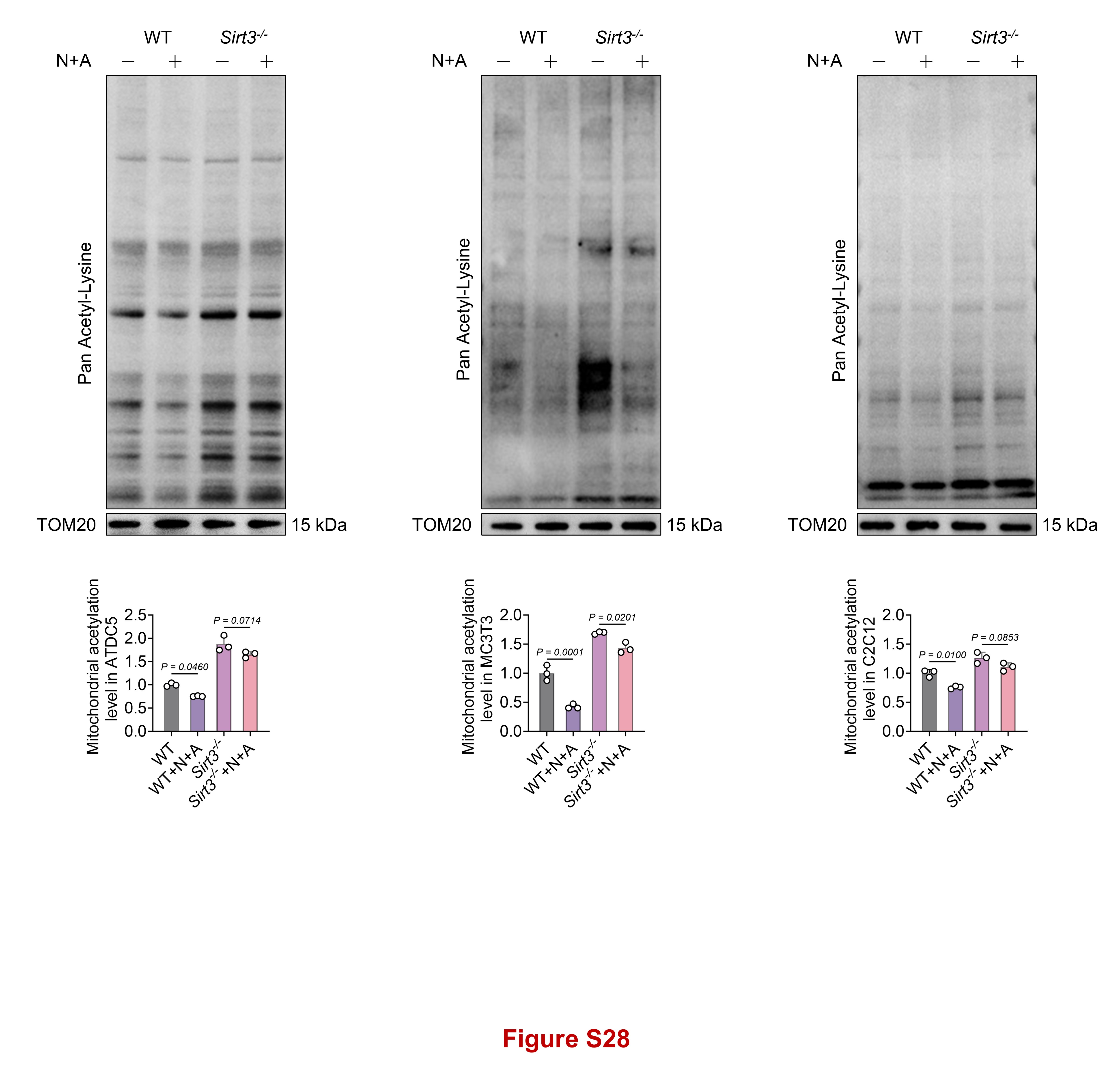


**Figure S28.** Effects of SIRT3 deficiency on the acetylation level of mitochondrial proteins in N+A-treated cells and its quantitative analysis Data are presented as mean ± SD and statistical significance is determined by one-way ANOVA. Statistically significant differences between the indicated groups are denoted by (*P* < 0.05).


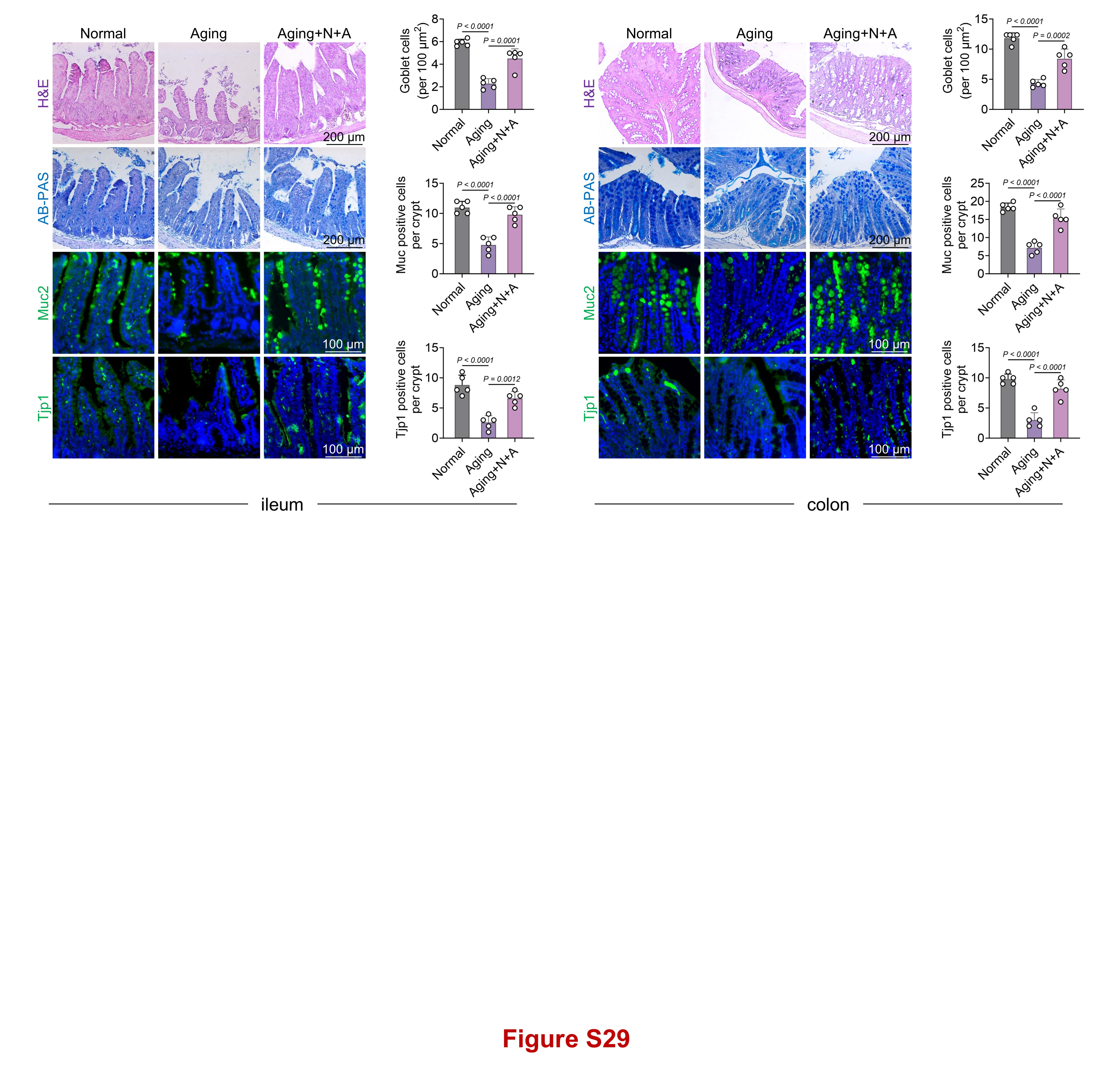


**Figure S29.** Representative images and quantitative analysis of H&E staining, Alcian Blue Periodic Acid Schiff (AB-PAS) Stain Kit staining, and Muc2/Tjp1 immunofluorescence staining in the small intestine and colon structures (n = 5). Statistical significance was determined using one-way ANOVA, with significant differences between groups indicated by *P* < 0.05.


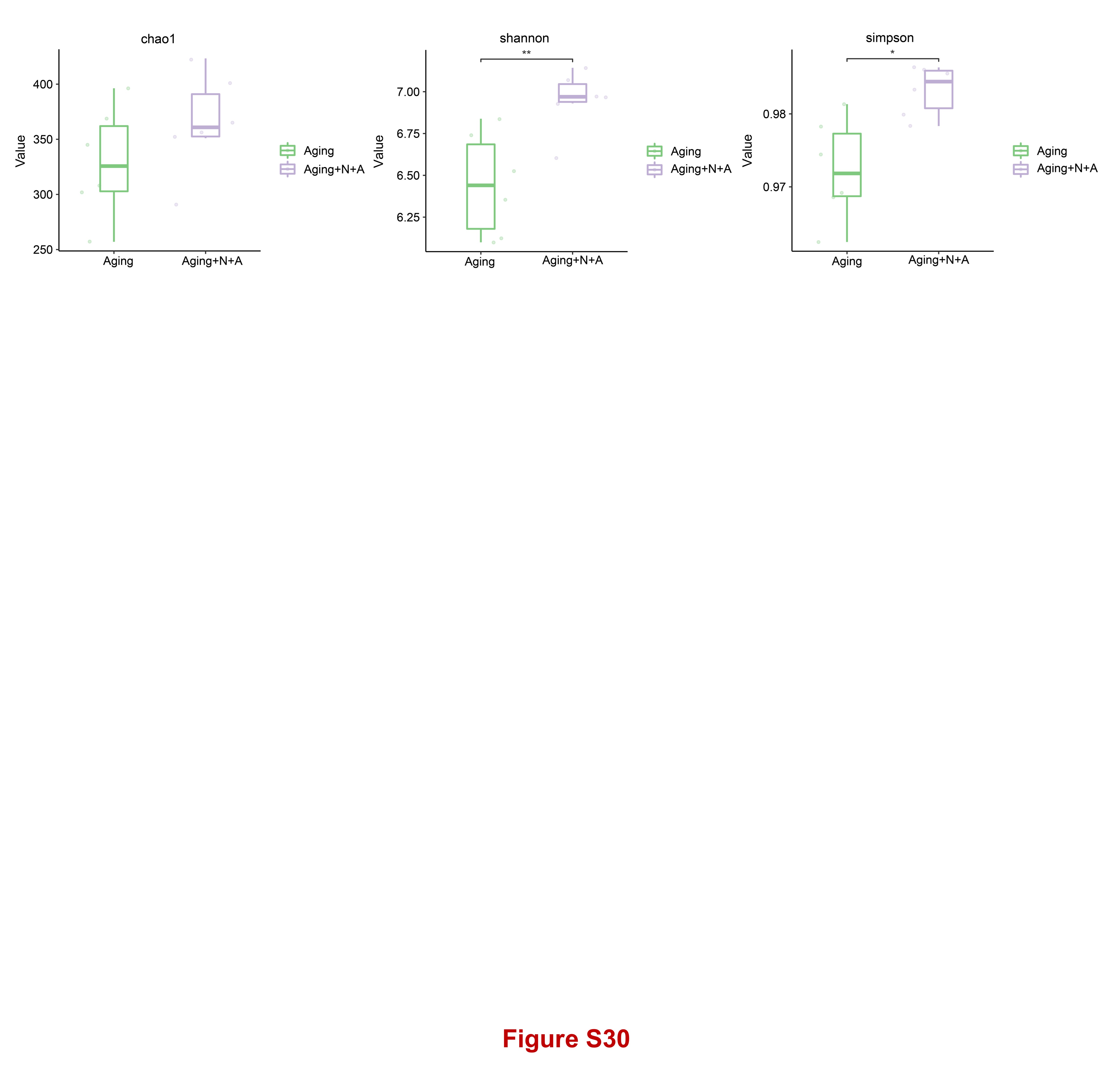


**Figure S30.** Differences in microbial community diversity, as indicated by Chao1 (species richness), Shannon (comprehensive diversity), and Simpson (dominance-related diversity), were observed between the two groups.

**
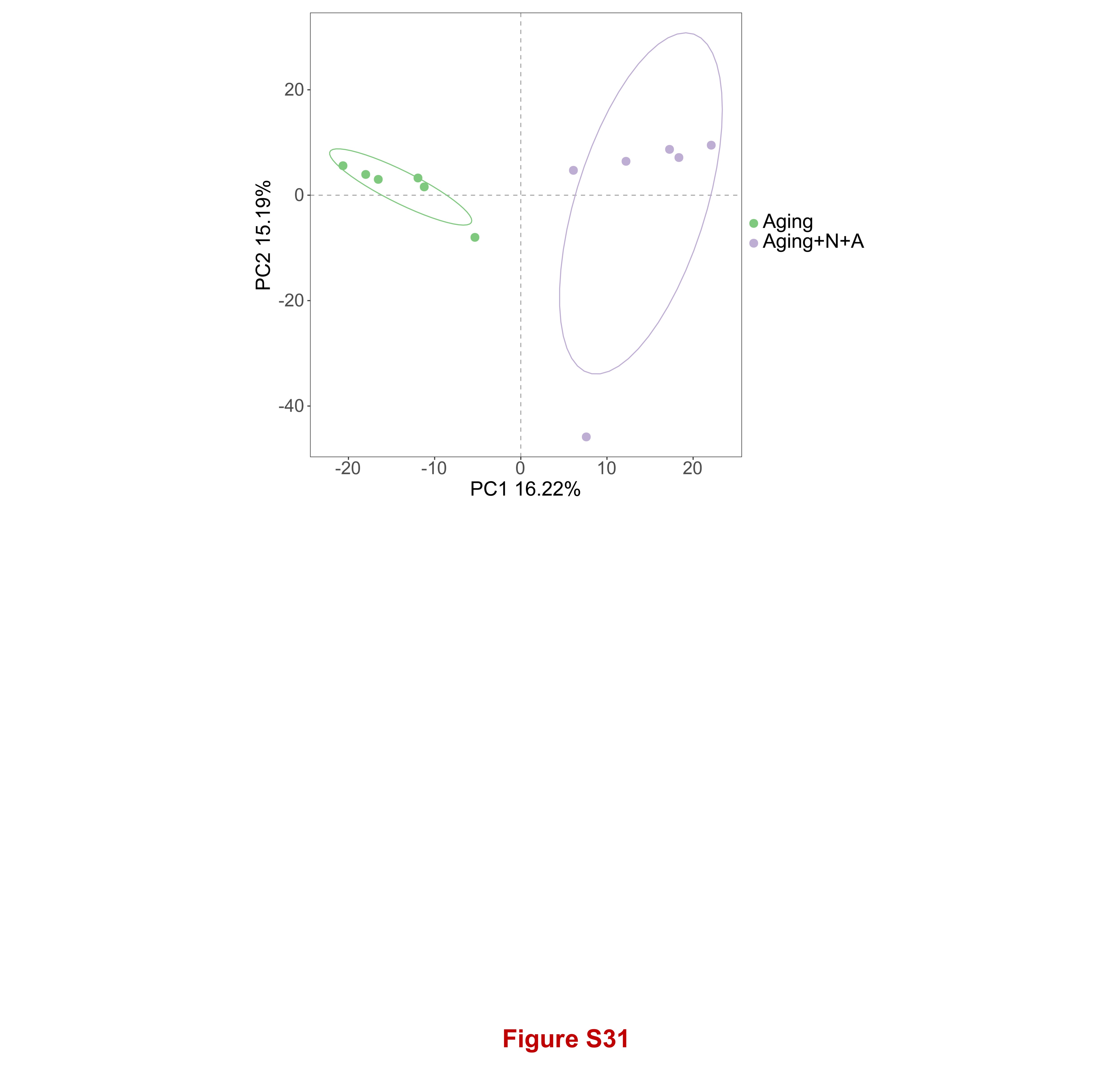
**

**Figure S31.** PCA analysis was conducted to examine the differences in microbial community structure between the two groups.


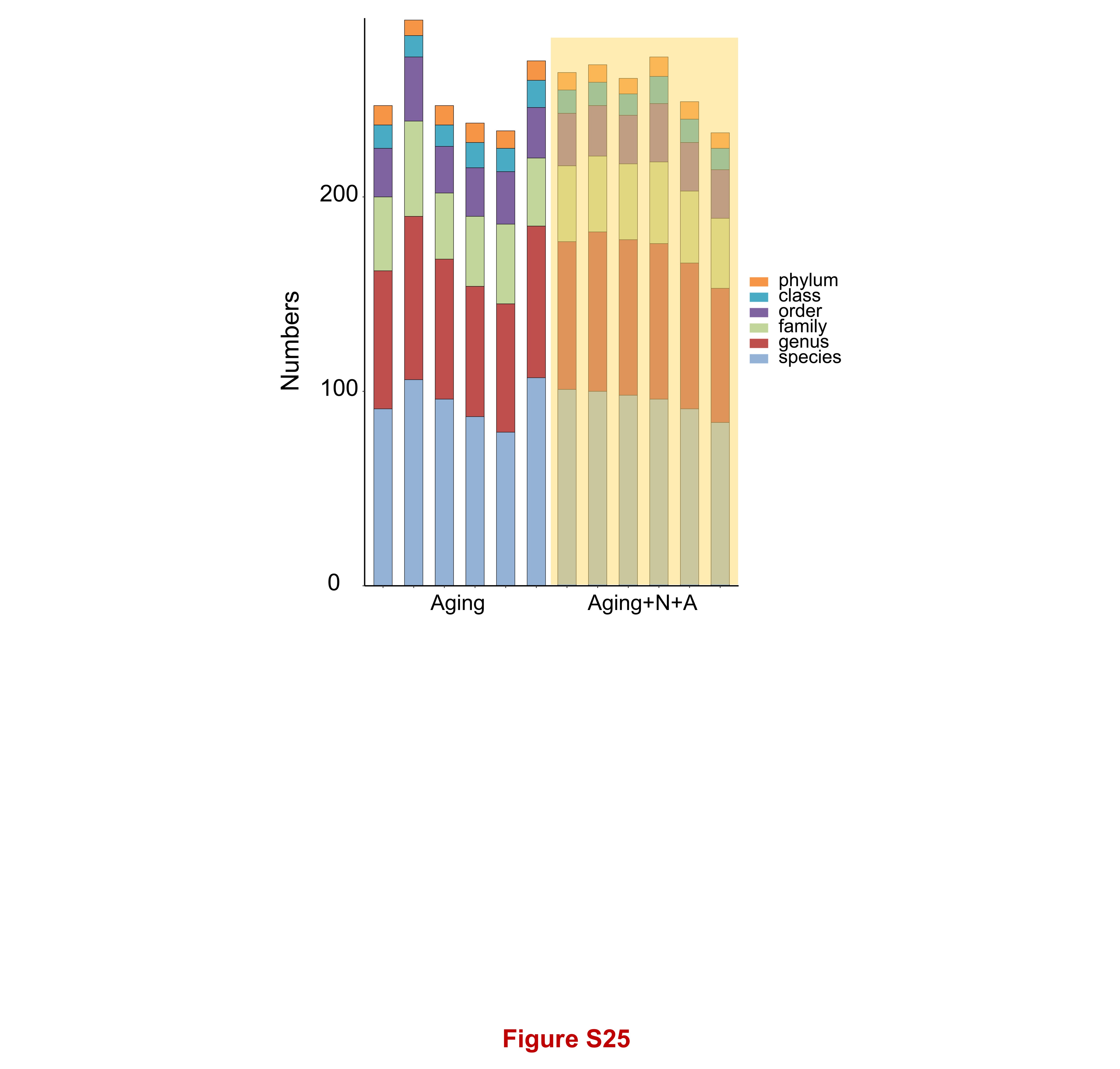


**Figure S32.** The distribution of microbial taxa across taxonomic ranks, including species, genus, family, order, class, and phylum.


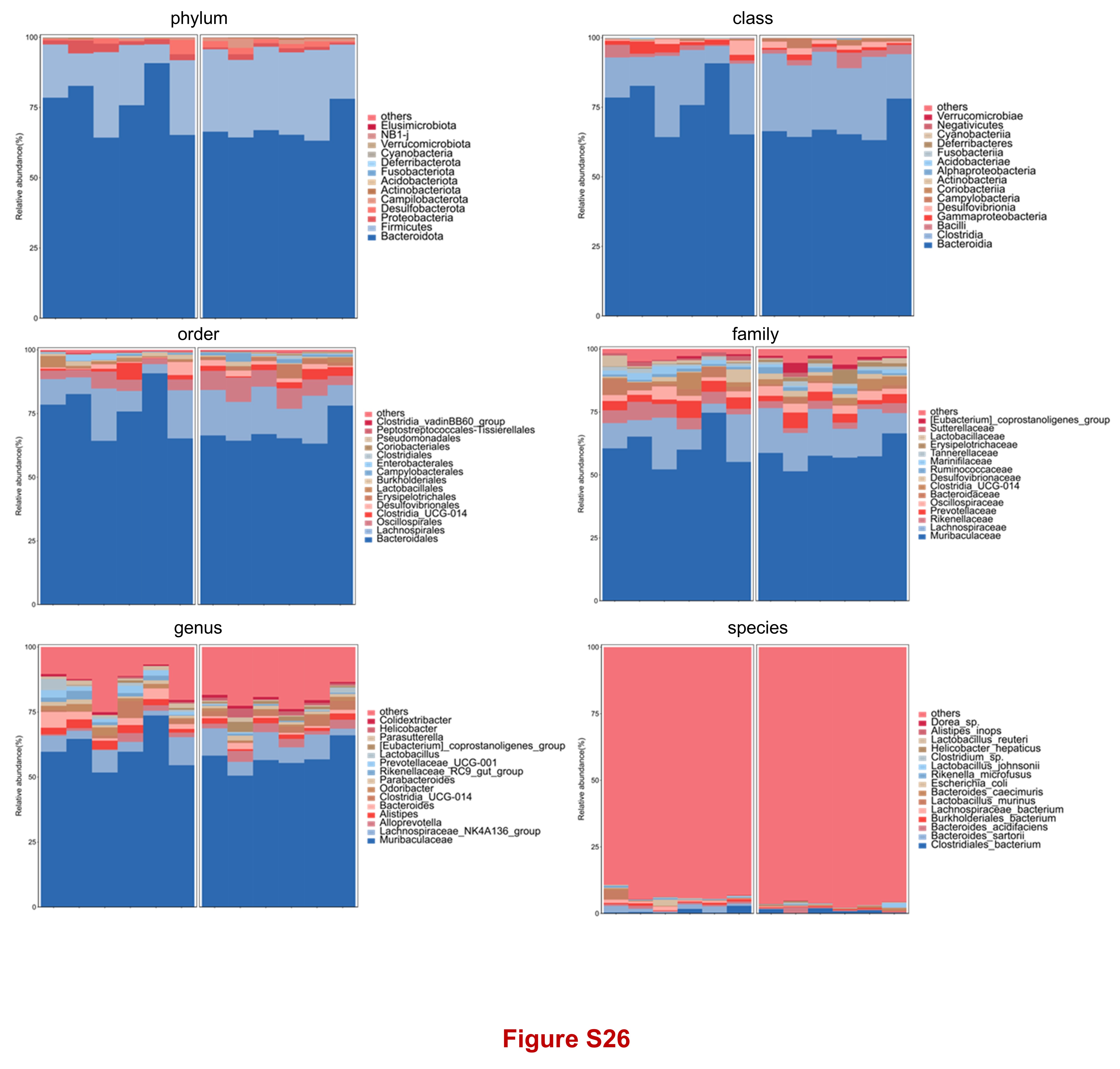


**Figure S33.** The relative abundance distribution of microbial communities was analyzed at various taxonomic levels, including phylum, class, order, family, genus, and species, across samples from the two groups.


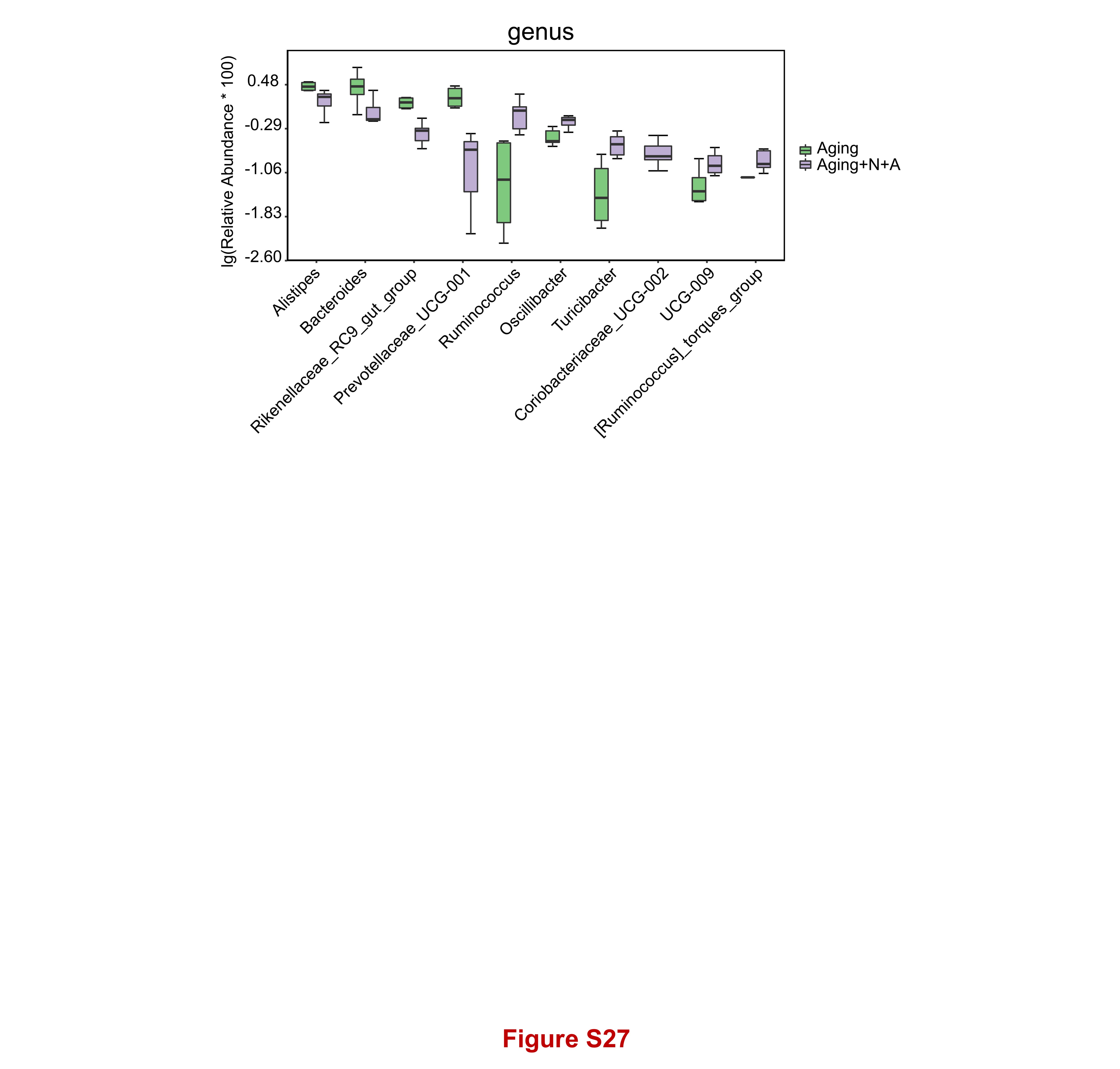


**Figure S34.** Boxplot depicting the relative abundance of microorganisms at the genus level between the two groups.

**
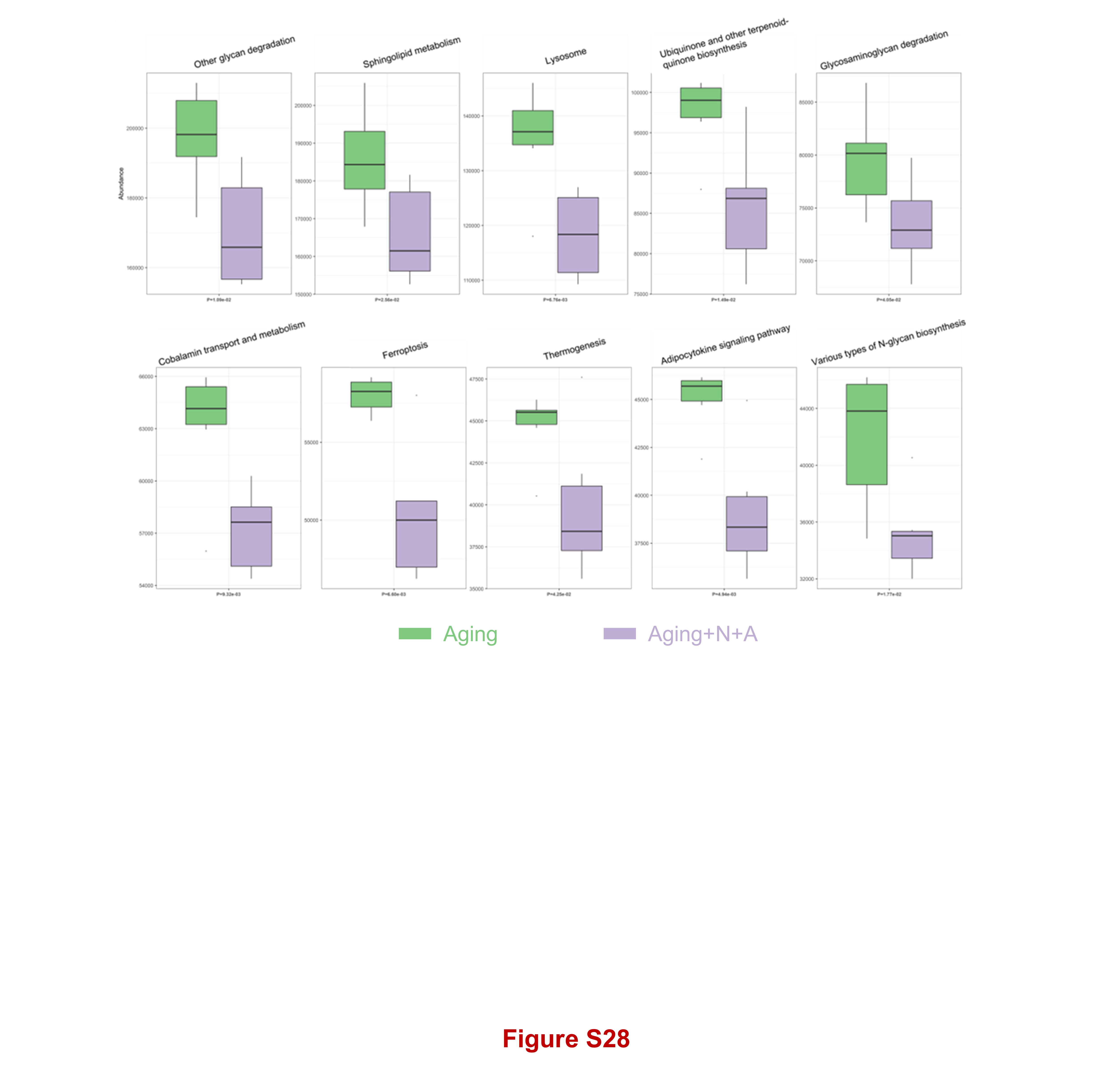
**

**Figure S35.** Abundance differences in 10 metabolic pathways between the two groups.


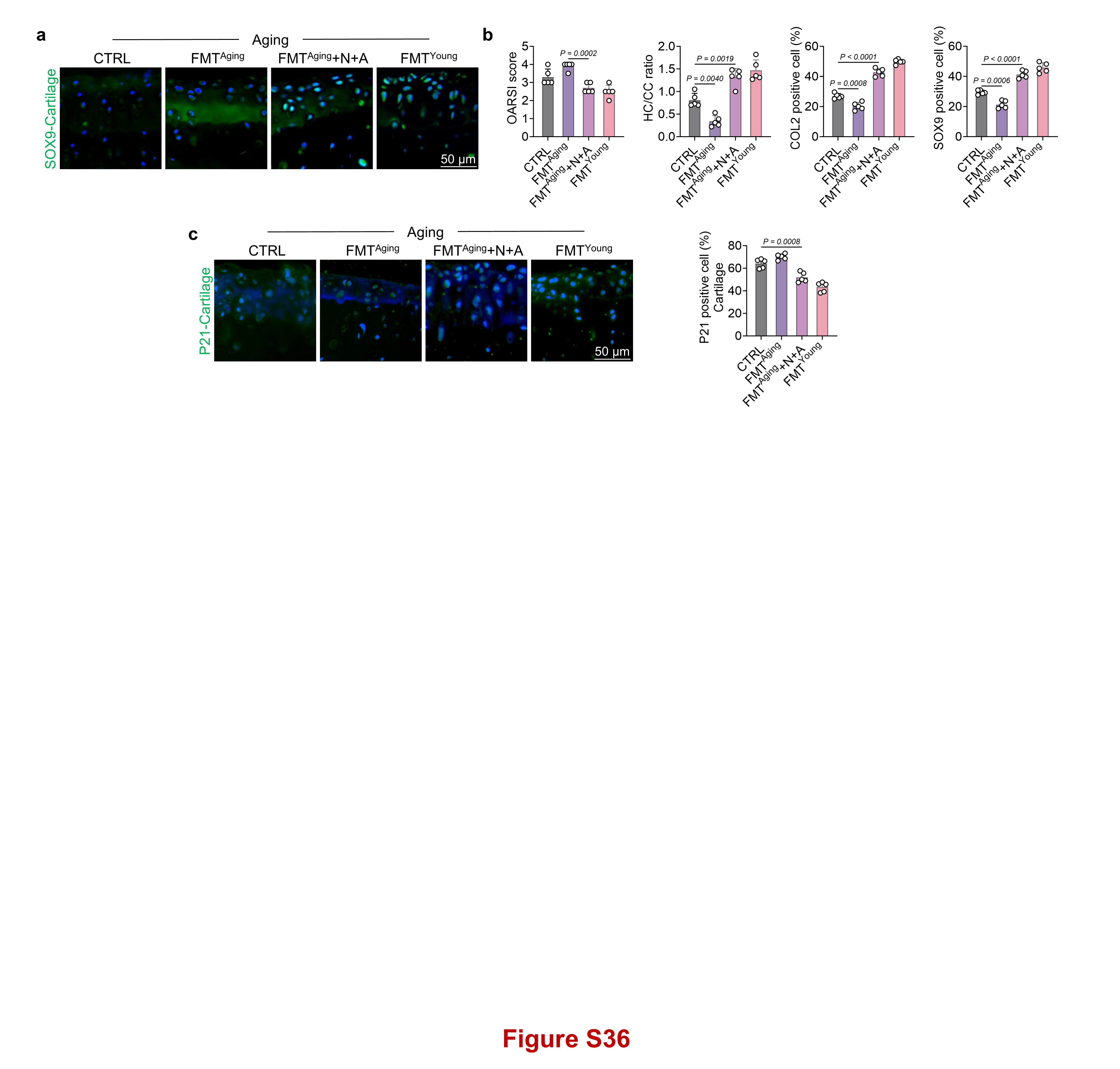


**Figure S36.** FMT attenuates cartilage aging in aged mice and enhances extracellular matrix synthesis. (a) Representative images of SOX9 immunofluorescence staining (n = 5). (b) Quantitative analysis of OARSI scores, HC/CC ratios, and the percentage of COL2- and SOX9-positive cells based on immunofluorescence staining (n = 5). (c) Representative images and quantification of P21 immunofluorescence staining (n = 5). Statistical significance was determined using one-way ANOVA, with significant differences between groups indicated by *P* < 0.05.

**
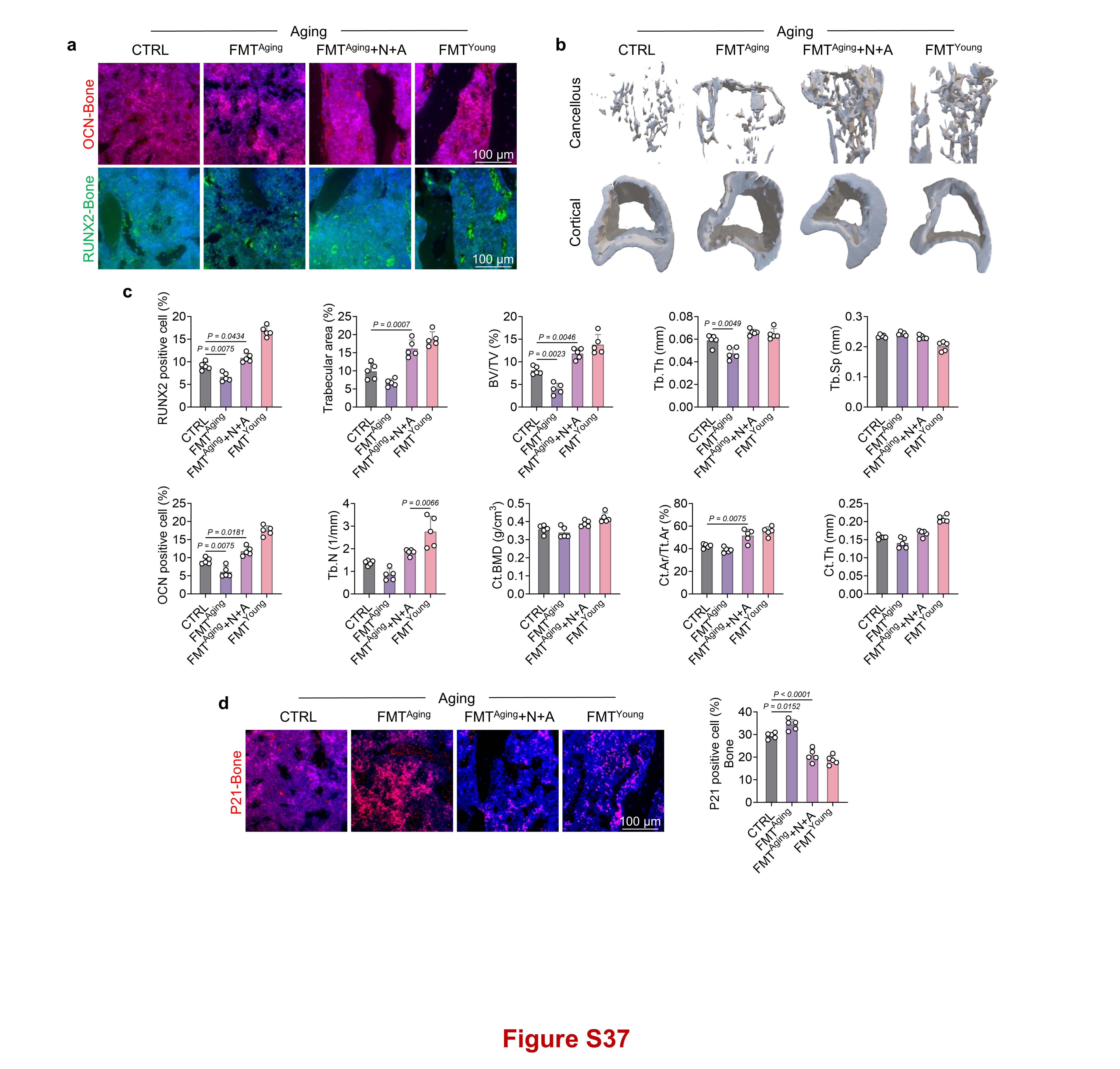
**

**Figure S37.** FMT improves bone aging in aged mice and promotes osteogenesis. (a) Representative images and quantitative analysis of OCN and RUNX2 immunofluorescence staining (n = 5). (b) μCT three-dimensional reconstruction of trabecular and cortical bones in the tibia following SIRT3 knockout and pharmacological treatment (n = 5). (c) Quantitative assessment of trabecular bone thickness (Tb.Th), trabecular number (Tb.N), trabecular separation (Tb.Sp), cortical bone mineral density (Ct.BMD), cortical bone area to total area ratio (Ct.Ar/Tt.Ar), and cortical bone thickness (Ct.Th) via μCT analysis, along with quantitative immunofluorescence analysis of OCN and RUNX2 (n = 5). (d) Representative images and quantitative analysis of P21 immunofluorescence staining (n = 5). Statistical significance was determined using one-way ANOVA, with significant differences between groups indicated by *P* < 0.05.


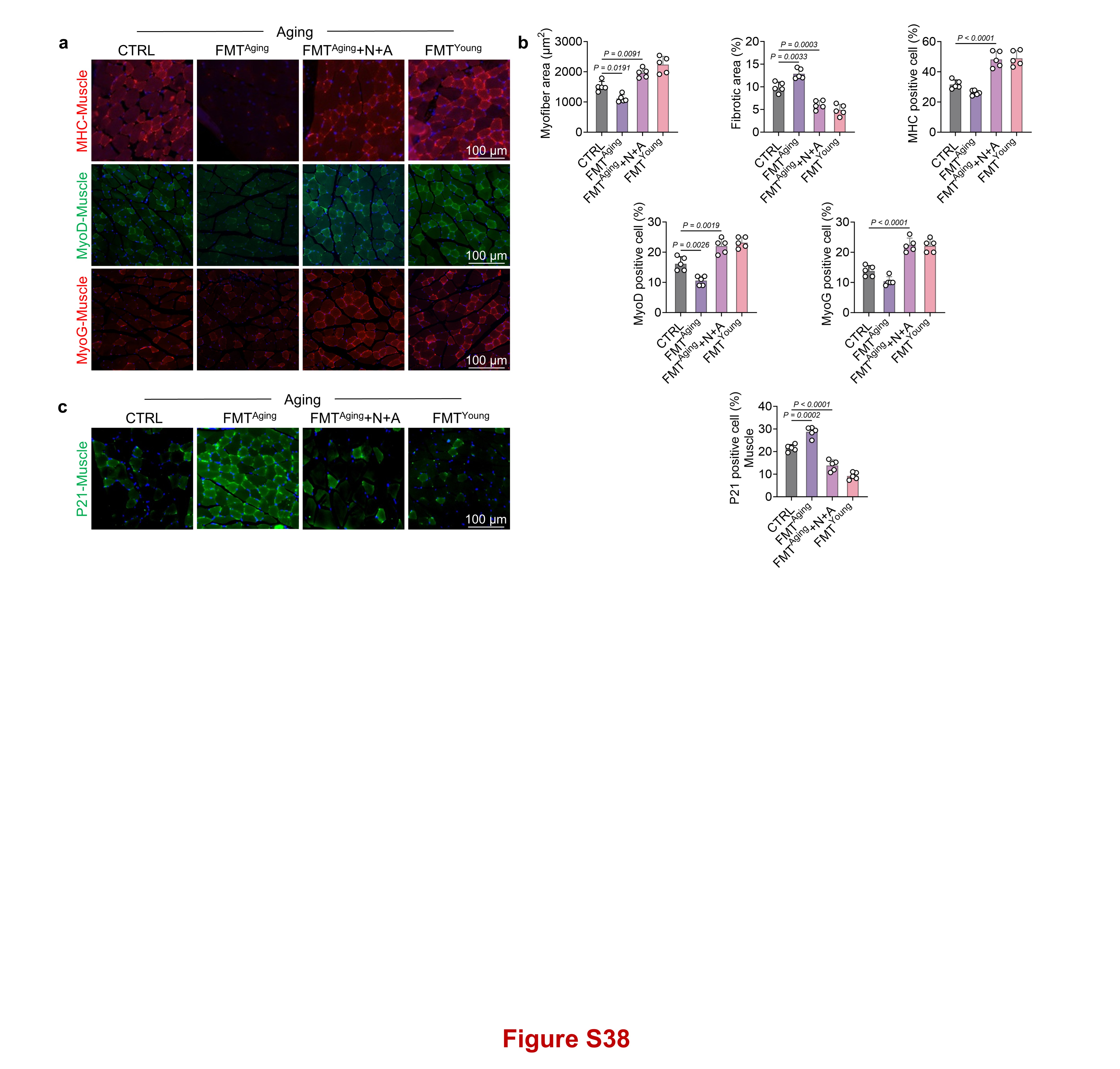


**Figure S38.** FMT mitigates age-related muscle deterioration in aged mice and facilitates myogenesis. (a-c) Representative immunofluorescence images and quantitative analysis of MHC, MyoD, MyoG, and P21 expression in the quadriceps of FMT-treated mice (n = 5). Statistical significance was determined using one-way ANOVA, with significant differences between groups indicated by *P* < 0.05.


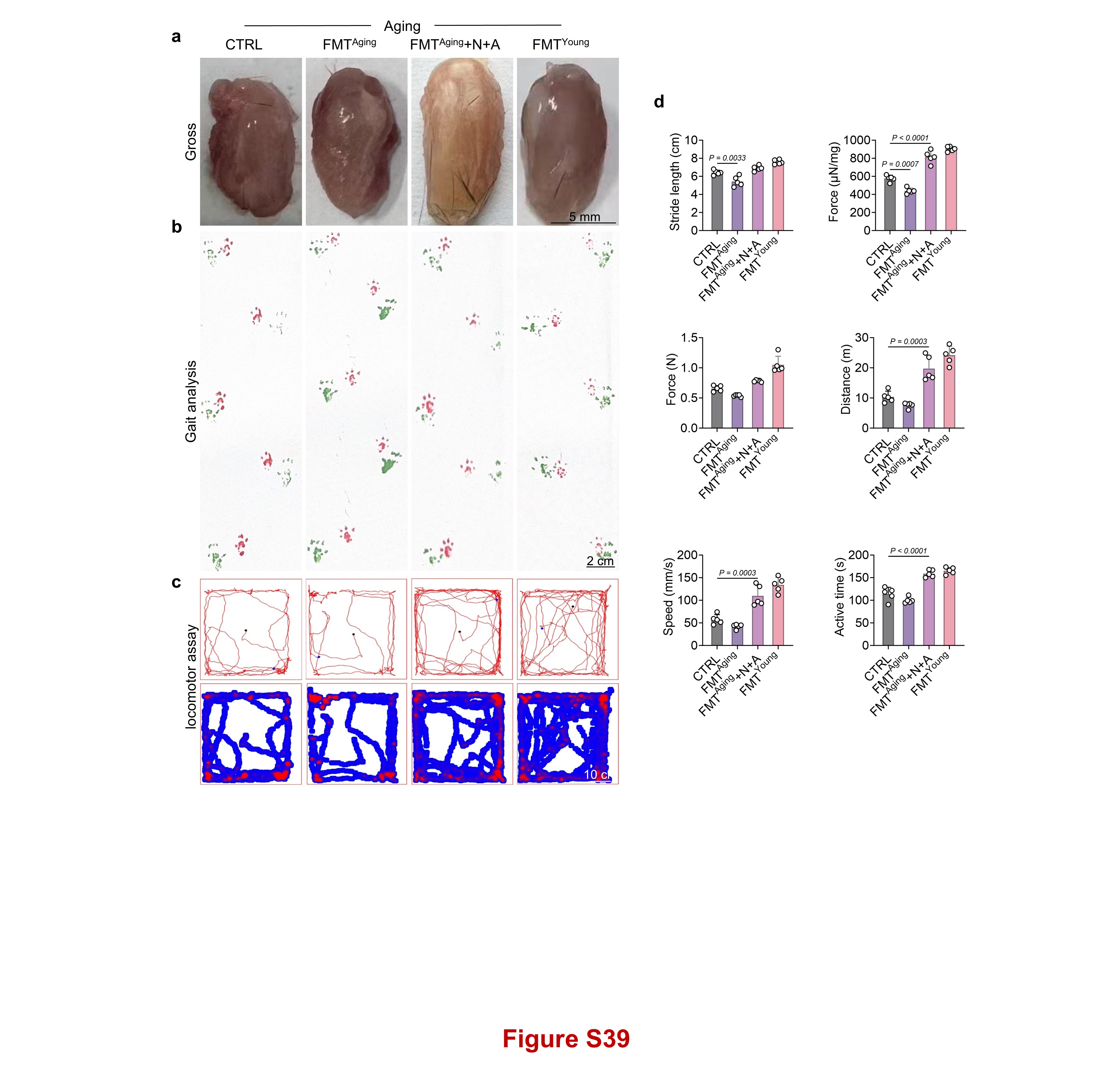


**Figure S39.** FMT enhances the motor function of aged mice. (a) Macroscopic view of the quadriceps femoris in mice. (b) Representative gait analysis images. (c) Representative open field test images. (d) Quantitative assessment of gait parameters, muscle strength, forelimb grip strength, as well as locomotor distance, speed, and activity duration in the open field test in mice (n = 5). Statistical significance was determined using one-way ANOVA, with significant differences between groups indicated by *P* < 0.05.


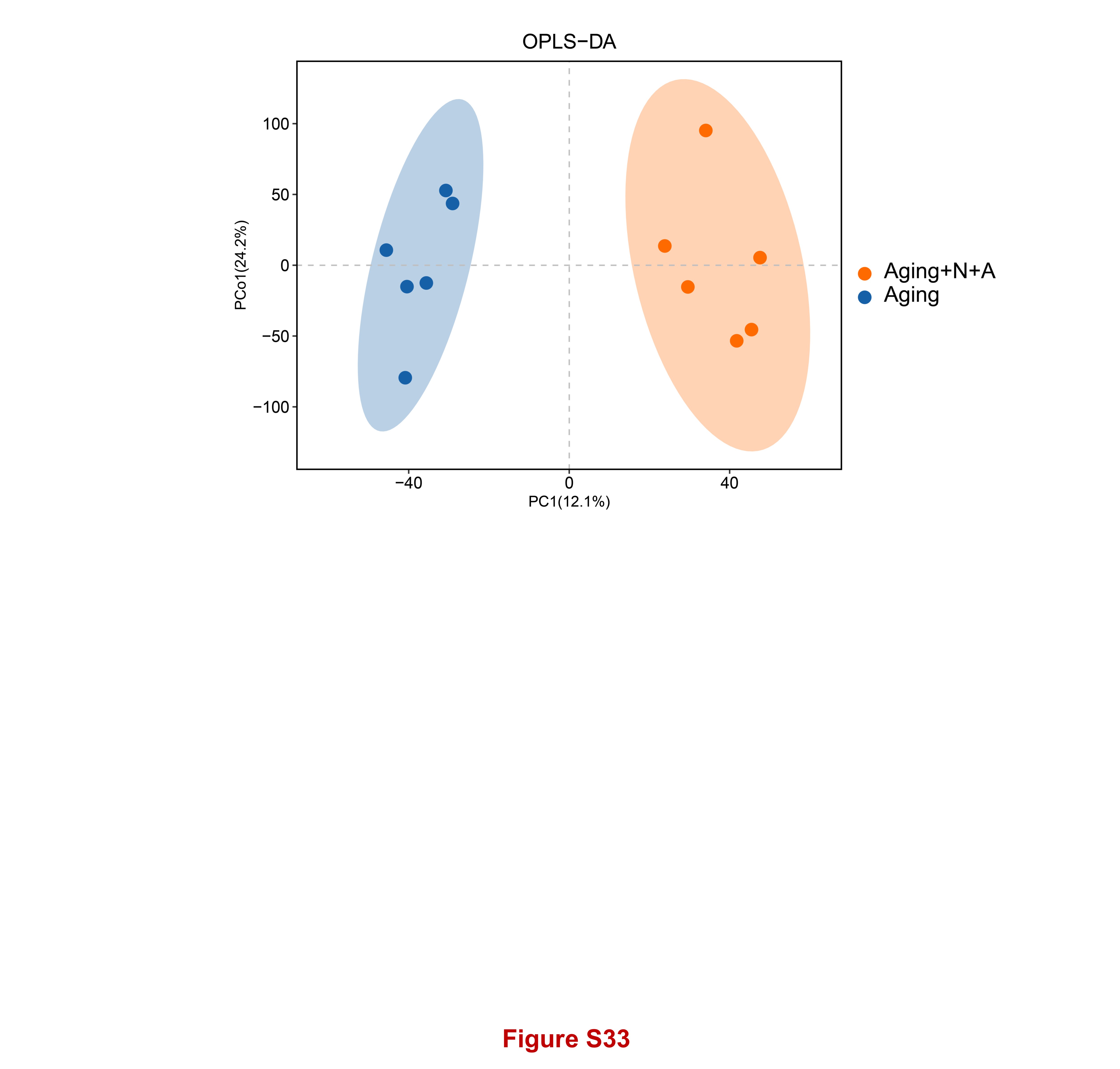


**Figure S40.** PCA analysis was conducted to examine the metabolic differences between the two groups.


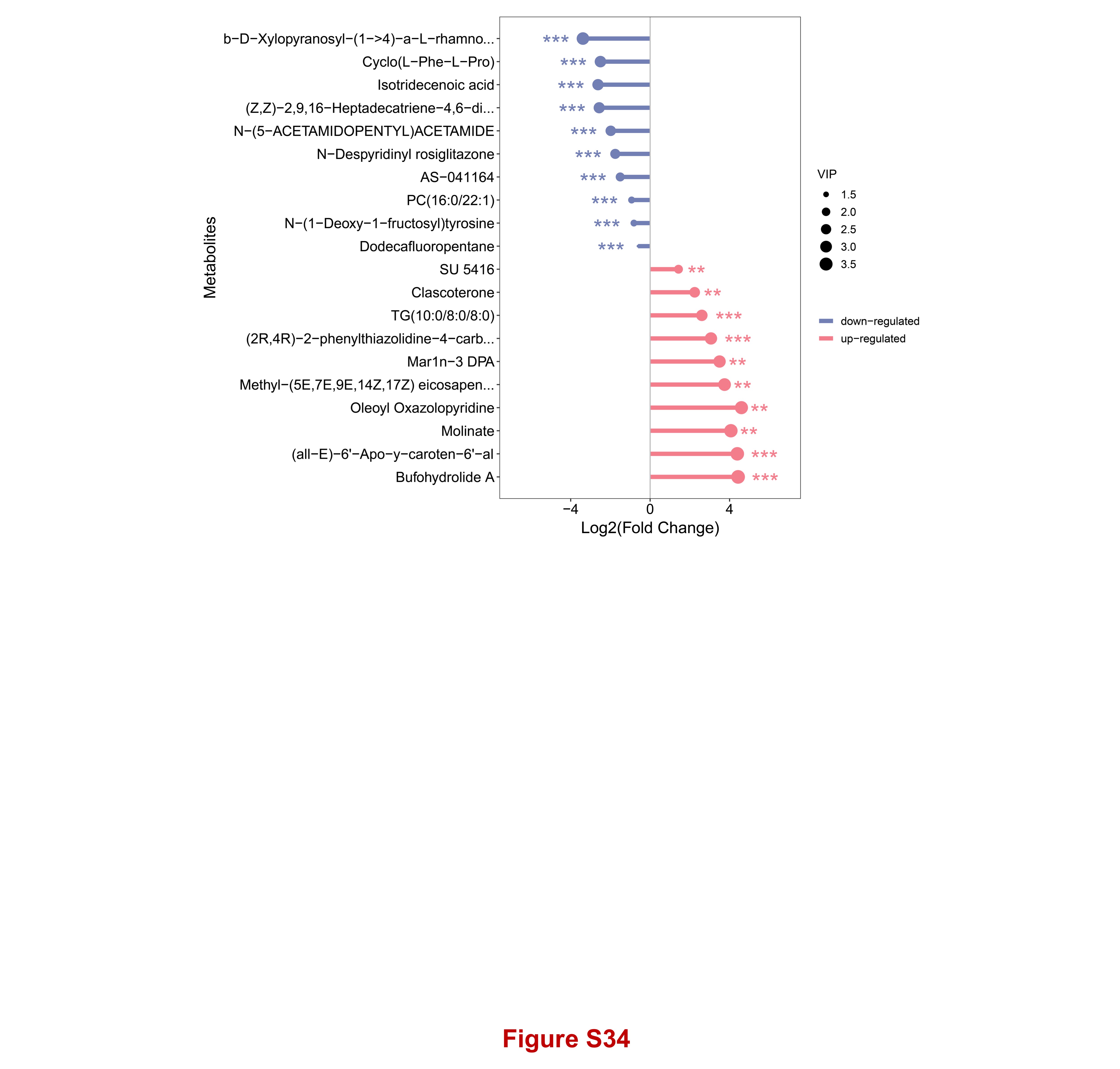


**Figure S41.** The top 10 up-regulated and down-regulated differential metabolites between the two sample groups.


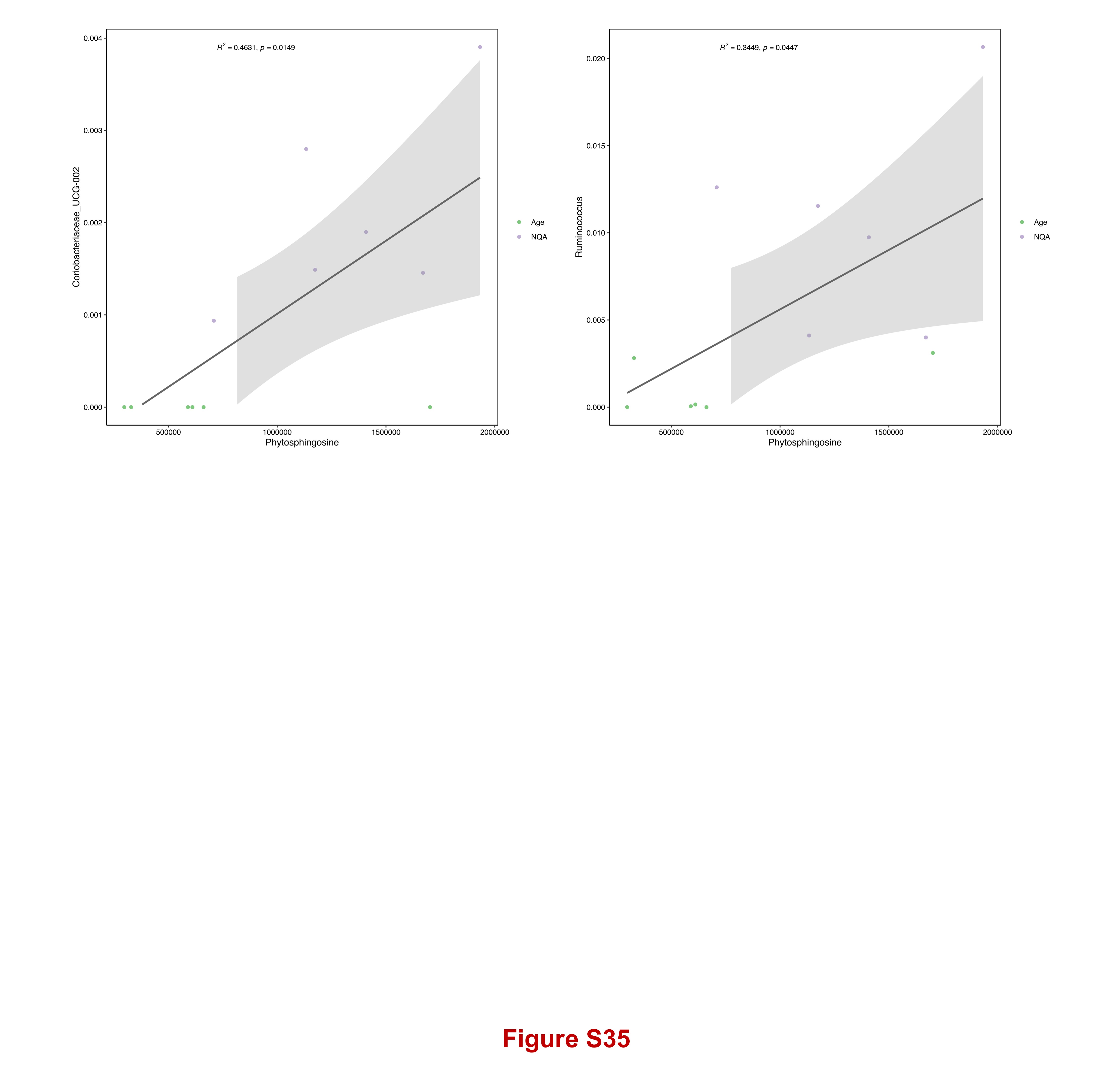


**Figure S42.** The correlation analysis between the metabolite PHS and the gut microbiota taxa *Coriobacteriaceae_UCG-002* and *Ruminococcus* was conducted.


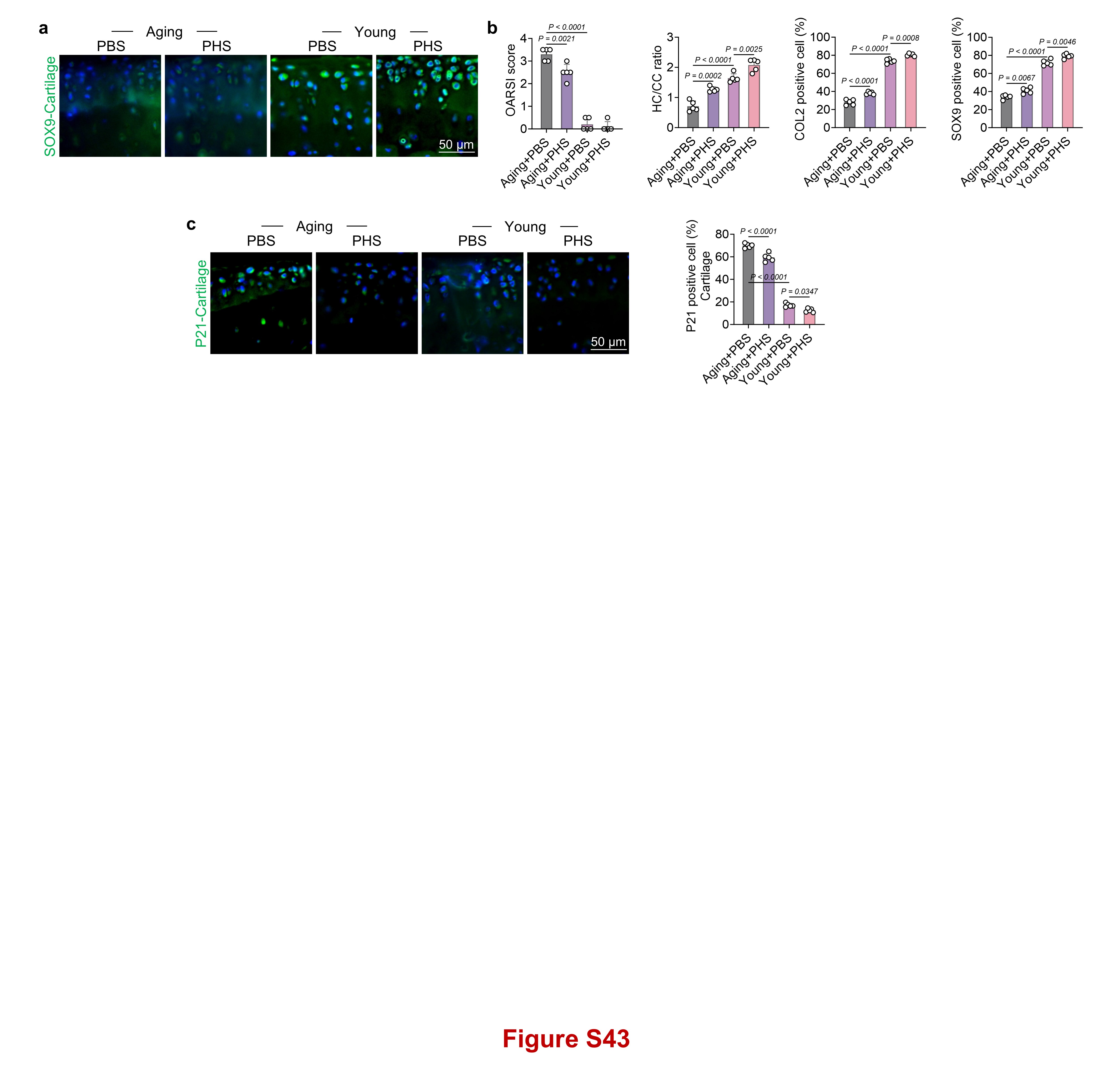


**Figure S43.** PHS attenuates cartilage aging in aged mice and enhances extracellular matrix synthesis. (a) Representative images of SOX9 immunofluorescence staining (n = 5). (b) Quantitative analysis of OARSI scores, HC/CC ratios, and the percentage of COL2- and SOX9-positive cells based on immunofluorescence staining (n = 5). (c) Representative images and quantification of P21 immunofluorescence staining (n = 5). Statistical significance was determined using one-way ANOVA, with significant differences between groups indicated by *P* < 0.05.


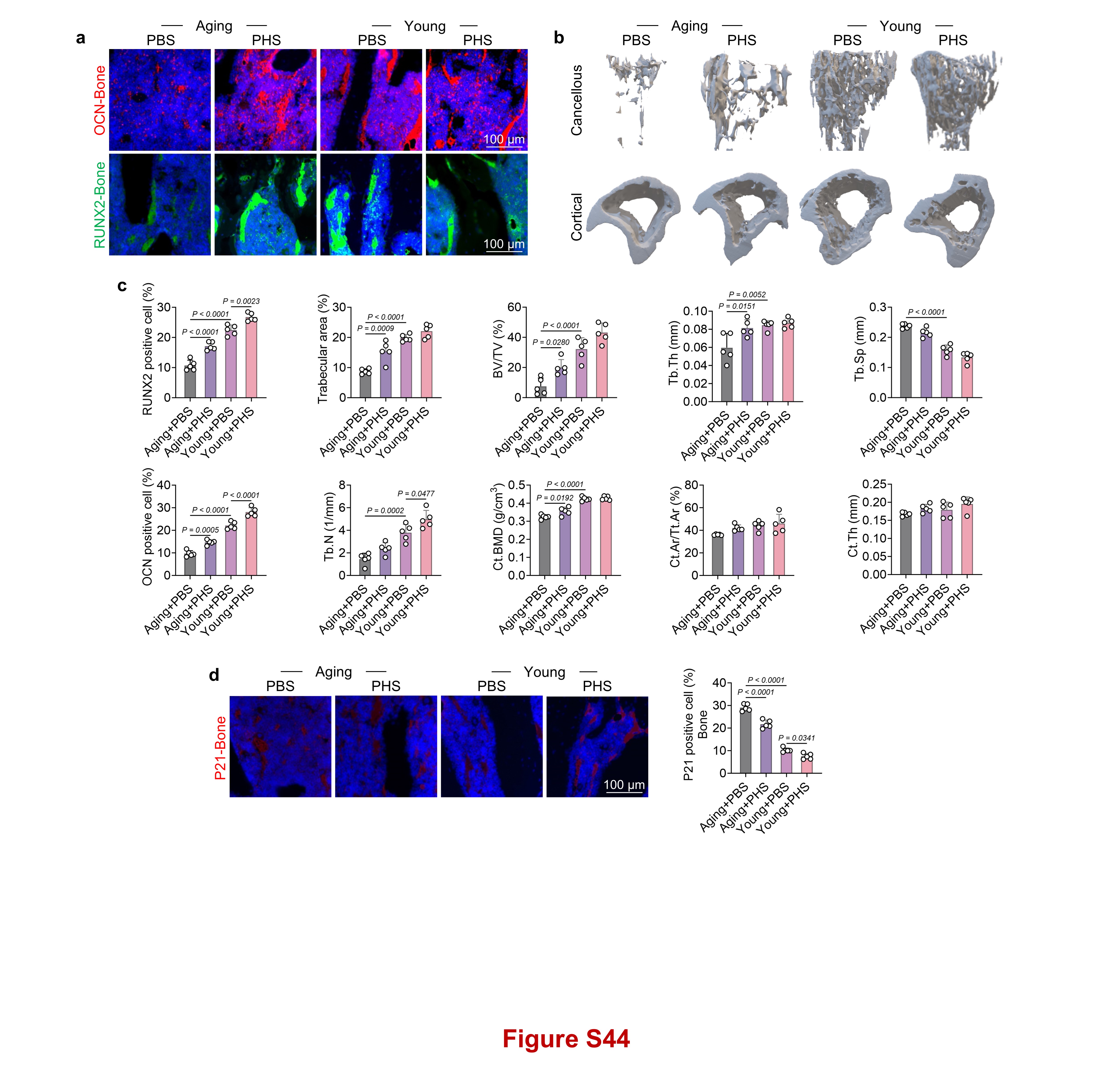


**Figure S44.** PHS improves bone aging in aged mice and promotes osteogenesis. (a) Representative images and quantitative analysis of OCN and RUNX2 immunofluorescence staining (n = 5). (b) μCT three-dimensional reconstruction of trabecular and cortical bones in the tibia following SIRT3 knockout and pharmacological treatment (n = 5). (c) Quantitative assessment of trabecular bone thickness (Tb.Th), trabecular number (Tb.N), trabecular separation (Tb.Sp), cortical bone mineral density (Ct.BMD), cortical bone area to total area ratio (Ct.Ar/Tt.Ar), and cortical bone thickness (Ct.Th) via μCT analysis, along with quantitative immunofluorescence analysis of OCN and RUNX2 (n = 5). (d) Representative images and quantitative analysis of P21 immunofluorescence staining (n = 5). Statistical significance was determined using one-way ANOVA, with significant differences between groups indicated by *P* < 0.05.


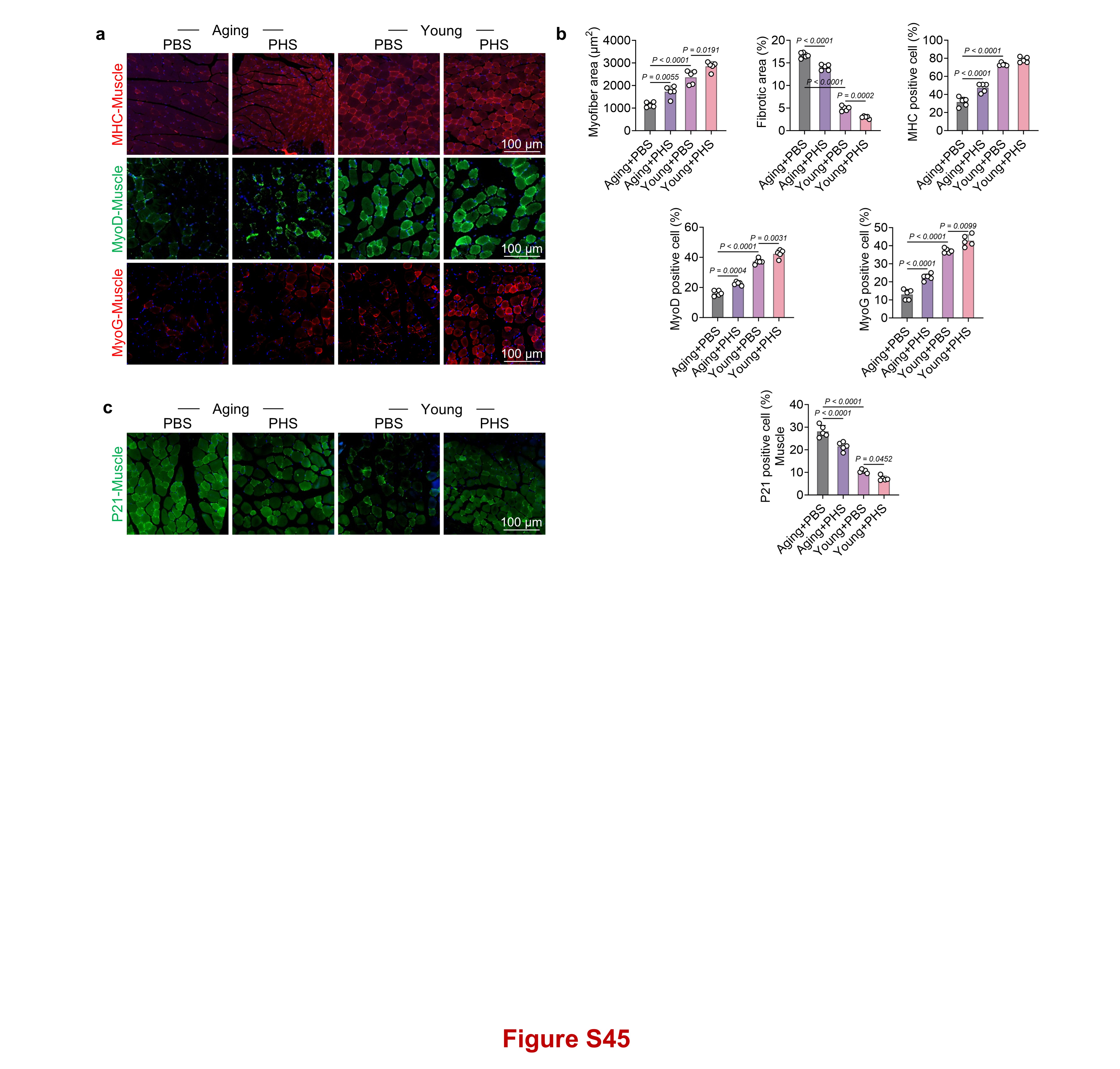


**Figure S45.** PHS mitigates age-related muscle deterioration in aged mice and facilitates myogenesis. (a-c) Representative immunofluorescence images and quantitative analysis of MHC, MyoD, MyoG, and P21 expression in the quadriceps of FMT-treated mice (n = 5). Statistical significance was determined using one-way ANOVA, with significant differences between groups indicated by *P* < 0.05.


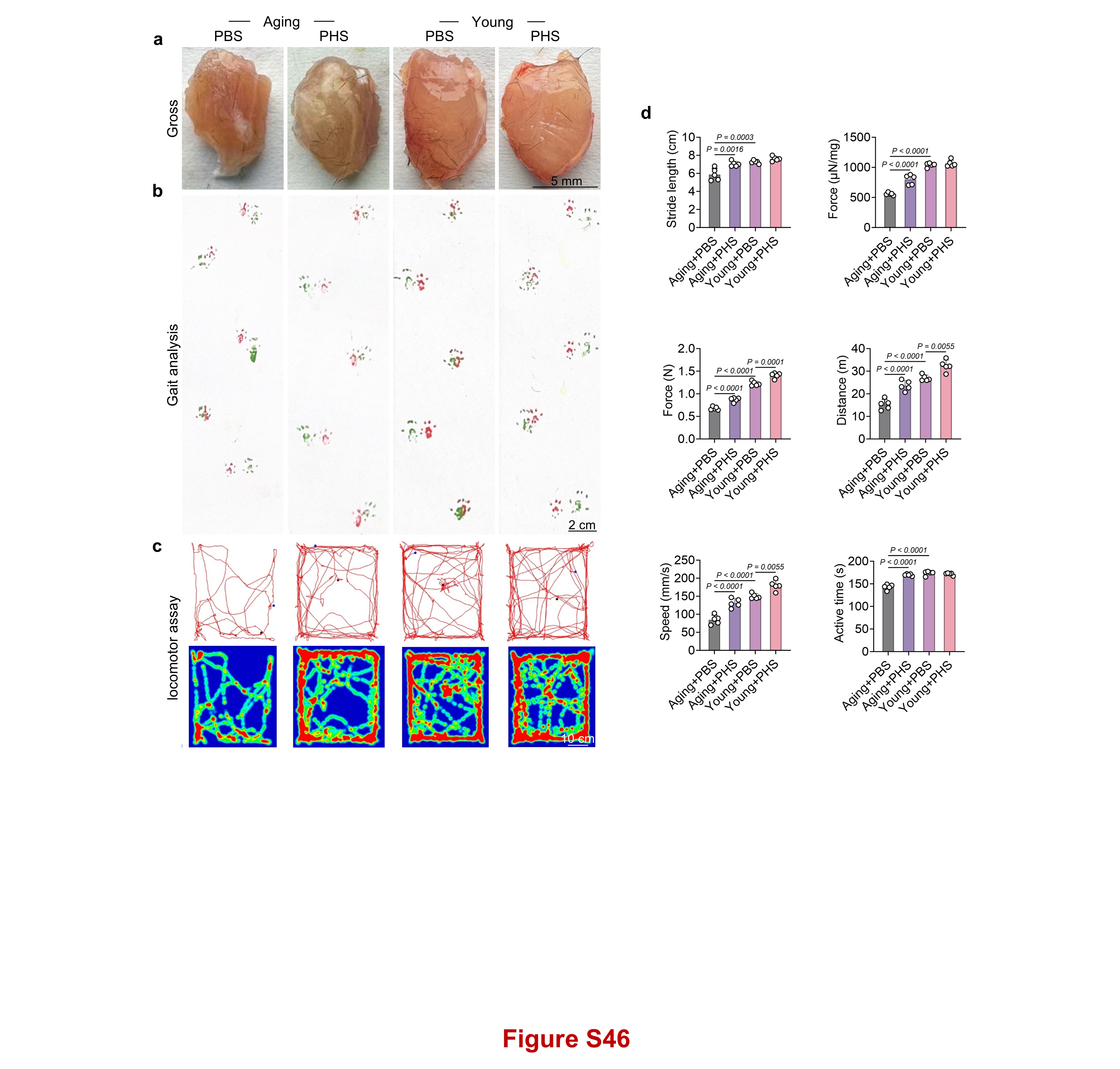


**Figure S46.** PHS enhances the motor function of aged mice. (a) Macroscopic view of the quadriceps femoris in mice. (b) Representative gait analysis images. (c) Representative open field test images. (d) Quantitative assessment of gait parameters, muscle strength, forelimb grip strength, as well as locomotor distance, speed, and activity duration in the open field test in mice (n = 5). Statistical significance was determined using one-way ANOVA, with significant differences between groups indicated by *P* < 0.05.

**Supplementary Tables**

**Supplementary Table 1.** Primers used for qRT-PCR assay

| ***Mmu*** |  | **Forward Primer sequence (5'-3')** | | **Reverse Primer sequence (5'-3')** | |  |
| --- | --- | --- | --- | --- | --- | --- |
| *Gapdh* |  | AGGTCGGTGTGAACGGATTTG | | TGTAGACCATGTAGTTGAGGTCA | |  |
| *Myod1* |  | ACTTCTATGATGACCCGTGTTT | | ACATGCTCATCCTCACGAG | |  |
| *Myog* |  | AACCCAGGAGATCATTTGCTC | | GAAGGCAACAGACATATCCTCC | |  |
| *Myhc7* |  | CTACCAGACAGAGGAAGACAAG | | TAGGCCTTCACCTTCAACTGTA | |  |
| *Col2a1* |  | TACTGGAGTGACTGGTCCTAAG | | AACACCTTTGGGACCATCTTTT | |  |
| *Sox9* |  | GAGTTTGACCAATACTTGCCAC | | GTAACTGCCAGTGTAGGTGAC | |  |
| *Runx2* |  | CCTTCAAGGTTGTAGCCCTC | | GGAGTAGTTCTCATCATTCCCG | |  |
| *Bglap* |  | GGACCATCTTTCTGCTCACTCTGC | | TCCTGCTTGGACATGAAGGCTTTG | |  |
| *Sp7* |  | TCGTCTGACTGCCTGCCTAGTG | | CTGCGTGGATGCCTGCCTTG | |  |
| *Tp53* |  | | TGGAAGGAAATTTGTATCCCGA | | GTGGATGGTGGTATACTCAGAG | |
| *Cdkn1a* |  | ATGTCCAATCCTGGTGATGTC | | GAAGTCAAAGTTCCACCGTTC | |  |
| *Cdkn2a* |  | TCAAGACATCGTGCGATATTTG | | TTAGCTCTGCTCTTGGGATTG | |  |
| *Sirt3* |  | TCTATACACAGAACATCGACGG | | GCATGTAGCTGTTACAAAGGTC | |  |
| *Tnf* |  | ATGTCTCAGCCTCTTCTCATTC | | GCTTGTCACTCGAATTTTGAGA | |  |
| *Mmp3* |  | TGTCACTGGTACCAACCTATTC | | TCTCAGGTTCCAGAGAGTTAGA | |  |
| *H2ax* |  | CTTCAGTTCCCTGTAGGCC | | GATCTCGGCAGTGAGGTAC | |  |
| *Cxcl8* |  | CTGTTGGCCCAATTACTAACAG | | TCCCGAATTGGAAAGGGAAATA | |  |
| *Il6* |  | CTCCCAACAGACCTGTCTATAC | | CCATTGCACAACTCTTTTCTCA | |  |

*Mmu: Mus musculus*

**KEY RESOURCES TABLE**

| REAGENT | SOURCE | IDENTIFIER |  |
| --- | --- | --- | --- |
| **Antibodies** | | |  |
| SOX9 Rabbit Monoclonal antibody | ABclonal | Cat# A19710; RRID: AB_2862748 |  |
| P21 Rabbit recombinant Monoclonal antibody | ABclonal | Cat# A22460PM; RRID: AB_3713033 |  |
| P53 Rabbit monoclonal antibody | ABclonal | Cat# A25915; RRID: AB_3674525 |  |
| Anti-Myosin Mouse monoclonal antibody | Abcam | Cat# ab37484; RRID: AB_2921304 |  |
| P16 Rabbit Polyclonal antibody | ABclonal | Cat# A11651; RRID: AB_2861619 |  |
| COL2 Rabbit Polyclonal antibody | ABclonal | Cat# A1560; RRID: AB_2763005 |  |
| MYOG Rabbit Polyclonal antibody | Affinity | Cat# DF8273; RRID: AB_2841562 |  |
| MyoD1 Rabbit Polyclonal antibody | Affinity | Cat# AF7733; RRID:AB_2844097 |  |
| Osteocalcin Rabbit Polyclonal antibody | ABclonal | Cat#A6205; RRID: AB_2766815 |  |
| Osteoprotegerin Rabbit Polyclonal antibody | Affinity | Cat# DF6824; RRID: AB_2838784 |  |
| RUNX2 Rabbit Polyclonal antibody | Affinity | Cat# AF5186; RRID: AB_2837672 |  |
| Goat Anti-Rabbit IgG (H+L) Fluor488-conjugated | Affinity | Cat#S0018; RRID: AB_2846215 |  |
| Goat Anti-Rabbit IgG (H+L) CY3-conjugated | Affinity | Cat#S0011; RRID: AB_2844800 |  |
| Goat Anti-Mouse IgG (H+L) CY3-conjugated | Affinity | Cat#S0012 RRID: AB_2844799 |  |
| Goat Anti-Mouse IgG (H+L) FITC-conjugated | ABclonal | Cat#AS001RRID: AB_2769475 |  |
| Goat Anti-Rat IgG (H+L) FITC-conjugated | ABclonal | Cat#AS019; RRID: AB_2769477 |  |
| ZO-1 Rabbit Polyclonal antibody | ABclonal | Cat# A11417; RRID: AB_2758549 |  |
| MUC2 Rabbit Polyclonal antibody | ABclonal | Cat# A14659; RRID: AB_2761535 |  |
| Polyclonal Rabbit Anti-beta actin antibody | Servicebio | Cat#GB11001-100; RRID: AB_2801259 |  |
| Goat Anti- Mouse IgG (H+L) HRP | Affinity | Cat#S0002 RRID: AB_2839430 |  |
| Goat Anti-Rabbit IgG (H+L) HRP | Affinity | Cat#S0001; RRID: AB_2839429 |  |
| TOM20 Rabbit Monoclonal antibody | ABclonal | Cat#A19403; RRID: AB_2862646 |  |
| CD38 Rabbit Polyclonal antibody | ABclonal | Cat#A1680; RRID: AB_2763735 |  |
| Pan Acetylation Mouse Monoclonal antibody | Proteintech | Cat#66289-1-1g; RRID: AB_2881672 |  |
| **Chemicals, Peptides, and Recombinant Proteins** | | |  |
| TGF-β3 | Novoprotein | Cat# CJ44 |  |
| β-Glycerophosphate disodium salt hydrate | Sigma-Aldrich | Cat#G9422 |  |
| Dexamethasone | Sigma-Aldrich | Cat#D4902 |  |
| Ascorbic acid | Sigma-Aldrich | Cat# PHR1008 |  |
| Apigenin | MACKLIN | Cat# A800500 |  |
| Beta-Nicotinamide Mononucleotide | MACKLIN | Cat# B832936 |  |
| ITS-G | Absin | Cat# abs9462 |  |
| Horse Serum | Biosharp | Cat#BL209A |  |
| Bacitracin | MACKLIN | Cat#B802311 |  |
| Ampicillin | MACKLIN | Cat# A830931 |  |
| Vancomycin | MACKLIN | Cat# V871983 |  |
| Neomycin sulfate | MACKLIN | Cat# N6063 |  |
| Meropenem trihydrate | MACKLIN | Cat# M843917 |  |
| Phytosphingosine | MACKLIN | Cat#P838474 |  |
| Type II Collagenase | Sigma-Aldrich | Cat# C2-BIOC |  |
| MEMα | Procell | Cat# PM150421 |  |
| PBS | Procell | Cat# PB180327 |  |
| DMEM (High Glucose) | Keygen Bio ECH | Cat# KGL1206-500 |  |
| DMEM/F-12 | Keygen Bio ECH | Cat# KGL1201-500 |  |
| Fetal Bovine Serum (FBS) | Vazyme Biotech | Cat# F103 |  |
| Penicillin Streptomycin | Gibco | Cat#15140122 |  |
| Paraformaldehyde (PFA) | Servicebio | Cat#G1101 |  |
| Assay Buffer (RIPA buffer) | NCM Biotech | Cat#WB3100 |  |
| SDS Loading Buffer | Beyotime | Cat#P0015 |  |
| Nitrocellulose membrane | Beyotime | Cat#FFN03 |  |
| Western blocking solution | Beyotime | Cat#P0023B |  |
| Western Wash Buffer | Beyotime | Cat#P0023C3 |  |
| YoungPAGE™ Bis-Tris Precast Gradient Gel (4-12%, 8-well×10 gels) | GenScript | Cat#M00938 |  |
| ultra-sensitive Enhanced Chemiluminescent | NCM Biotech | Cat#P10100 |  |
| TRIzol® reagent | Sigma-Aldrich | Cat# T9424 |  |
| ChamQ Blue Universal SYBR qPCR Master Mix | Vazyme | Cat#Q312-02 |  |
| QuickBlock™ Blocking Buffer | Beyotime | Cat#P0228 |  |
| Triton X-100 | Beyotime | Cat#P0096 |  |
| DAPI | Sigma-Aldrich | Cat# D9542 |  |
| TRITC Phalloidin | Solarbio | Cat# CA1610 |  |
| FITC Phalloidin | Solarbio | Cat#CA1620 |  |
| PKH26 | MedChemExpress | Cat#367265 |  |
| Isoflurane | RWD | Cat#R510-22-10 |  |
| Ethylenedinitrilotetraacetic acid (EDTA) | Sigma-Aldrich | Cat#E9884 |  |
| Safranin O | Sigma-Aldrich | Cat#S2255 |  |
| Fast Green FCF | Sigma-Aldrich | Cat#F7252 |  |
| Hematoxylin and Eosin (H&E) | Jiancheng | Cat#D006 |  |
| 180 kDa Prestained Protein Marker | Vazyme Biotech | Cat# MP 102-01 |  |
| 250 kDa Plus Prestained Protein Marker | Vazyme Biotech | Cat# MP202-01 |  |
| **Critical Commercial Assays** |  |  |  |
| BCA Protein Quantification Kit | Vazyme | Cat#E112-01/02 |  |
| Nuclear and Cytoplasmic Protein Extraction Kit | Beyotime | Cat#P0028 |  |
| Proliferation Marker (JC1) | Beyotime | Cat# C2005 |  |
| DCFH-DA | Medchemexpress | Cat# HY-D0940 |  |
| MitoSOX Red Mitochondrial Superoxide Indicator | Medchemexpress | Cat# HY-D1055 |  |
| Modified Giemsa Staining Solution | Beyotime | Cat# C0131 |  |
| Masson Stain Kit | Nanjing Jiancheng Bioengineering Institute | Cat# D026-1 |  |
| AB-PAS | Biosharp | Cat#BL1123A |  |
| Alcian staining kit | Solarbio | Cat# G1560 |  |
| ALP staining kit | Beyotime | Cat#C3206 |  |
| ARS staining kit | Solarbio | Cat# G3284 |  |
| MagPure Soil DNA LQ Kit | Magen | Cat# D6356-02 |  |
| Cell Counting Kit-8 Kit | NCM Biotech | Cat#C6005 |  |
| NAD+/NADH Colorimetric Assay Kit | Eabscience | Cat# E-BC-K804-M |  |
| HiScript III RT SuperMix for qPCR (+gDNA wiper) | Vazyme | Cat#R323 |  |
| ATP Assay Kit | Beyotime | Cat#S0026 |  |
| Protease Inhibitor Cocktail | Epizyme | Cat#GRF101 |  |
| Mitochondrial Extraction Kit | Epizyme | Cat#PC205 |  |
| **Software and Algorithms** |  |  |  |
| Integrative Genomics Viewer (IGV) | Broad Institute | https://www.broadinstitute.org/ |  |
| UCSC Genome Browserr | University of California Santa Cruz | http://genome.ucsc.edu/ |  |
| Primer3Plus | N/A | https://www.primer3plus.com/ |  |
| Origin 2021 | OriginLab | https://www.originlab.com/ |  |
| NRecon v1.6 | Bruker | http://www.bruker-microct.com/ |  |
| Dataviewer v1.5.2.4 | Bruker | http://www.bruker-microct.com/ |  |
| CTAn v1.13.8.1 | Bruker | http://www.bruker-microct.com/ |  |
| Mimics Research 19.0 | Materialise | http://www. materialise.com/ |  |
| Prism v9.3.1 | Graphpad | https://www.graphpad.com/;  RRID:SCR_002798 |  |
| Flourish | Flourish Studio | https://flourish.studio/ |  |
| SPSS | IBM | N/A |  |
| Image J | NIH | https://imagej.net/ij/. |  |
